# Supplementary material for: Training and Integration of Eat, Sleep, Console Model for Infants and Families at an Urban Academic Health Center
Source: MedEdPORTAL. 2026 Mar 19;22:11583. doi: 10.15766/mep_2374-8265.11583 (PMC12999543; doi:10.15766/mep_2374-8265.11583)
Supplement: Supplementary file 1 — Eat, Sleep, Console Algorithm.docxEat, Sleep, Console Education.pptxPre- and Postsurvey.docx [file mep_2374-8265.11583-s001.zip › A. Eat, Sleep, Console Algorithm.docx]

Key

**ESC**- Eat, Sleep, Console

**NOWS**- Neonatal Opioid Withdrawal Syndrome

**RN**- Registered Nurse

**APP**- Advanced Practice Provider

**NCU**- Neonatal Care Unit

Appendix A: Eat, Sleep, Console Algorithm

Begin ESC assessment within 4 hours of birth

- Poor eating due to NOWS?
- Slept less than 1hr due to NOWS?
- Unable to be consoled within 10mins due to NOWS?

Yes= 1 point each

Repeated score of 2 or higher after first trial dose of morphine

Team huddle with parent, APP, RN, and charge RN. Consider second x1 trial dose of morphine.

Consider scheduled q3h morphine doses after 3 consecutive trial doses in 24hrs.

Score 3 or score 1 or 2 for two consecutive scoring periods

Team huddle with parent, APP, RN, and charge RN.

Consider X1 trial dose of morphine and transfer to NCU for 4hrs for pulse oximetry monitoring.

Score 2

Team huddle with parent, APP, RN, and charge RN to discuss treatment options.

Score 1

Team huddle with parent and RN to optimize non-pharmacologic interventions.

Score 0

No intervention needed. Infant is tolerating withdrawal symptoms.

Re-assess after every feeding or at least every 4hrs

Stop ESC assessments after 96hrs of no pharmacologic treatment for withdrawal
