# Supplementary material for: Training and Integration of Eat, Sleep, Console Model for Infants and Families at an Urban Academic Health Center
Source: MedEdPORTAL. 2026 Mar 19;22:11583. doi: 10.15766/mep_2374-8265.11583 (PMC12999543; doi:10.15766/mep_2374-8265.11583)
Supplement: Supplementary file 1 — Eat, Sleep, Console Algorithm.docxEat, Sleep, Console Education.pptxPre- and Postsurvey.docx [file mep_2374-8265.11583-s001.zip › B. Eat, Sleep, Console Education.pptx]

## Slide 1
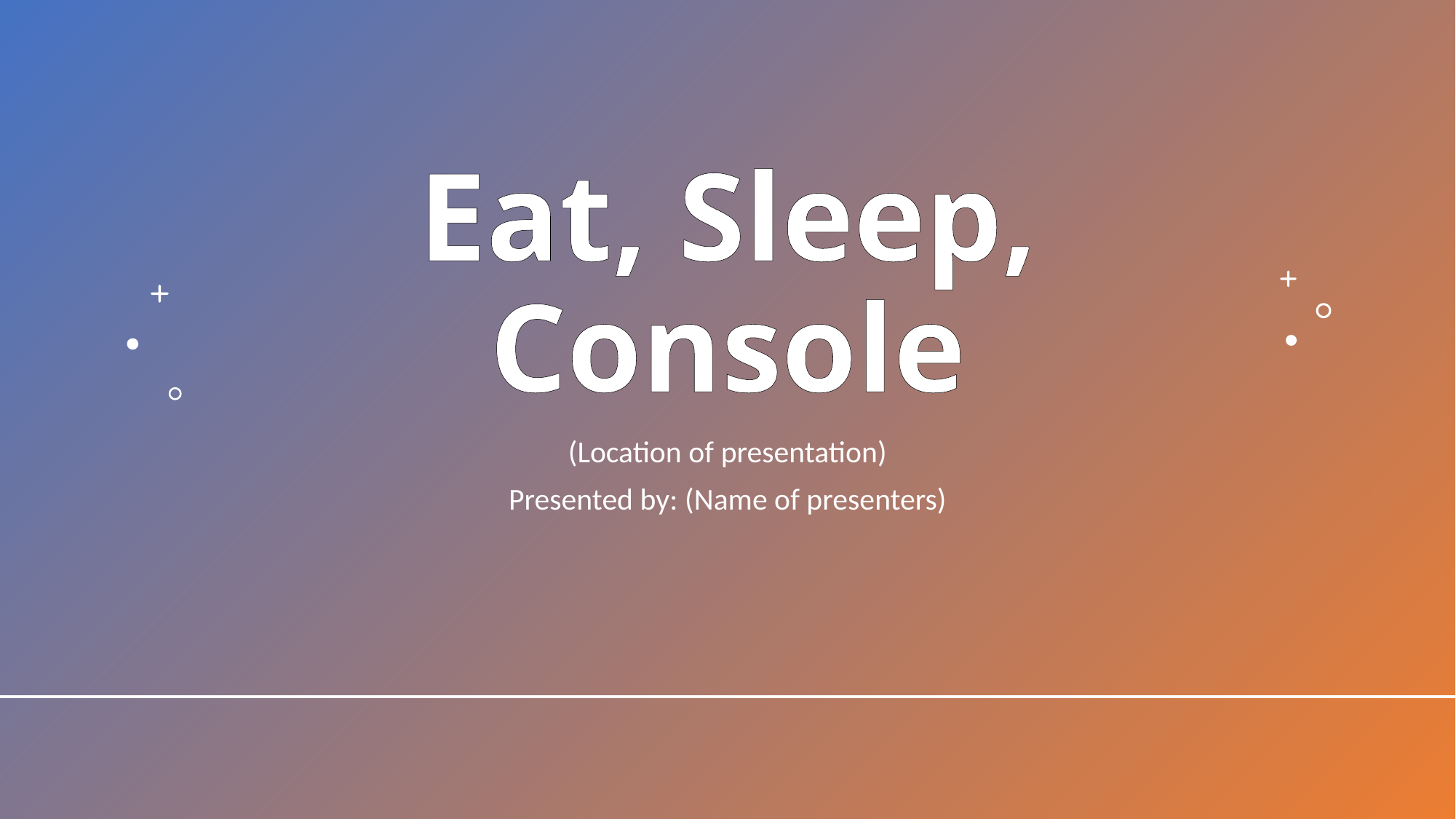

# Eat, Sleep, Console
(Location of presentation)
Presented by: (Name of presenters)

## Slide 2
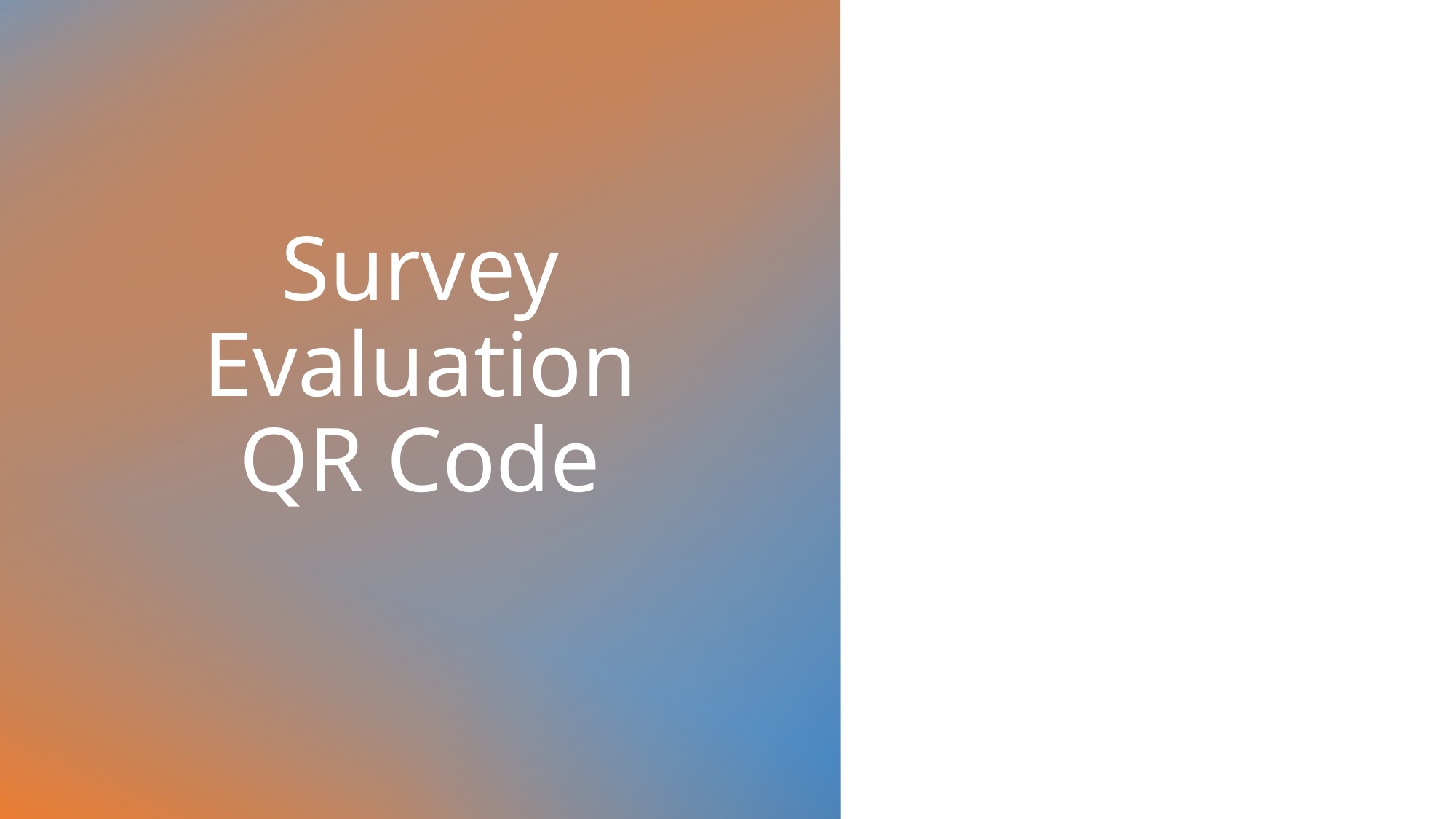

# Survey Evaluation QR Code

## Slide 3
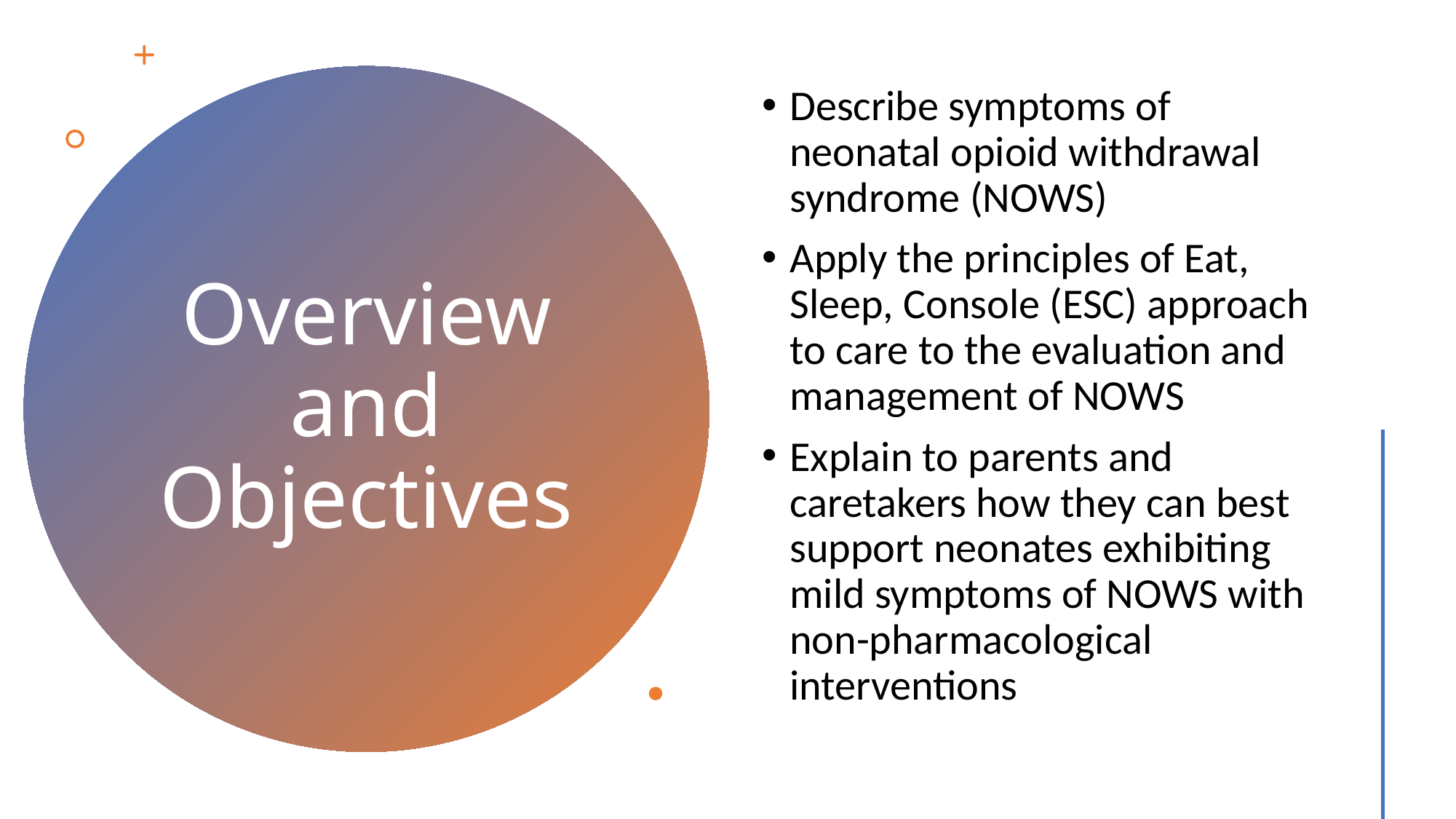

Describe symptoms of neonatal opioid withdrawal syndrome (NOWS)
Apply the principles of Eat, Sleep, Console (ESC) approach to care to the evaluation and management of NOWS
Explain to parents and caretakers how they can best support neonates exhibiting mild symptoms of NOWS with non-pharmacological interventions
# Overview and Objectives

## Slide 4
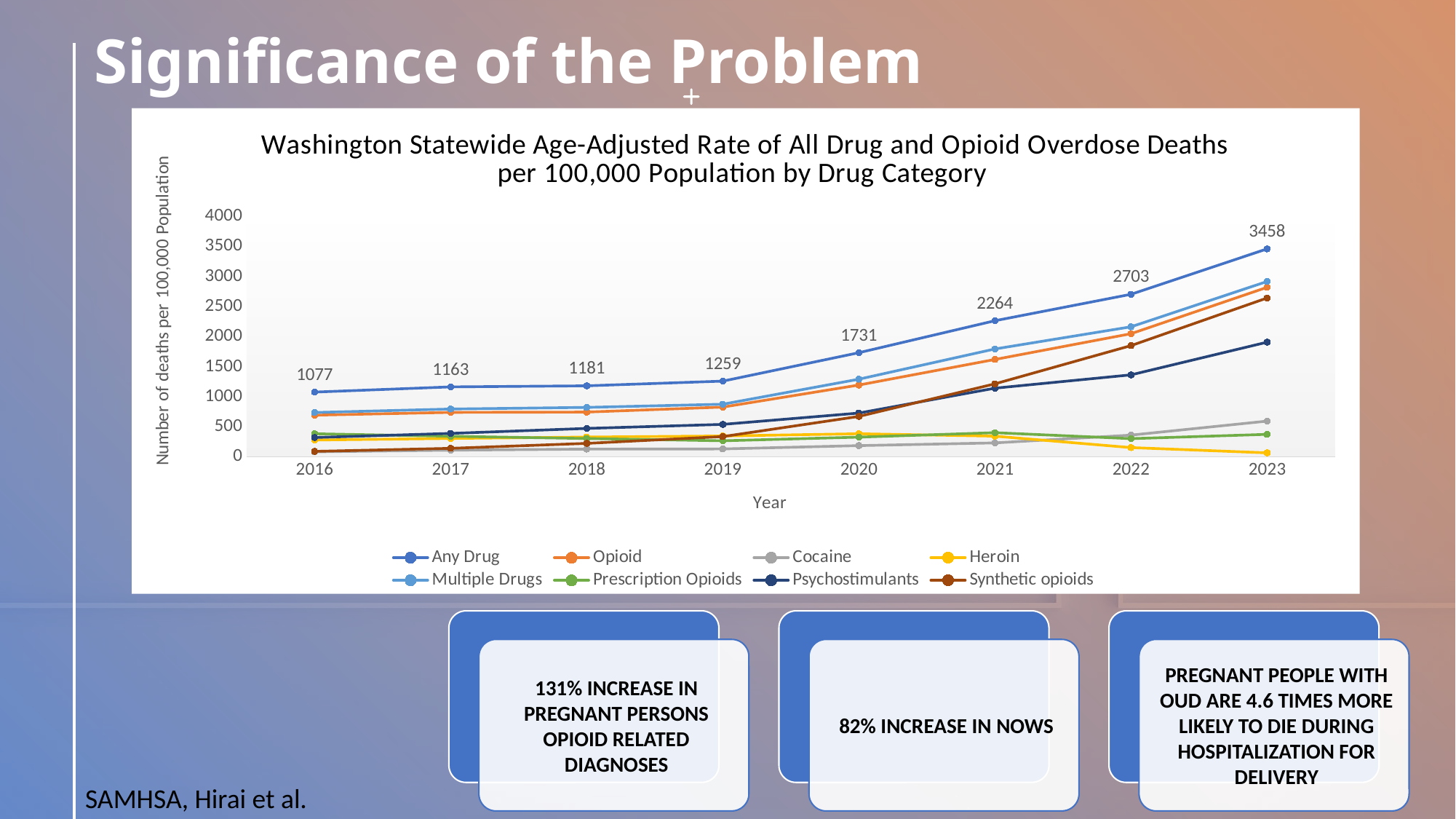

# Significance of the Problem
### Chart: Washington Statewide Age-Adjusted Rate of All Drug and Opioid Overdose Deaths per 100,000 Population by Drug Category
| Category | Any Drug | Opioid | Cocaine | Heroin | Multiple Drugs | Prescription Opioids | Psychostimulants | Synthetic opioids |
|---|---|---|---|---|---|---|---|---|
| 2016 | 1077.0 | 693.0 | 84.0 | 280.0 | 737.0 | 384.0 | 321.0 | 91.0 |
| 2017 | 1163.0 | 739.0 | 111.0 | 306.0 | 795.0 | 342.0 | 390.0 | 142.0 |
| 2018 | 1181.0 | 744.0 | 129.0 | 329.0 | 822.0 | 305.0 | 473.0 | 224.0 |
| 2019 | 1259.0 | 827.0 | 132.0 | 347.0 | 875.0 | 267.0 | 540.0 | 337.0 |
| 2020 | 1731.0 | 1194.0 | 187.0 | 384.0 | 1291.0 | 328.0 | 728.0 | 672.0 |
| 2021 | 2264.0 | 1619.0 | 232.0 | 344.0 | 1794.0 | 402.0 | 1142.0 | 1214.0 |
| 2022 | 2703.0 | 2048.0 | 361.0 | 154.0 | 2163.0 | 303.0 | 1363.0 | 1850.0 |
| 2023 | 3458.0 | 2819.0 | 594.0 | 67.0 | 2916.0 | 374.0 | 1907.0 | 2642.0 |
SAMHSA, Hirai et al.

## Slide 5
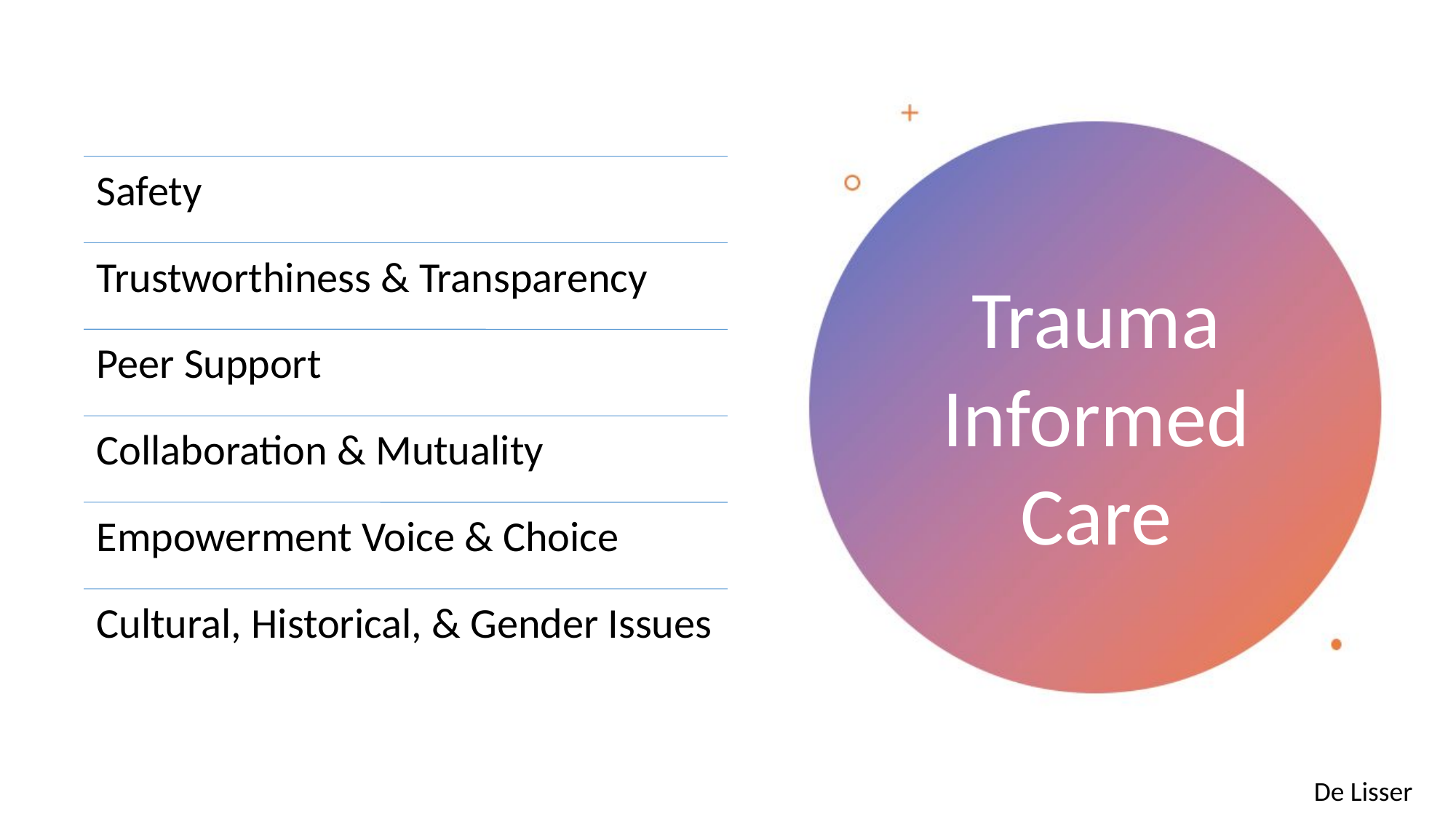

Trauma Informed Care
De Lisser

## Slide 6
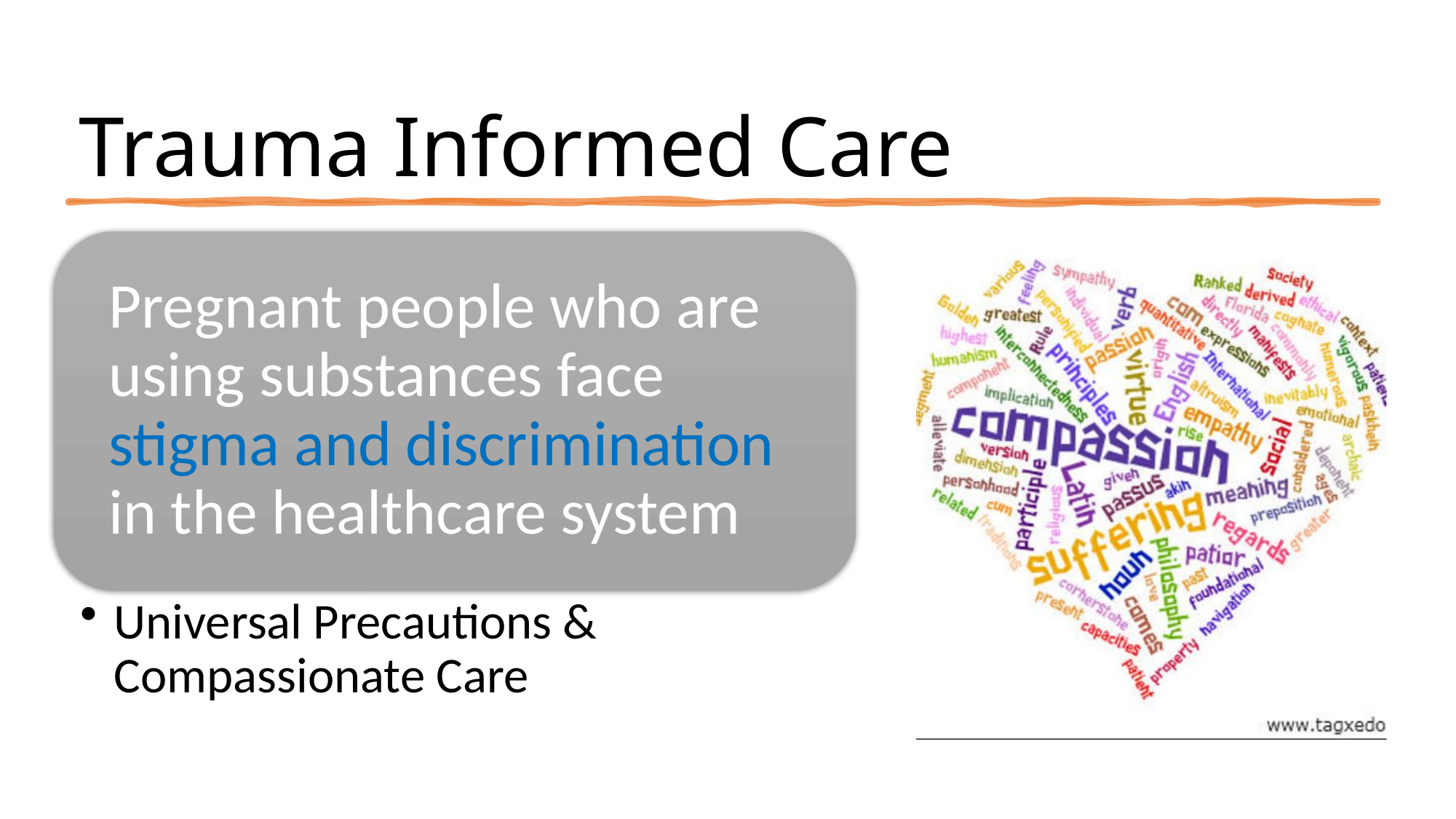

# Trauma Informed Care

## Slide 7
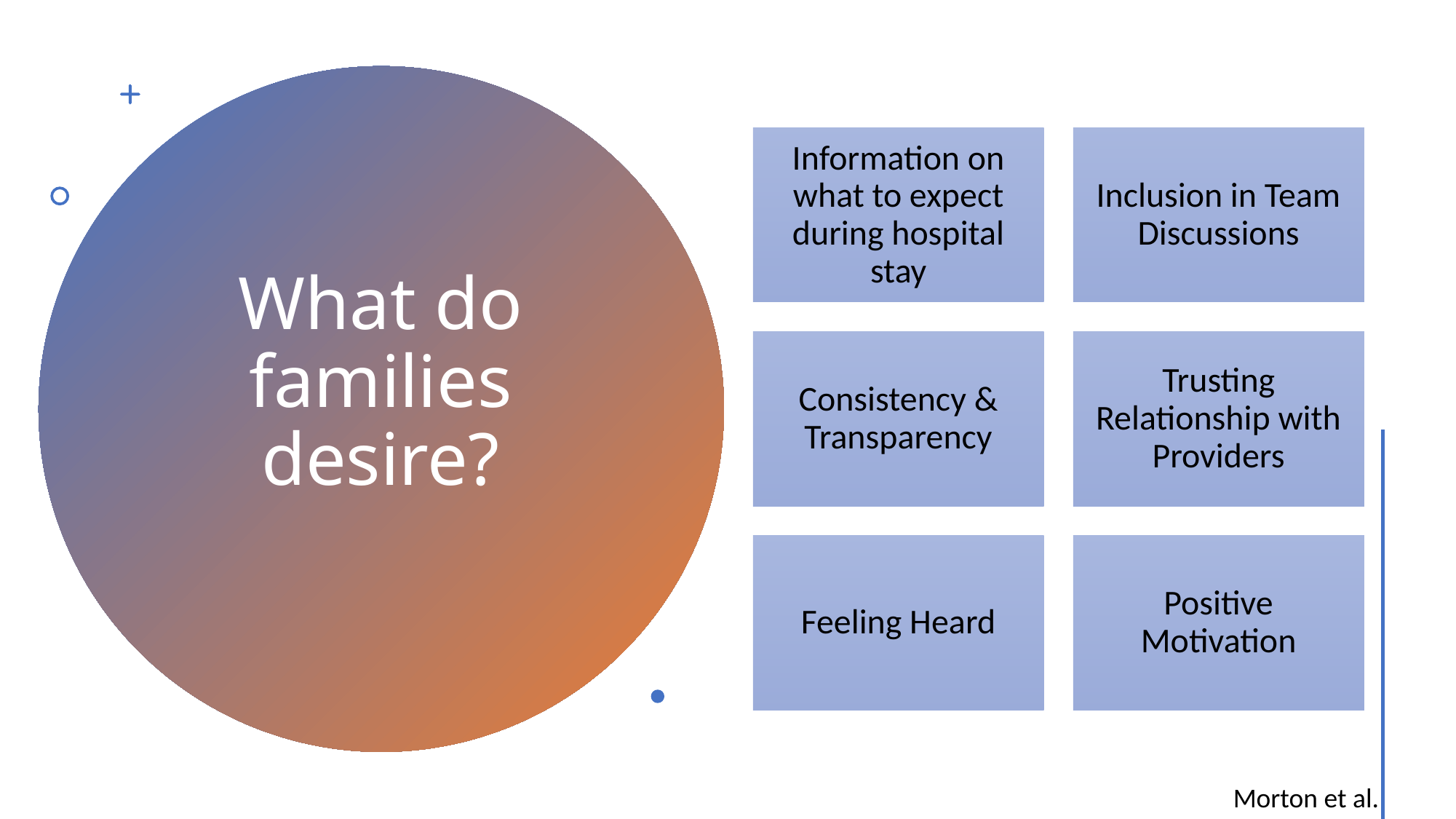

# What do families desire?
Morton et al.

## Slide 8
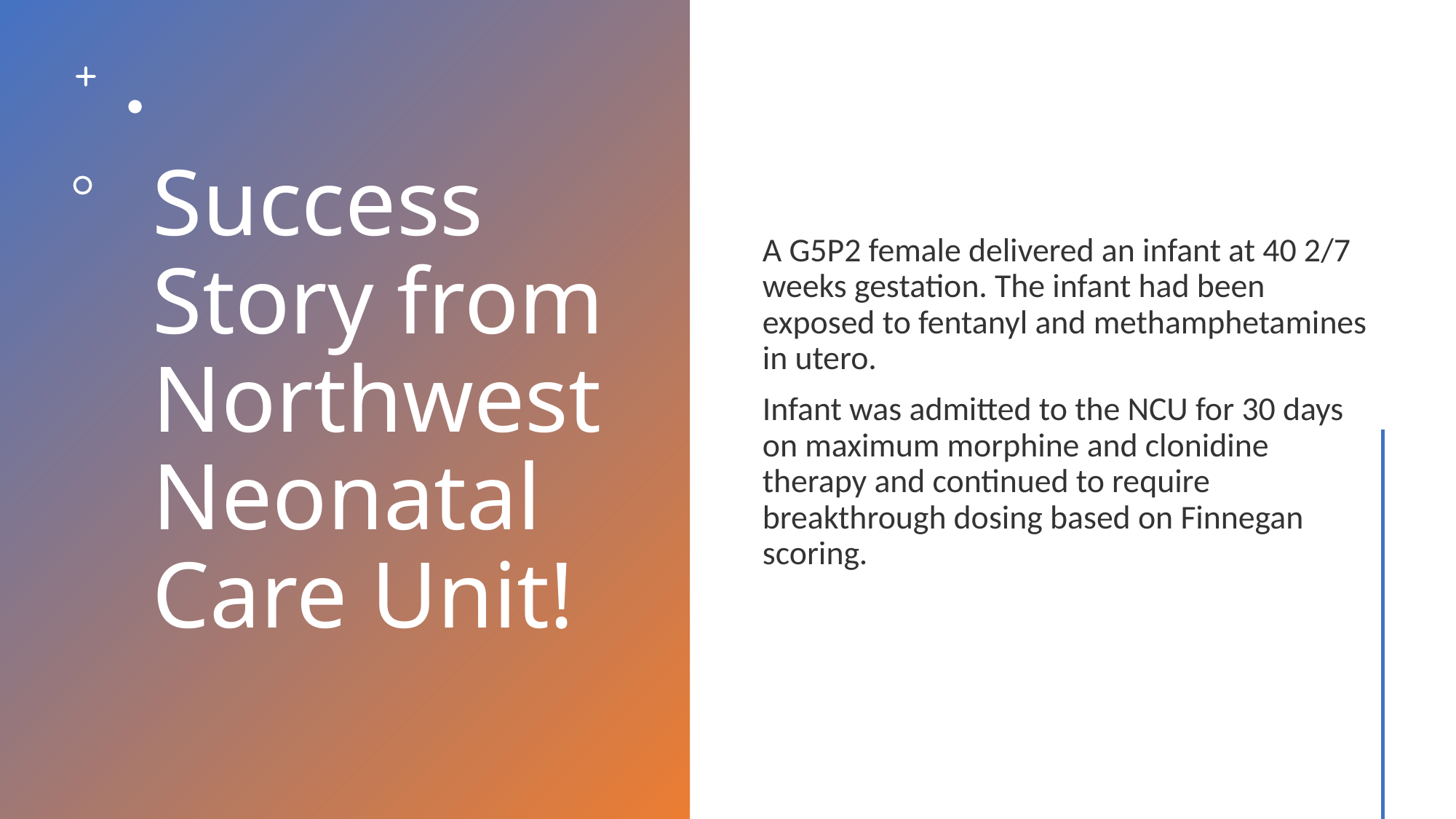

# Success Story from Northwest Neonatal Care Unit!
A G5P2 female delivered an infant at 40 2/7 weeks gestation. The infant had been exposed to fentanyl and methamphetamines in utero.
Infant was admitted to the NCU for 30 days on maximum morphine and clonidine therapy and continued to require  breakthrough dosing based on Finnegan scoring.

## Slide 9
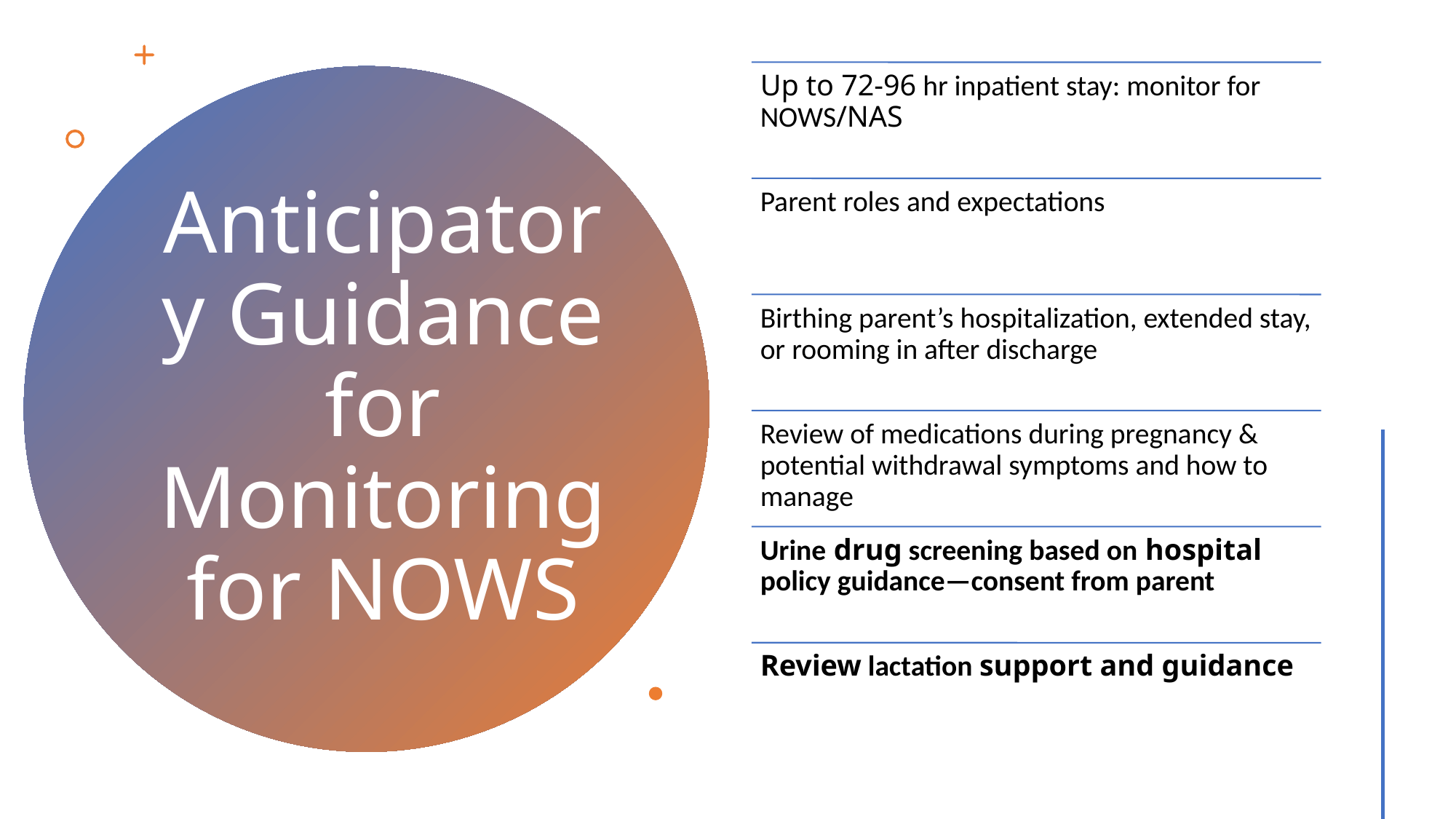

# Anticipatory Guidance for Monitoring for NOWS

## Slide 10
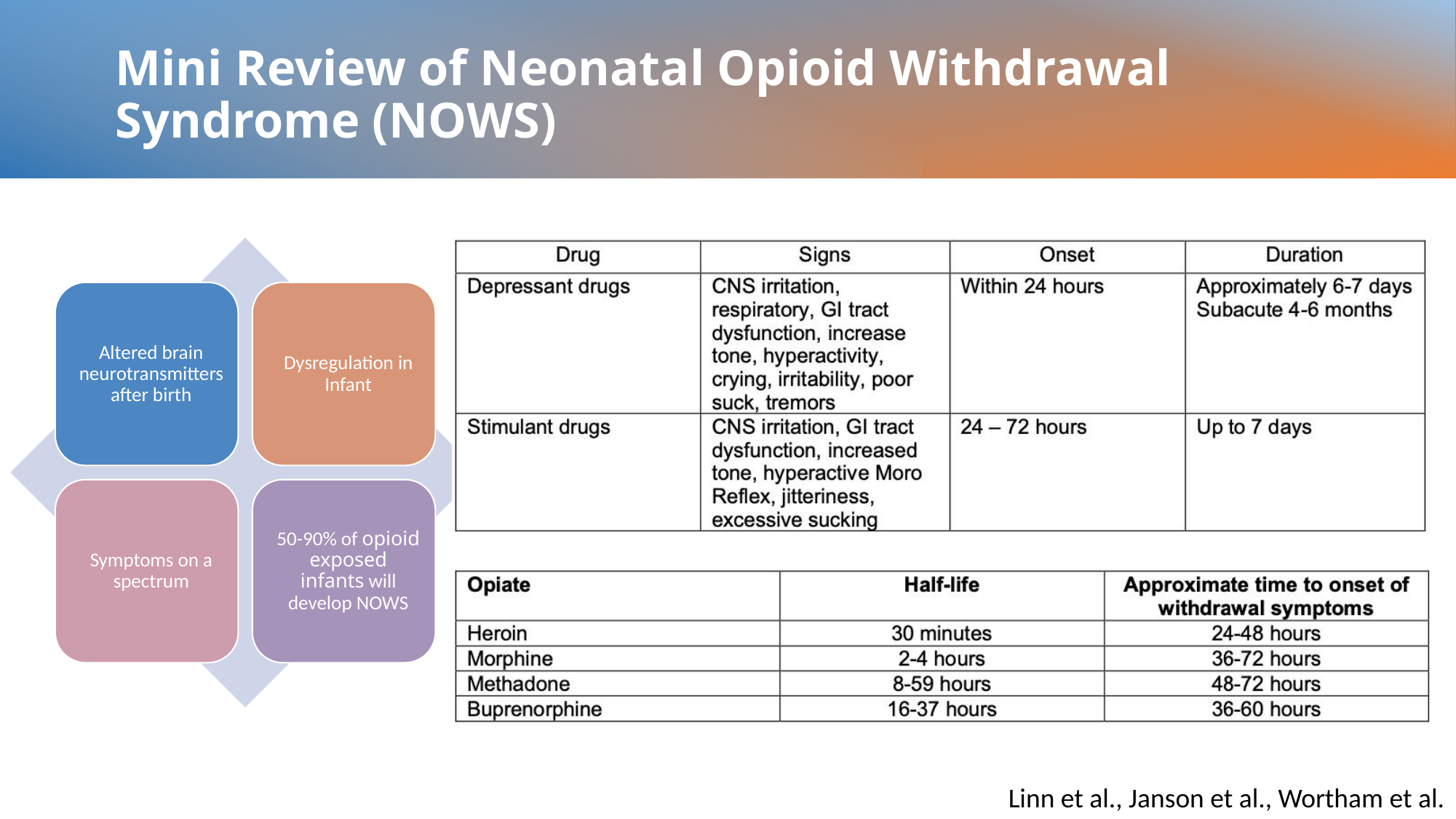

# Mini Review of Neonatal Opioid Withdrawal Syndrome (NOWS)
Linn et al., Janson et al., Wortham et al.

## Slide 11
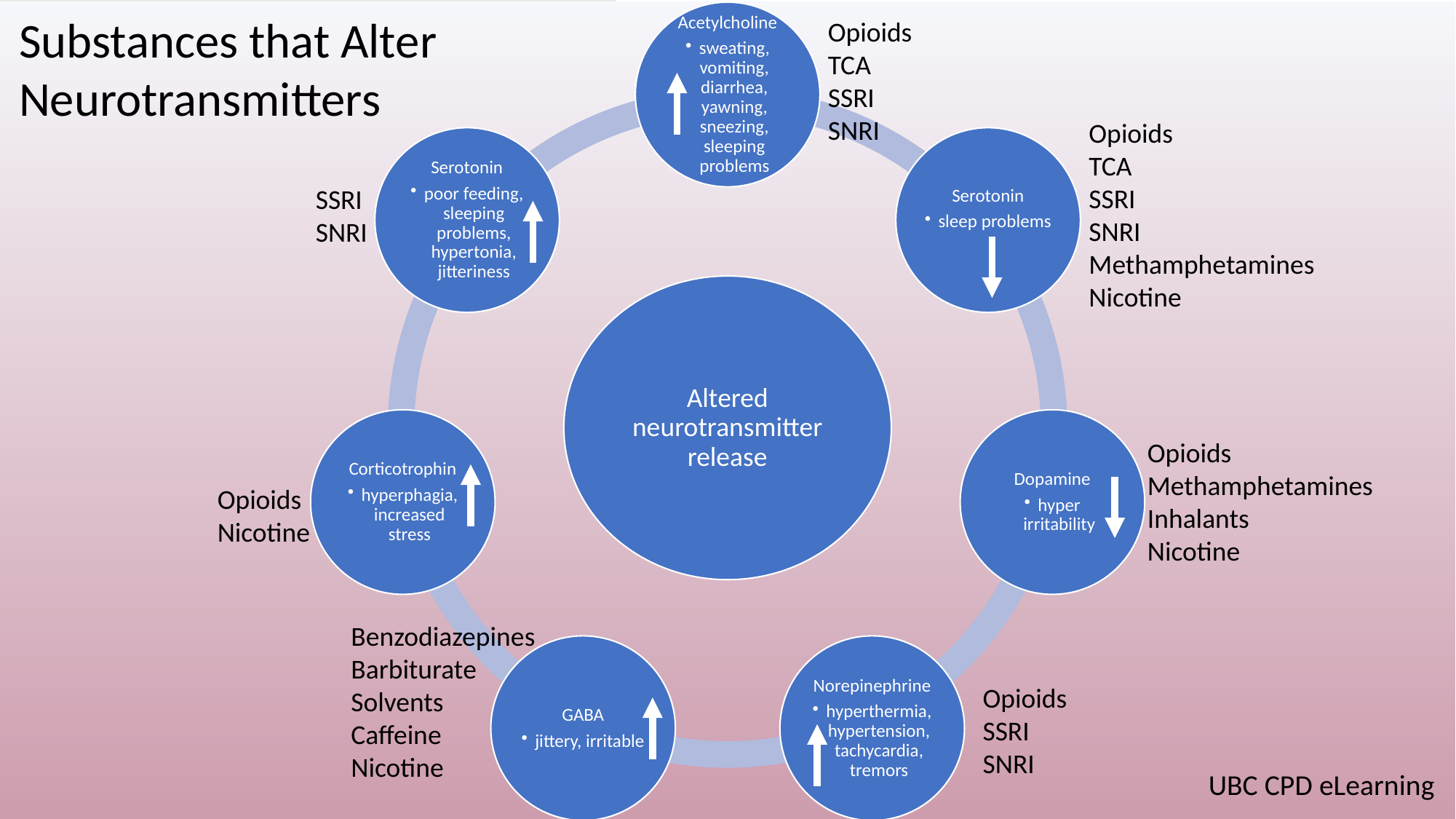

Substances that Alter Neurotransmitters
Opioids
TCA
SSRI
SNRI
Opioids
TCA
SSRI
SNRI
Methamphetamines
Nicotine
SSRI
SNRI
Opioids
Methamphetamines
Inhalants
Nicotine
Opioids
Nicotine
Benzodiazepines
Barbiturate
Solvents
Caffeine
Nicotine
Opioids
SSRI
SNRI
UBC CPD eLearning

## Slide 12
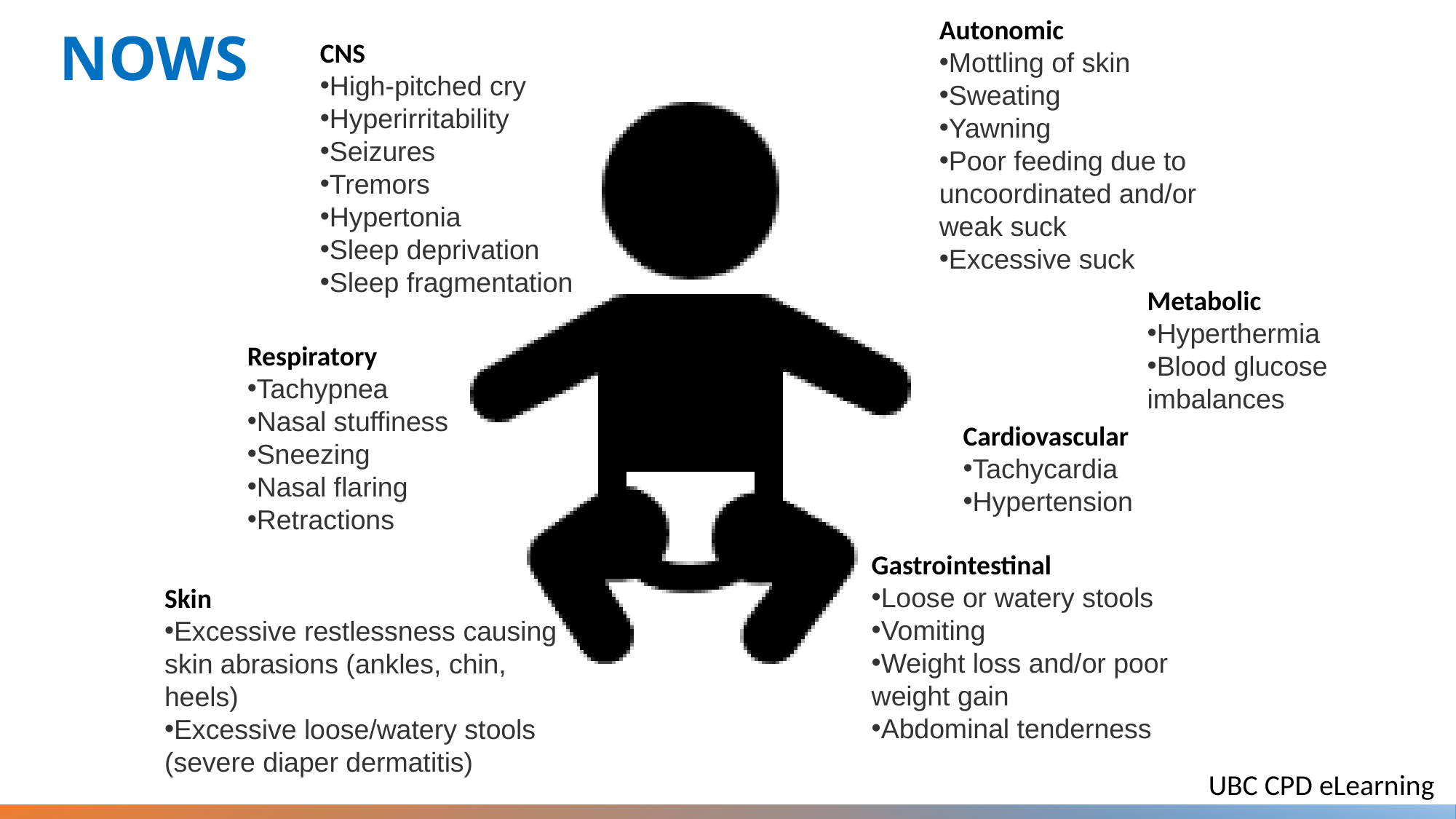

Autonomic
Mottling of skin
Sweating
Yawning
Poor feeding due to uncoordinated and/or weak suck
Excessive suck
# NOWS
CNS
High-pitched cry
Hyperirritability
Seizures
Tremors
Hypertonia
Sleep deprivation
Sleep fragmentation
Metabolic
Hyperthermia
Blood glucose imbalances
Respiratory
Tachypnea
Nasal stuffiness
Sneezing
Nasal flaring
Retractions
Cardiovascular
Tachycardia
Hypertension
Gastrointestinal
Loose or watery stools
Vomiting
Weight loss and/or poor weight gain
Abdominal tenderness
Skin
Excessive restlessness causing skin abrasions (ankles, chin, heels)
Excessive loose/watery stools (severe diaper dermatitis)
UBC CPD eLearning

## Slide 13
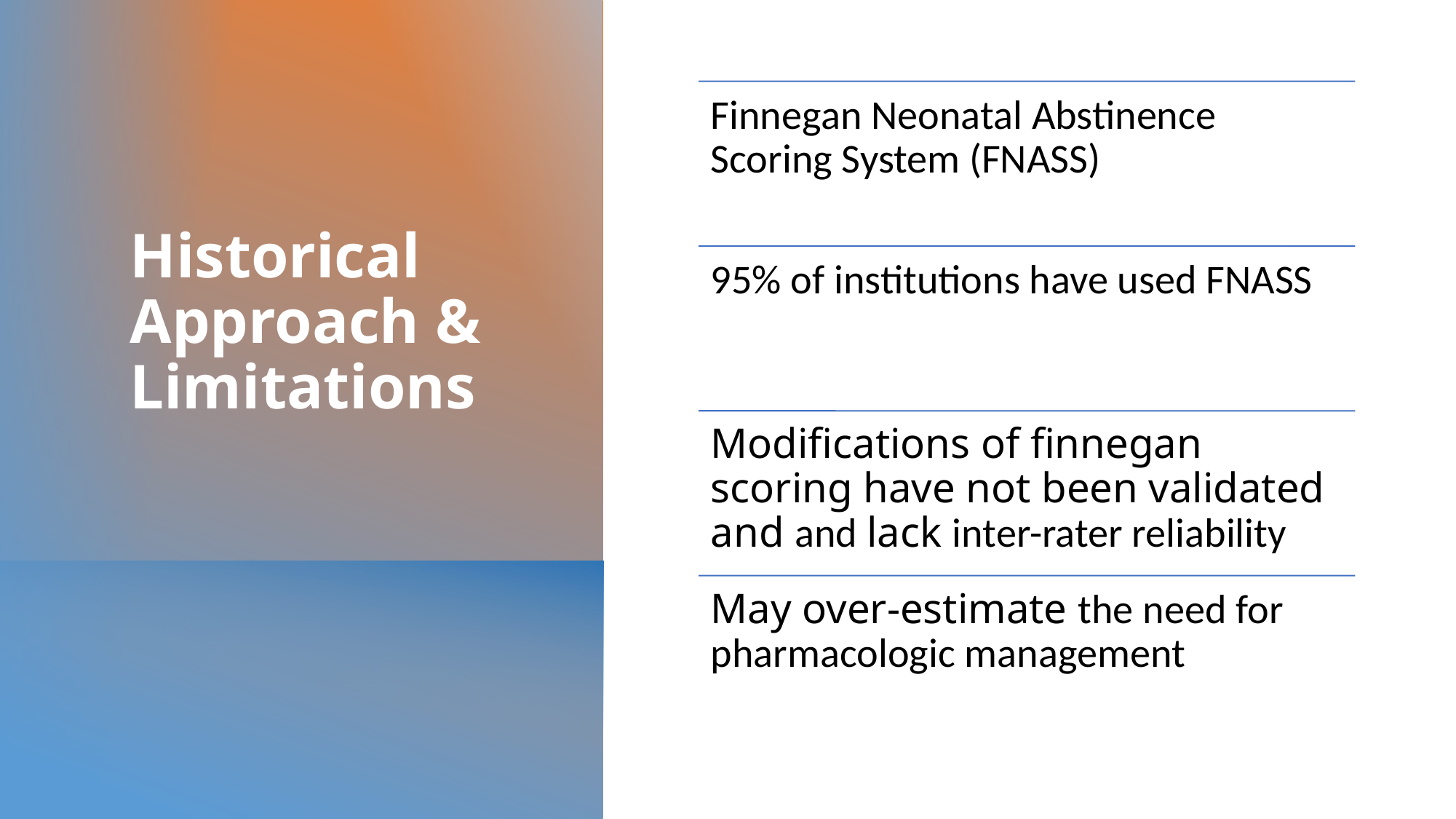

# Historical Approach & Limitations

## Slide 14
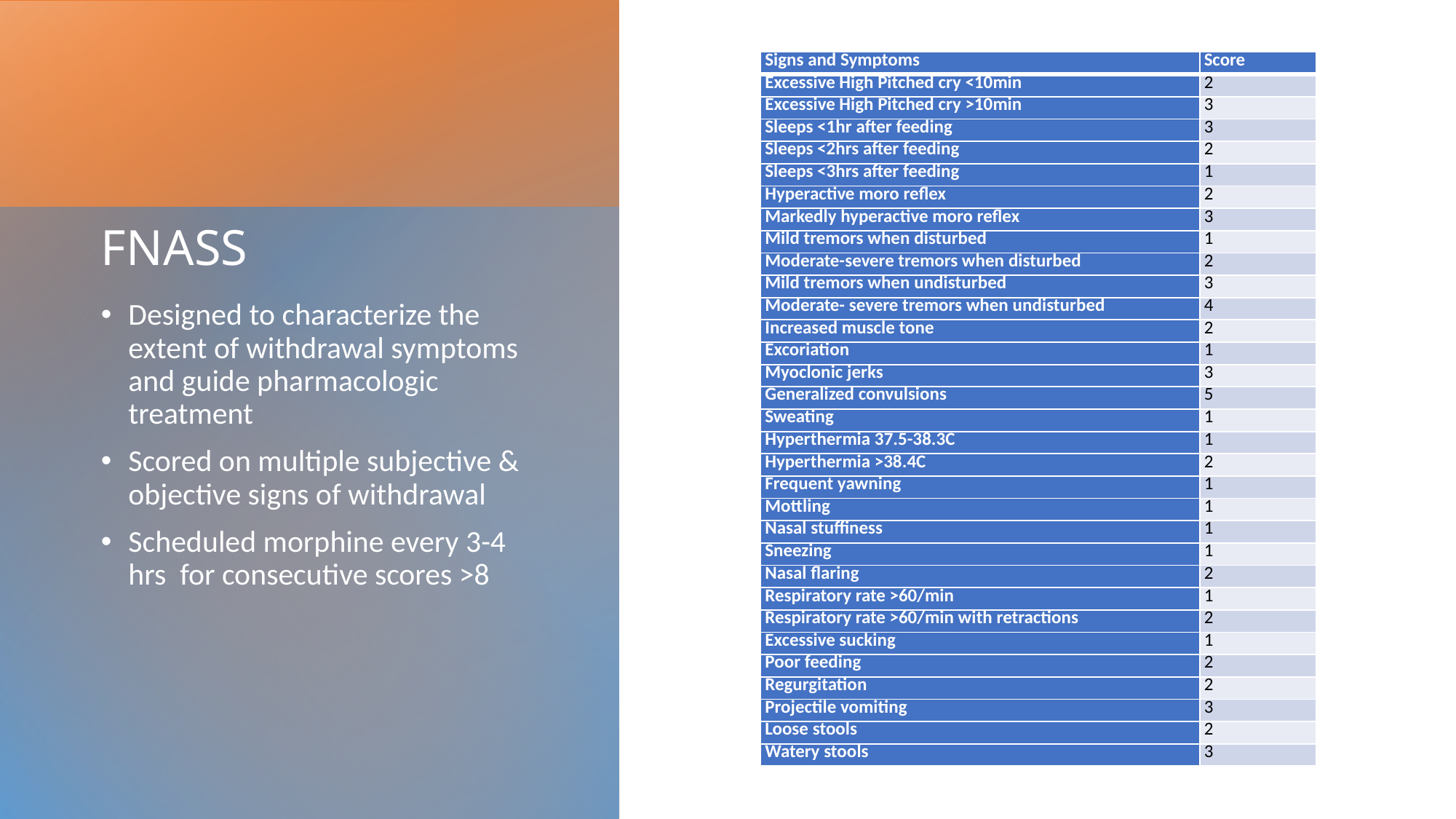

| Signs and Symptoms | Score |
| --- | --- |
| Excessive High Pitched cry <10min | 2 |
| Excessive High Pitched cry >10min | 3 |
| Sleeps <1hr after feeding | 3 |
| Sleeps <2hrs after feeding | 2 |
| Sleeps <3hrs after feeding | 1 |
| Hyperactive moro reflex | 2 |
| Markedly hyperactive moro reflex | 3 |
| Mild tremors when disturbed | 1 |
| Moderate-severe tremors when disturbed | 2 |
| Mild tremors when undisturbed | 3 |
| Moderate- severe tremors when undisturbed | 4 |
| Increased muscle tone | 2 |
| Excoriation | 1 |
| Myoclonic jerks | 3 |
| Generalized convulsions | 5 |
| Sweating | 1 |
| Hyperthermia 37.5-38.3C | 1 |
| Hyperthermia >38.4C | 2 |
| Frequent yawning | 1 |
| Mottling | 1 |
| Nasal stuffiness | 1 |
| Sneezing | 1 |
| Nasal flaring | 2 |
| Respiratory rate >60/min | 1 |
| Respiratory rate >60/min with retractions | 2 |
| Excessive sucking | 1 |
| Poor feeding | 2 |
| Regurgitation | 2 |
| Projectile vomiting | 3 |
| Loose stools | 2 |
| Watery stools | 3 |
# FNASS
Designed to characterize the extent of withdrawal symptoms and guide pharmacologic treatment
Scored on multiple subjective & objective signs of withdrawal
Scheduled morphine every 3-4 hrs  for consecutive scores >8

## Slide 15
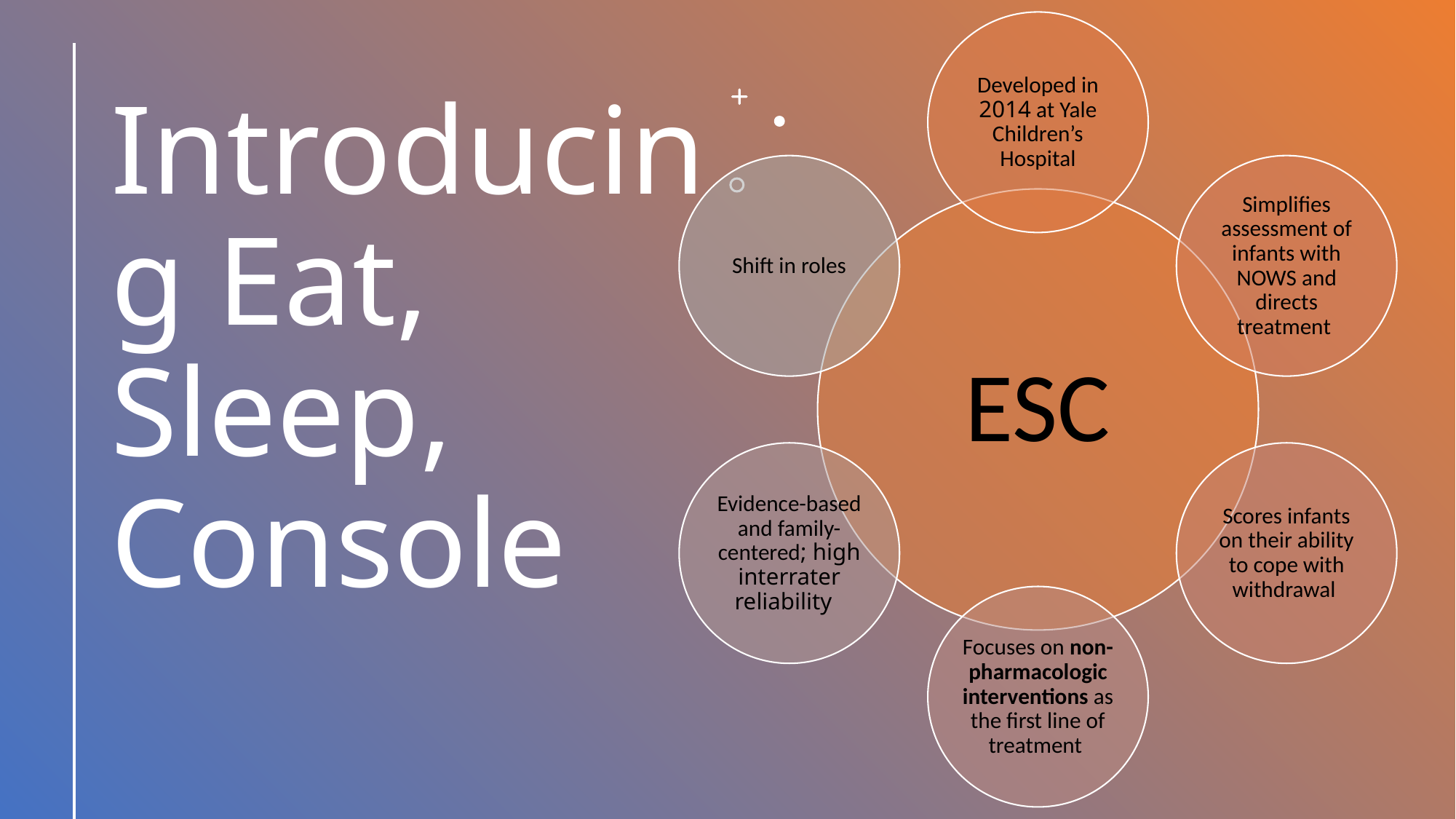

# Introducing Eat, Sleep, Console

## Slide 16
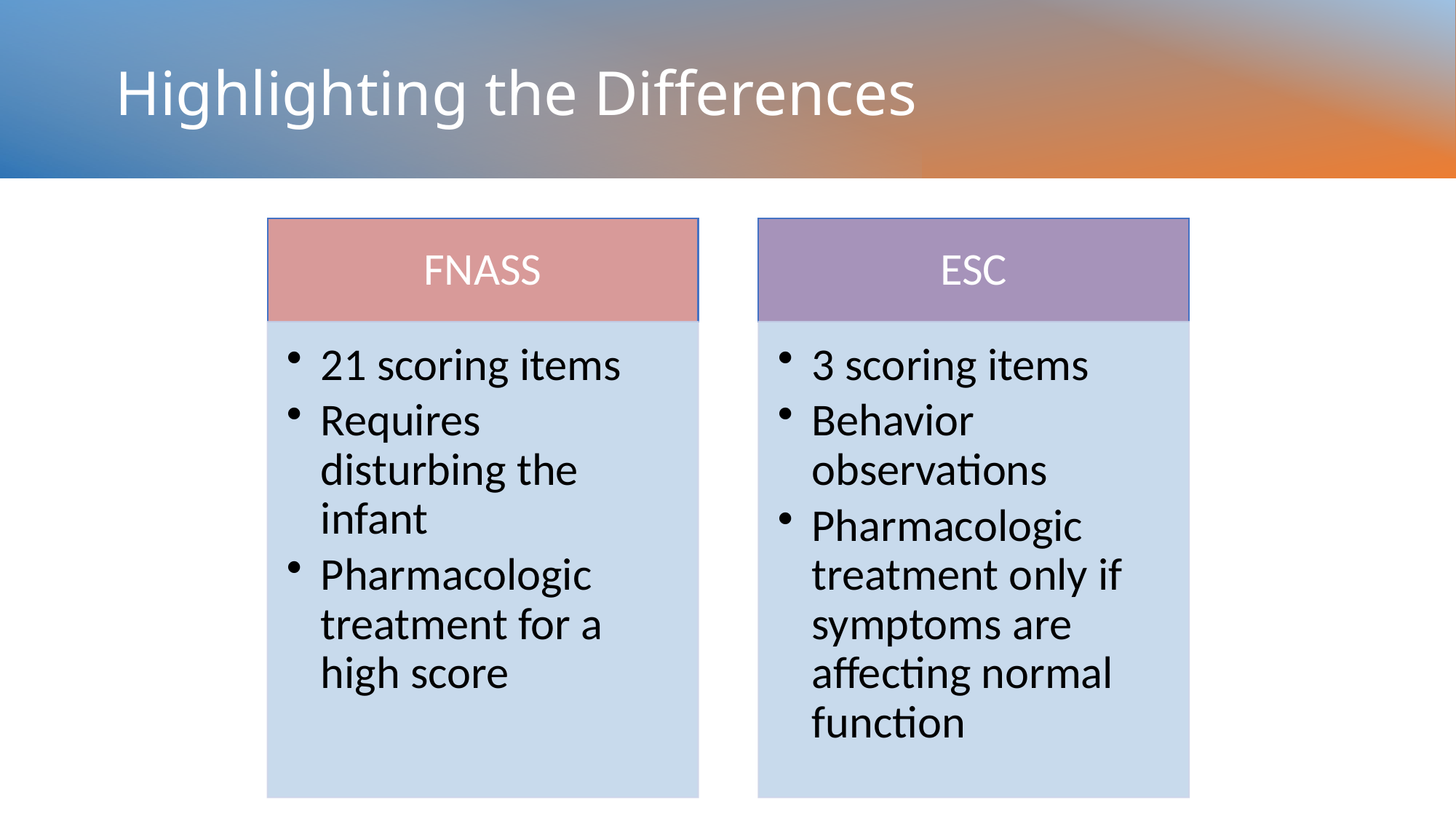

# Highlighting the Differences

## Slide 17
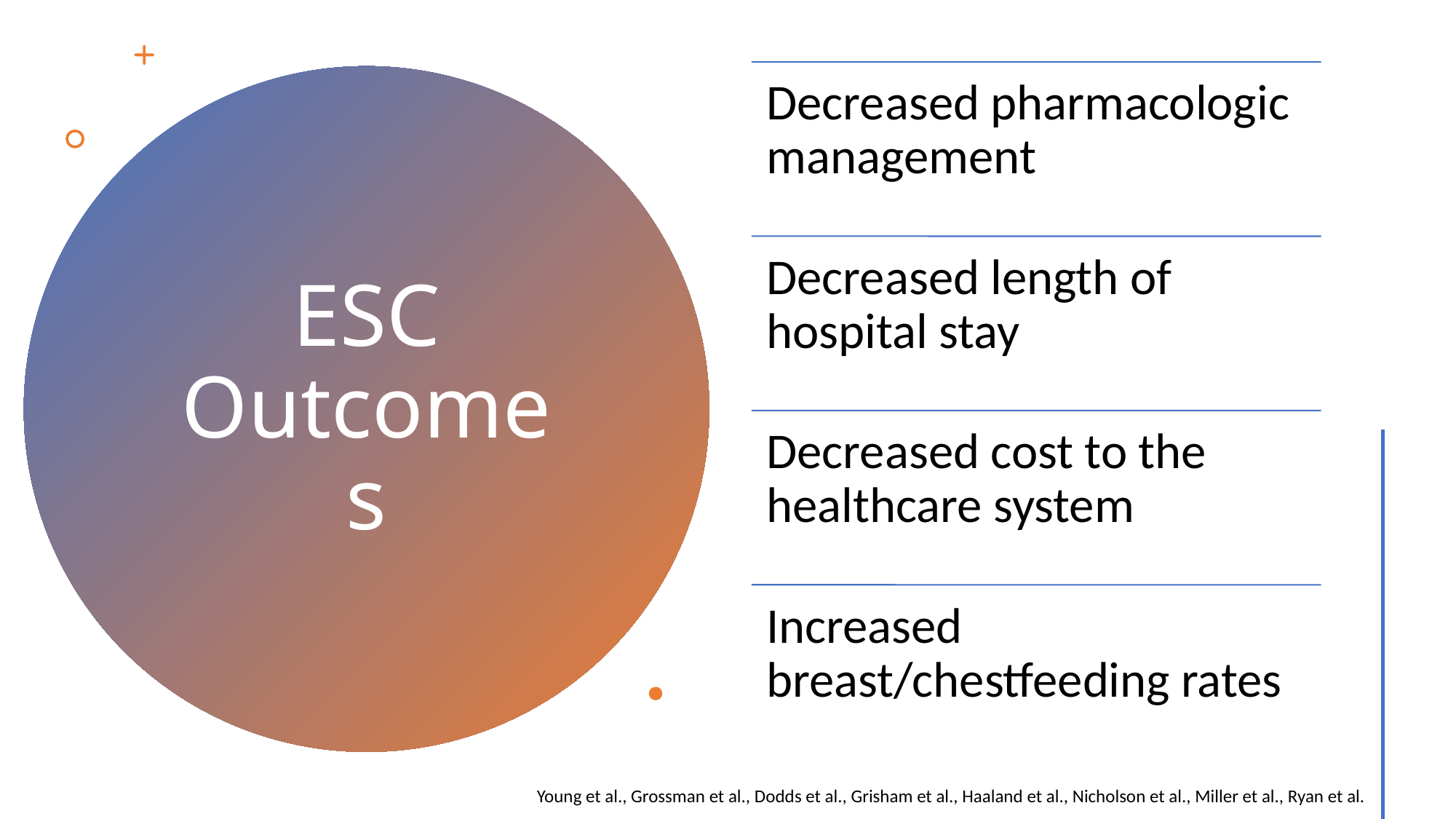

# ESC Outcomes
Young et al., Grossman et al., Dodds et al., Grisham et al., Haaland et al., Nicholson et al., Miller et al., Ryan et al.

## Slide 18
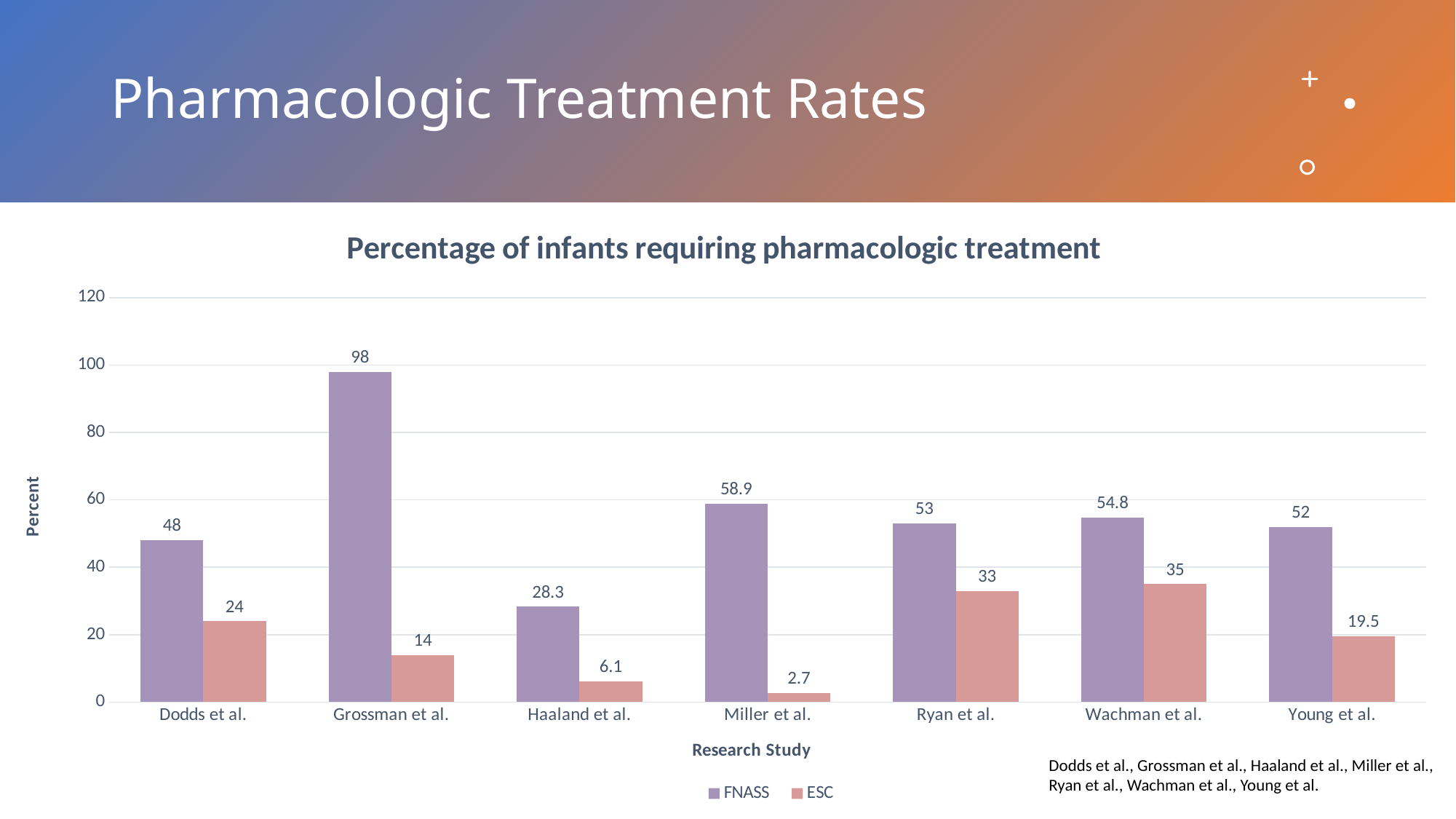

# Pharmacologic Treatment Rates
### Chart: Percentage of infants requiring pharmacologic treatment
| Category | FNASS | ESC |
|---|---|---|
| Dodds et al. | 48.0 | 24.0 |
| Grossman et al. | 98.0 | 14.0 |
| Haaland et al. | 28.3 | 6.1 |
| Miller et al. | 58.9 | 2.7 |
| Ryan et al. | 53.0 | 33.0 |
| Wachman et al. | 54.8 | 35.0 |
| Young et al. | 52.0 | 19.5 |Dodds et al., Grossman et al., Haaland et al., Miller et al., Ryan et al., Wachman et al., Young et al.

## Slide 19
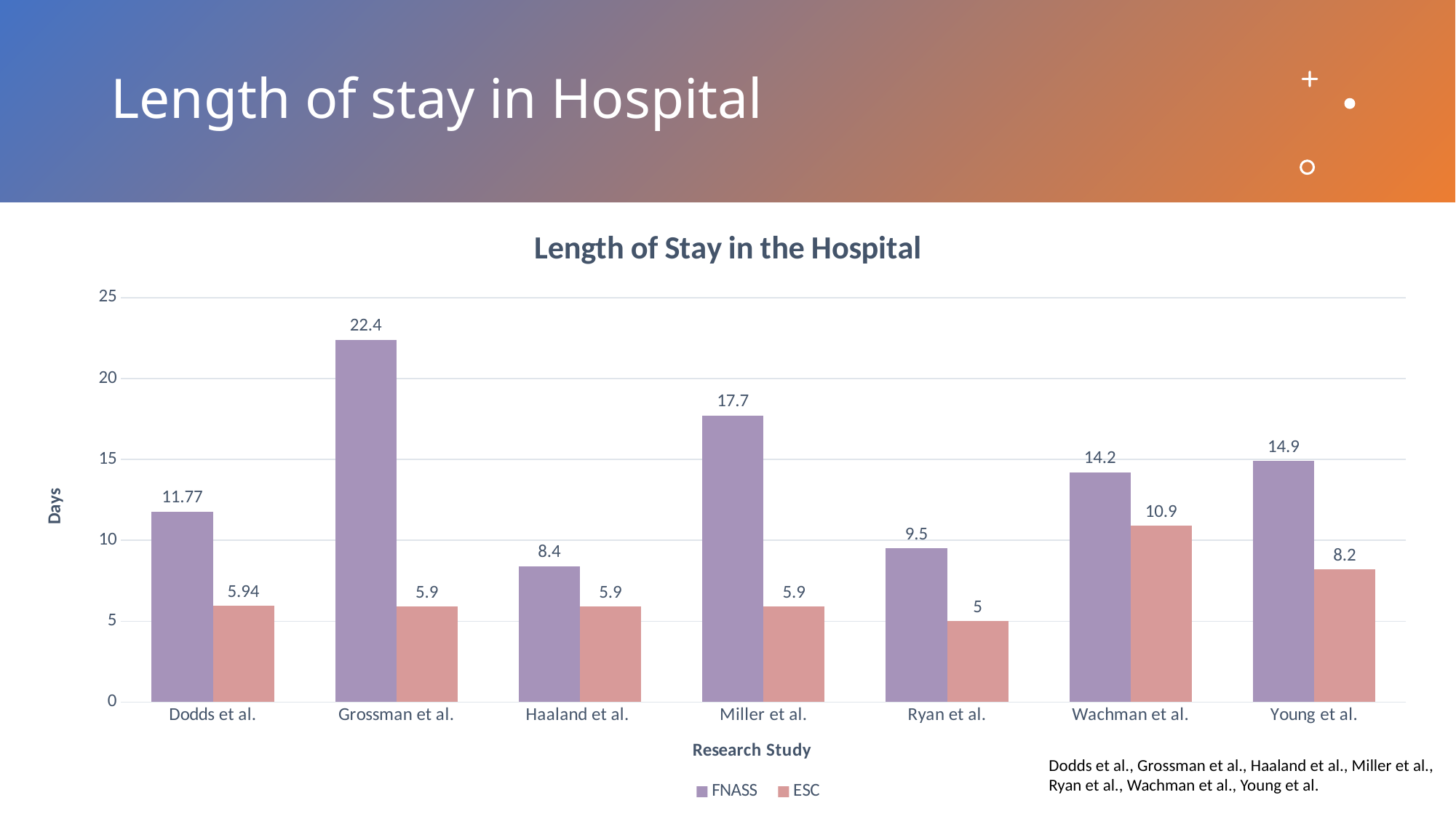

# Length of stay in Hospital
### Chart: Length of Stay in the Hospital
| Category | FNASS | ESC |
|---|---|---|
| Dodds et al. | 11.77 | 5.94 |
| Grossman et al. | 22.4 | 5.9 |
| Haaland et al. | 8.4 | 5.9 |
| Miller et al. | 17.7 | 5.9 |
| Ryan et al. | 9.5 | 5.0 |
| Wachman et al. | 14.2 | 10.9 |
| Young et al. | 14.9 | 8.2 |Dodds et al., Grossman et al., Haaland et al., Miller et al., Ryan et al., Wachman et al., Young et al.

## Slide 20
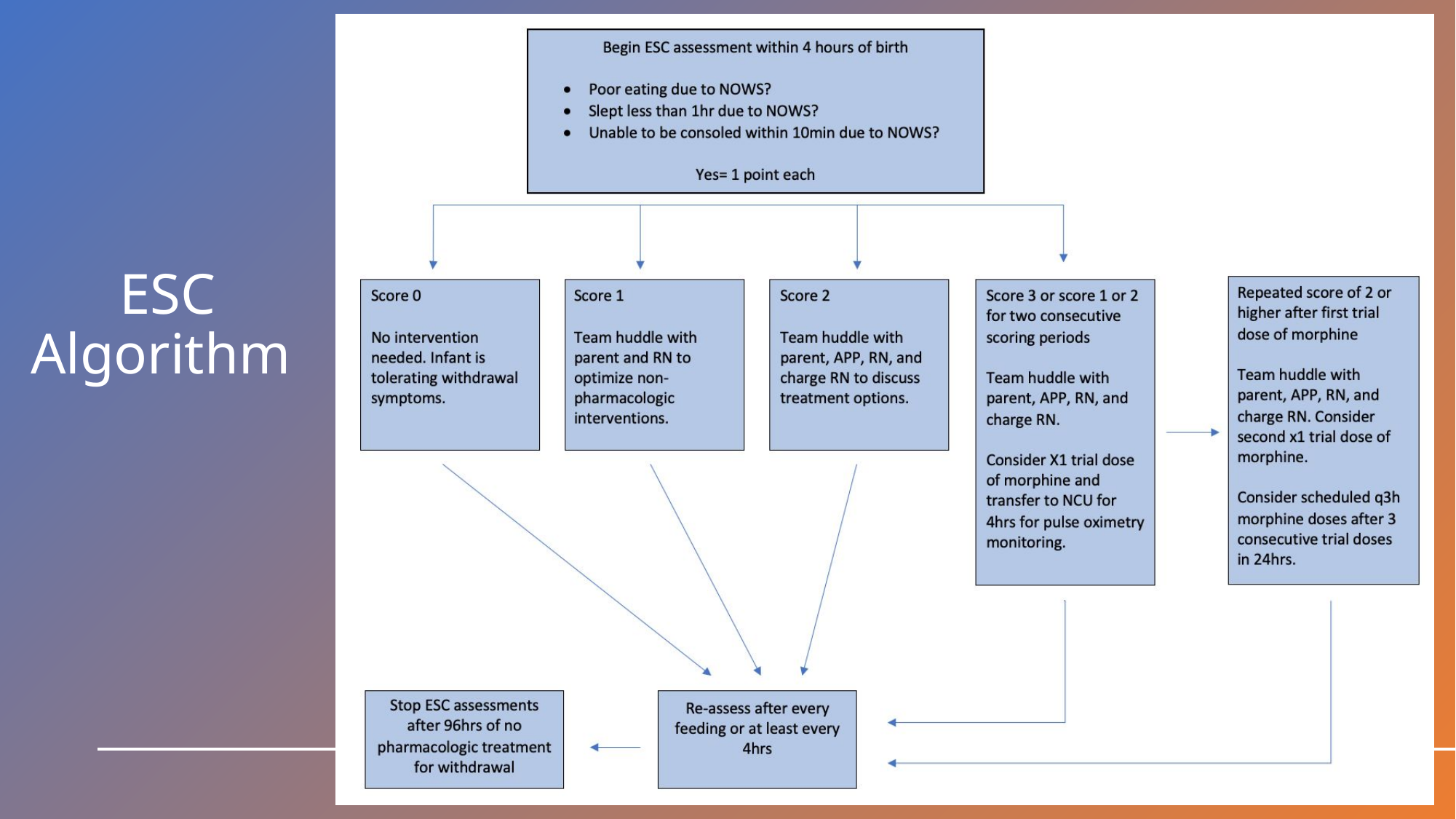

# ESC Algorithm

## Slide 21
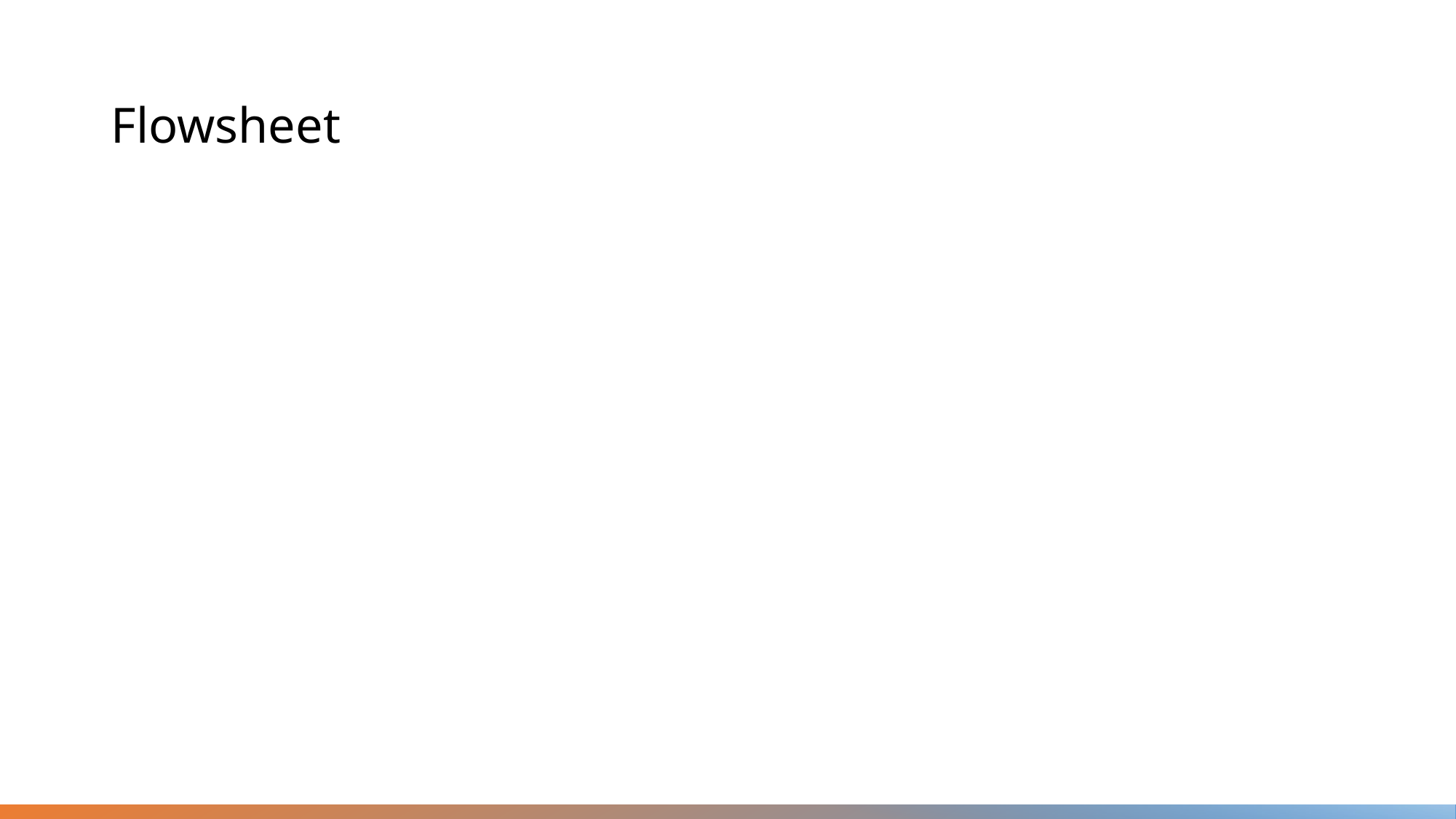

# Flowsheet

## Slide 22
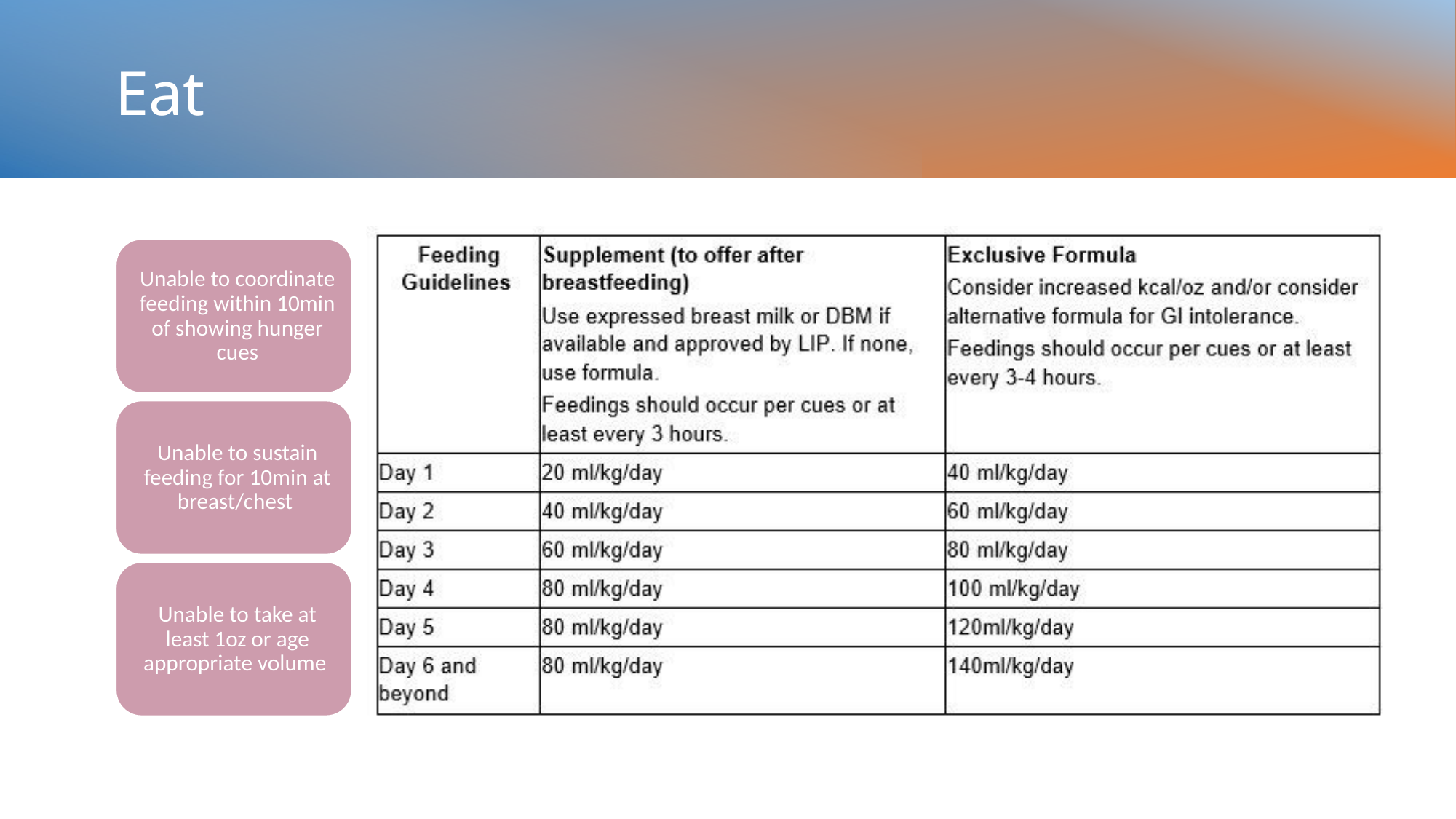

# Eat

## Slide 23
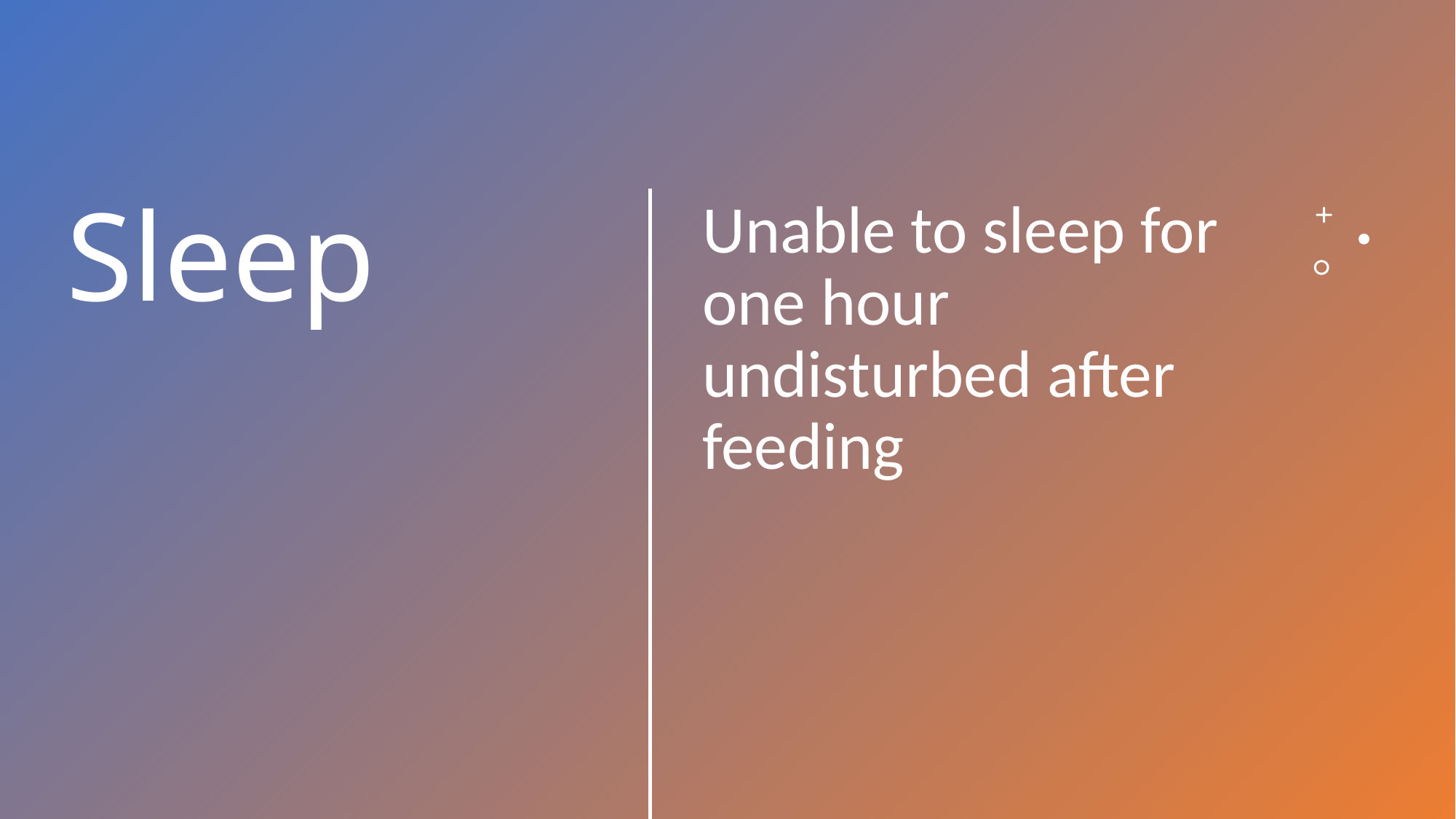

Unable to sleep for one hour undisturbed after feeding
# Sleep

## Slide 24
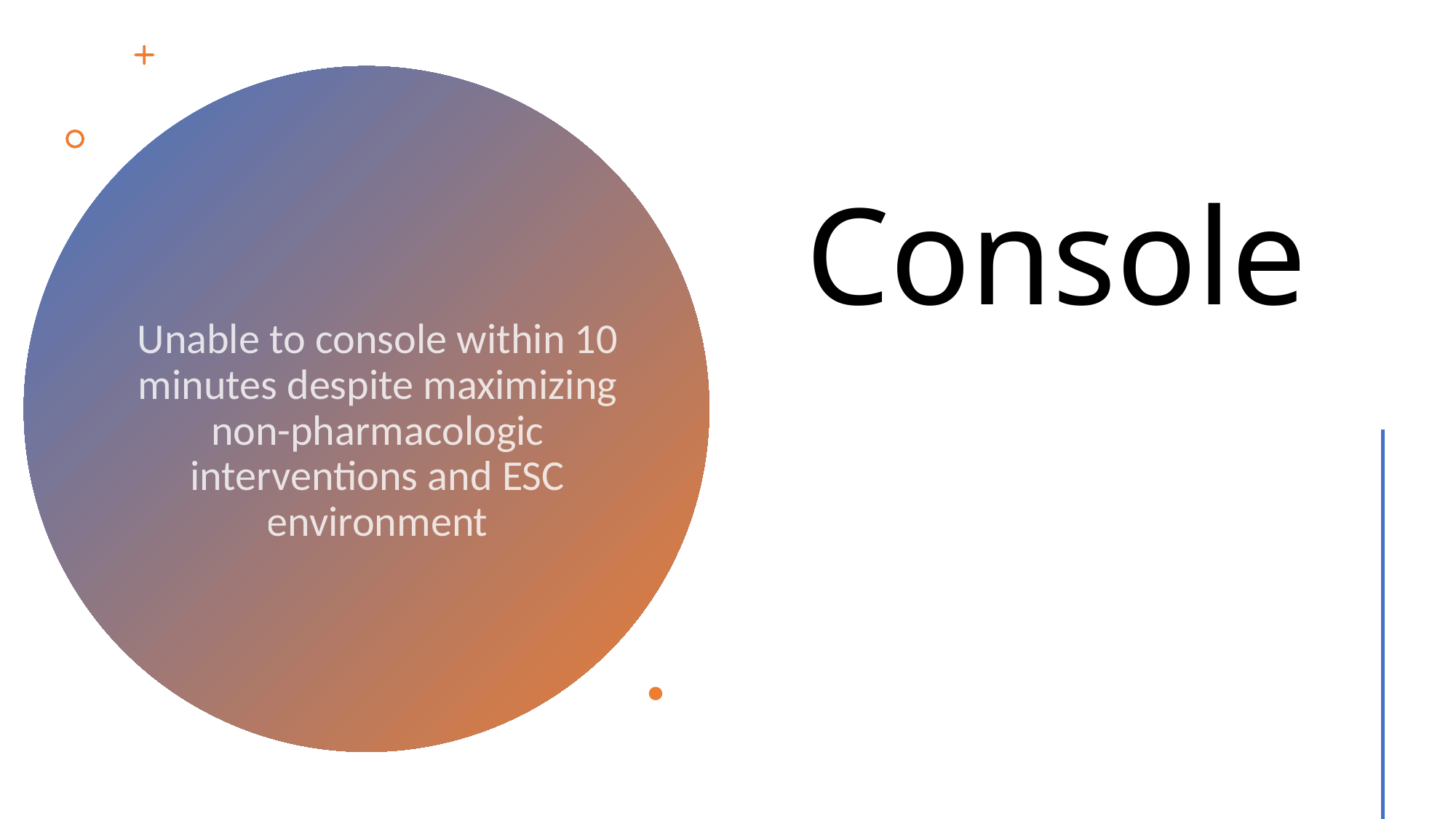

# Console
Unable to console within 10 minutes despite maximizing non-pharmacologic interventions and ESC environment

## Slide 25
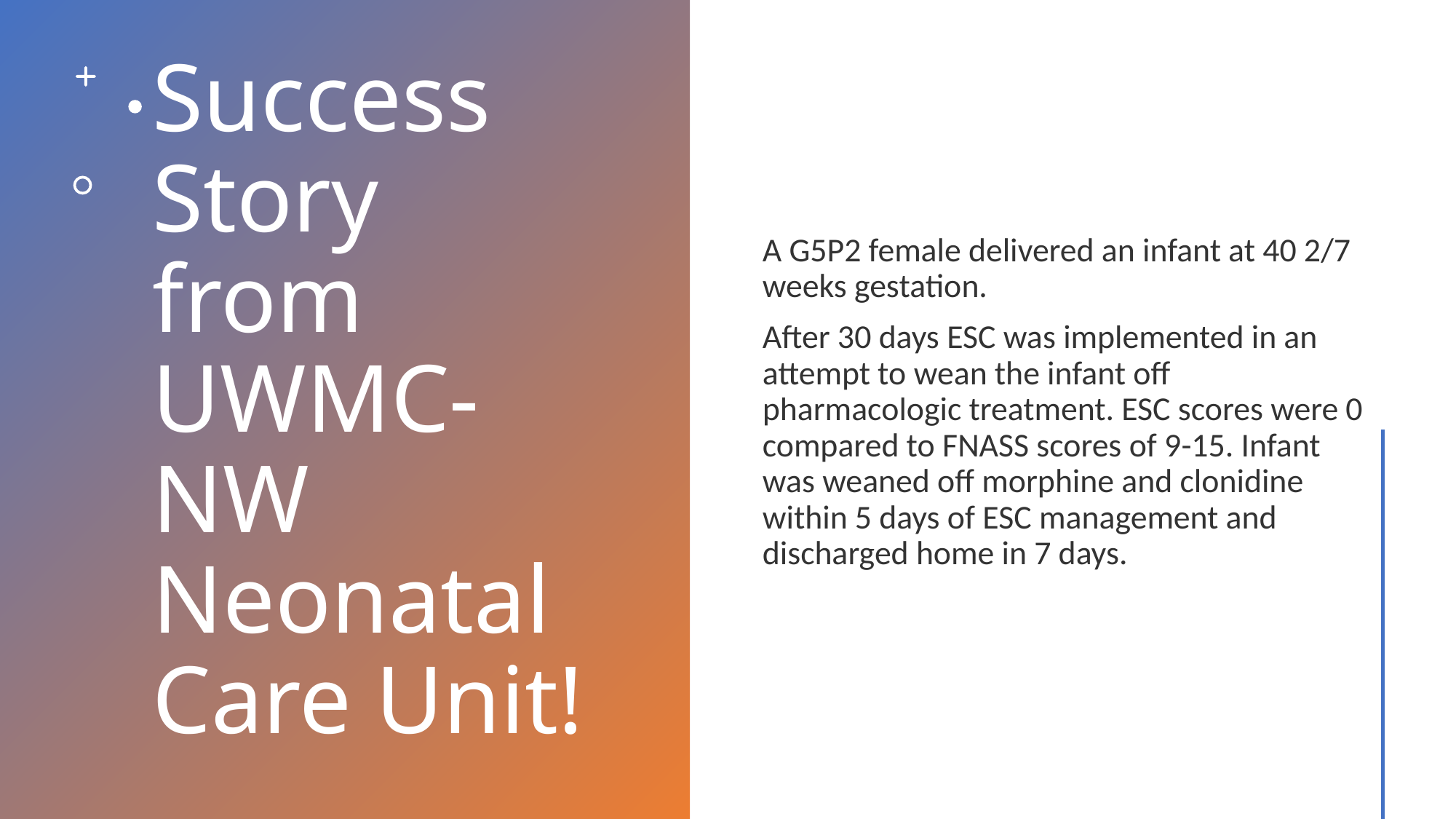

# Success Story from UWMC-NW Neonatal Care Unit!
A G5P2 female delivered an infant at 40 2/7 weeks gestation.
After 30 days ESC was implemented in an attempt to wean the infant off pharmacologic treatment. ESC scores were 0 compared to FNASS scores of 9-15. Infant was weaned off morphine and clonidine within 5 days of ESC management and discharged home in 7 days.

## Slide 26
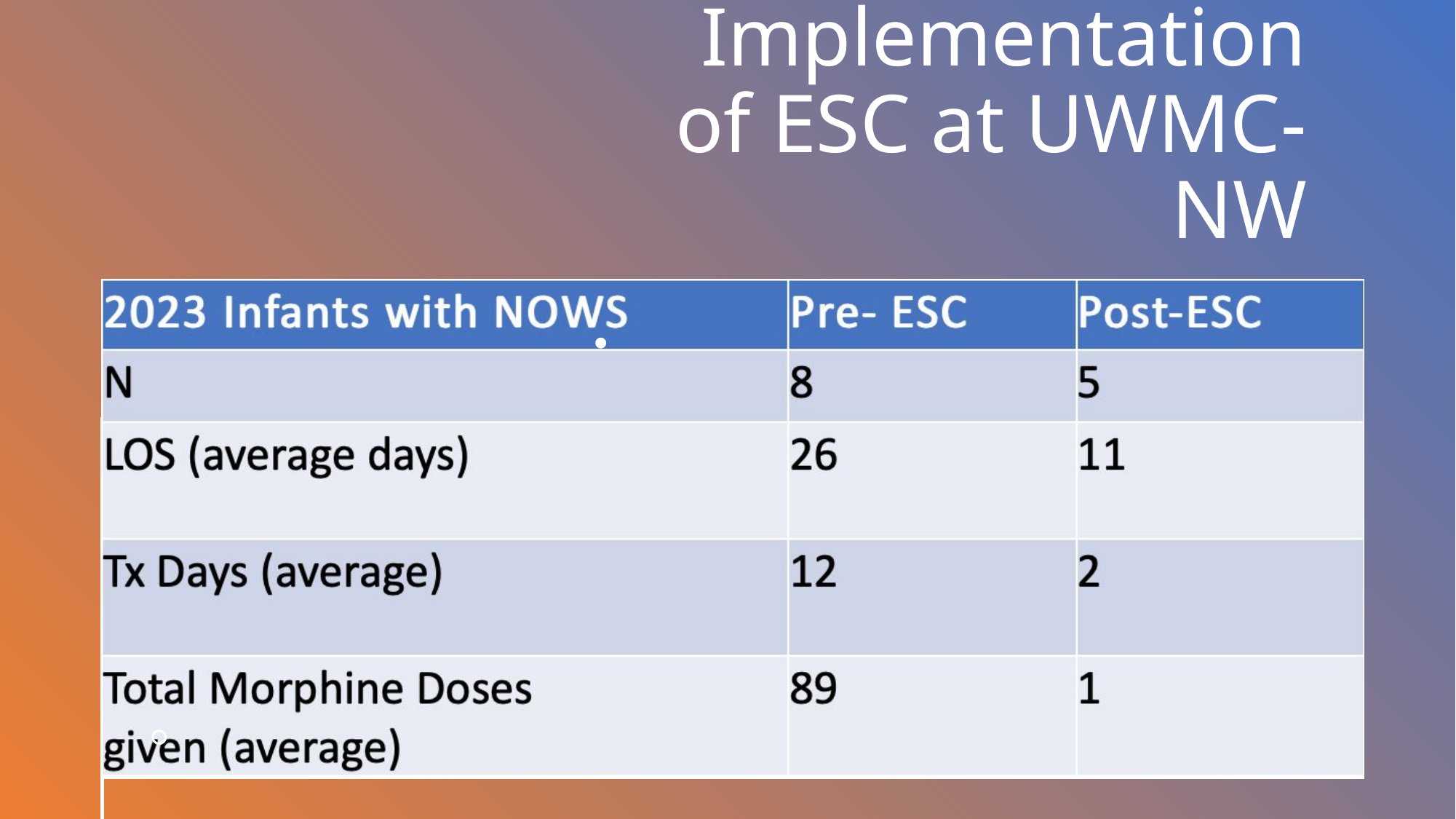

# Implementation of ESC at UWMC-NW

## Slide 27
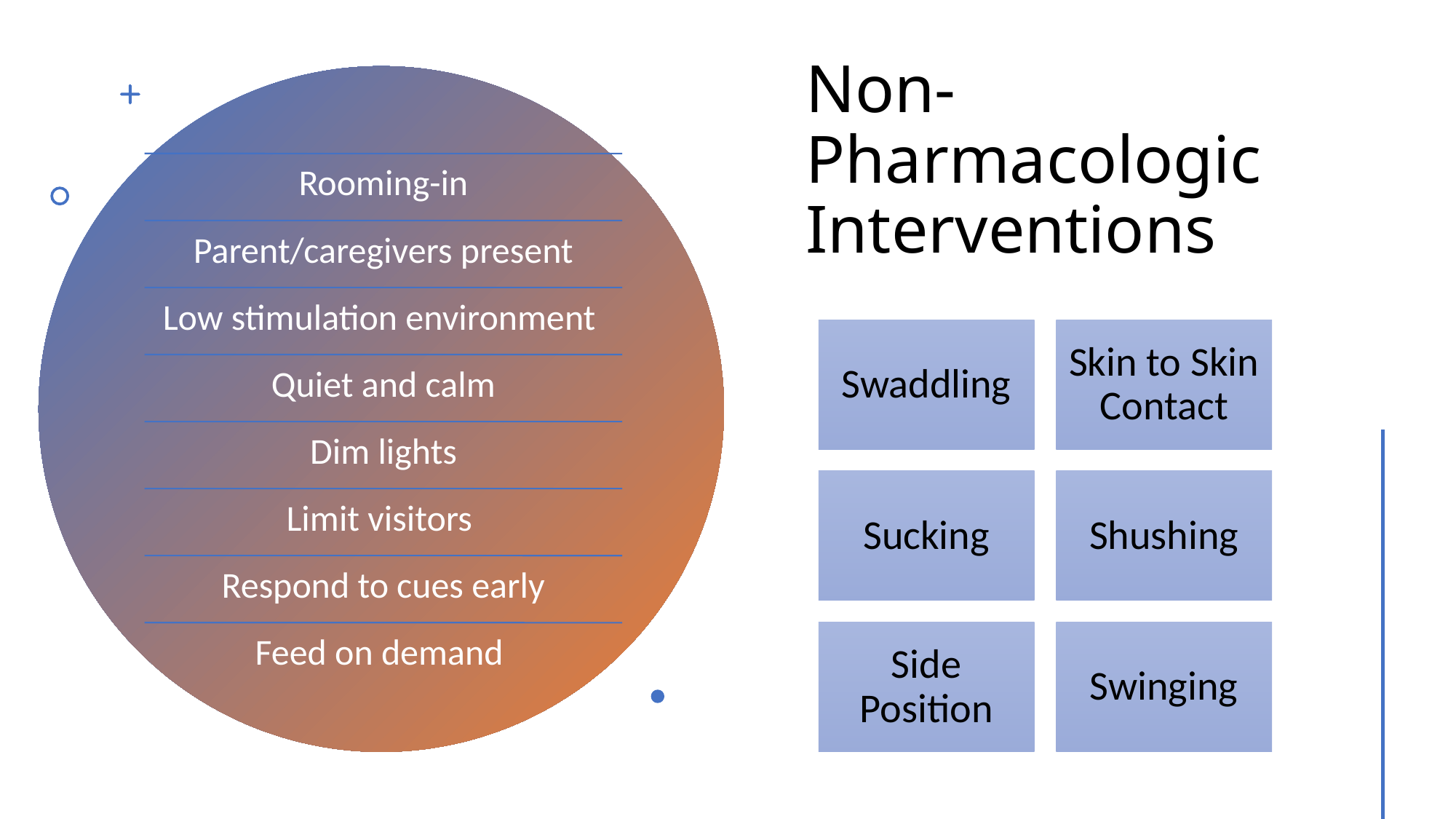

# Non-Pharmacologic Interventions

## Slide 28
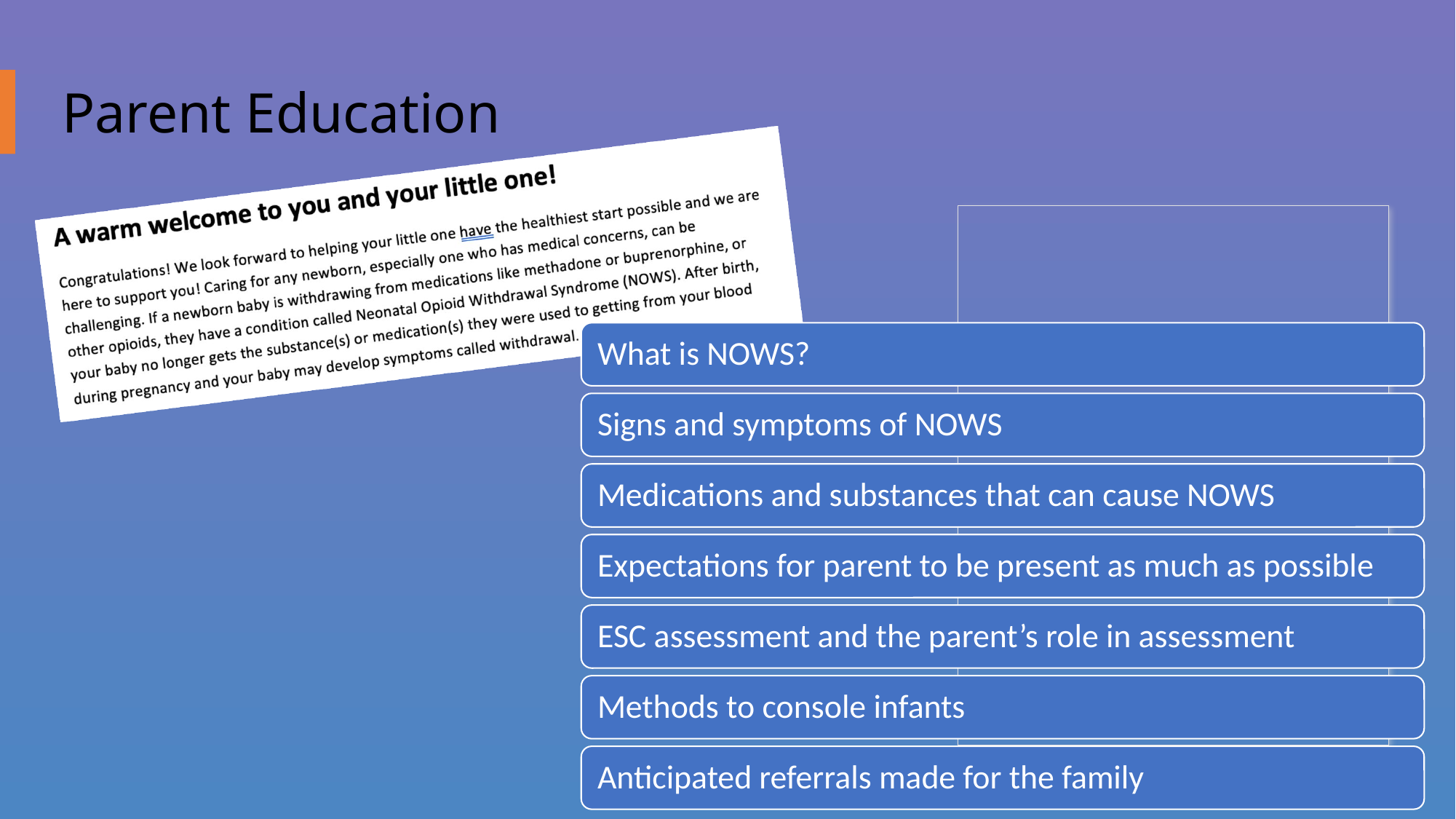

# Parent Education

## Slide 29
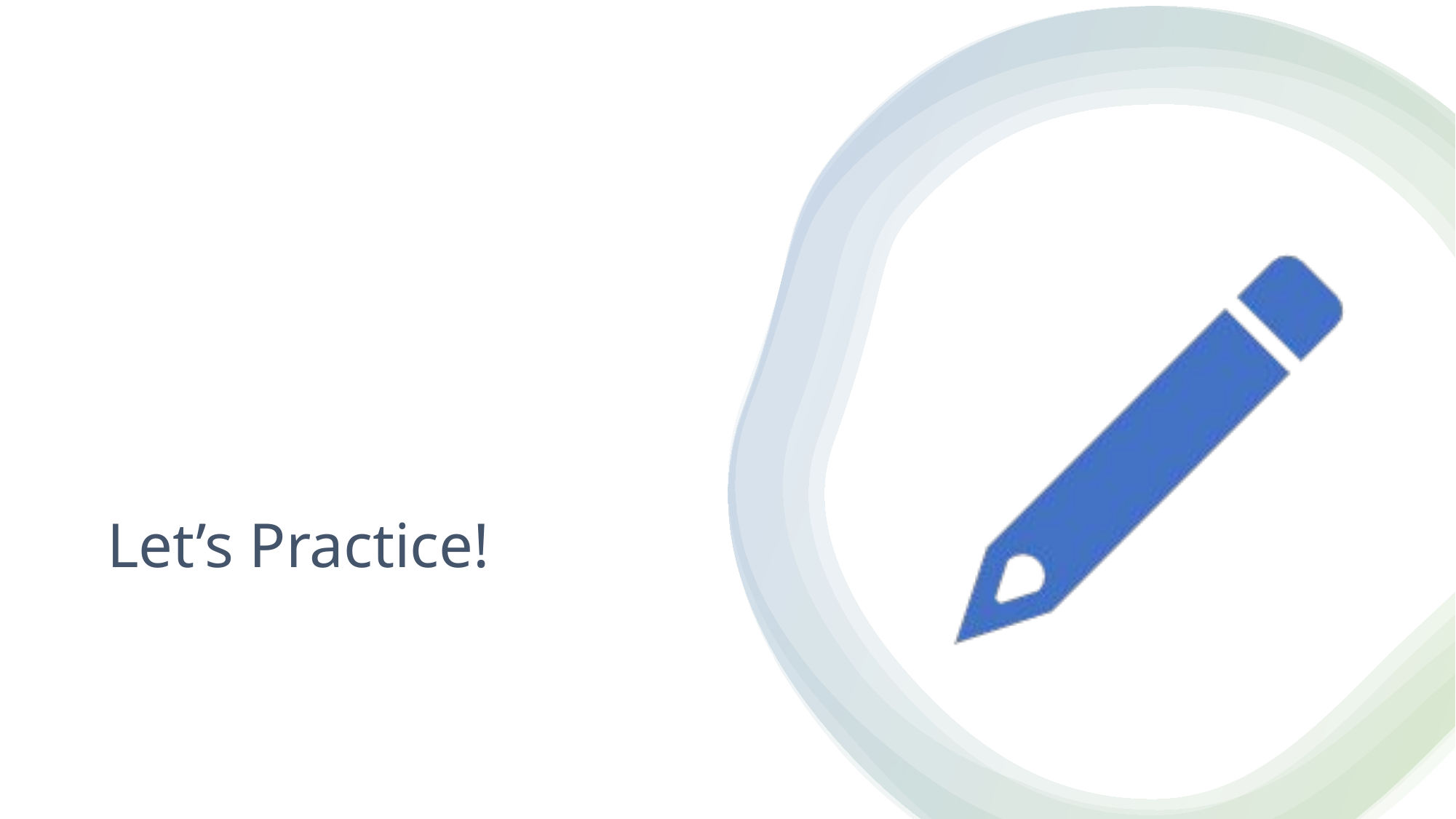

# Let’s Practice!

## Slide 30
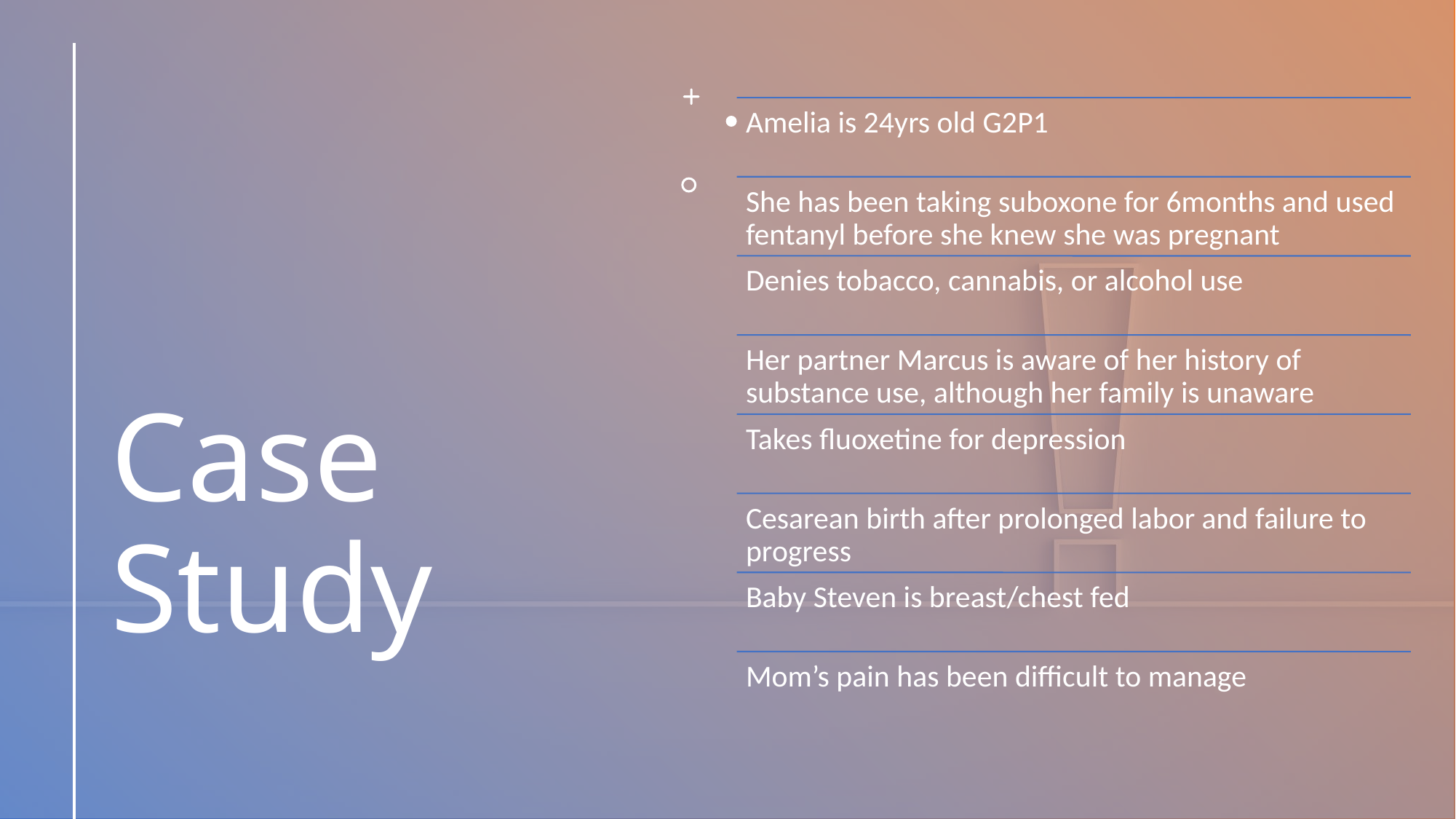

# Case Study

## Slide 31
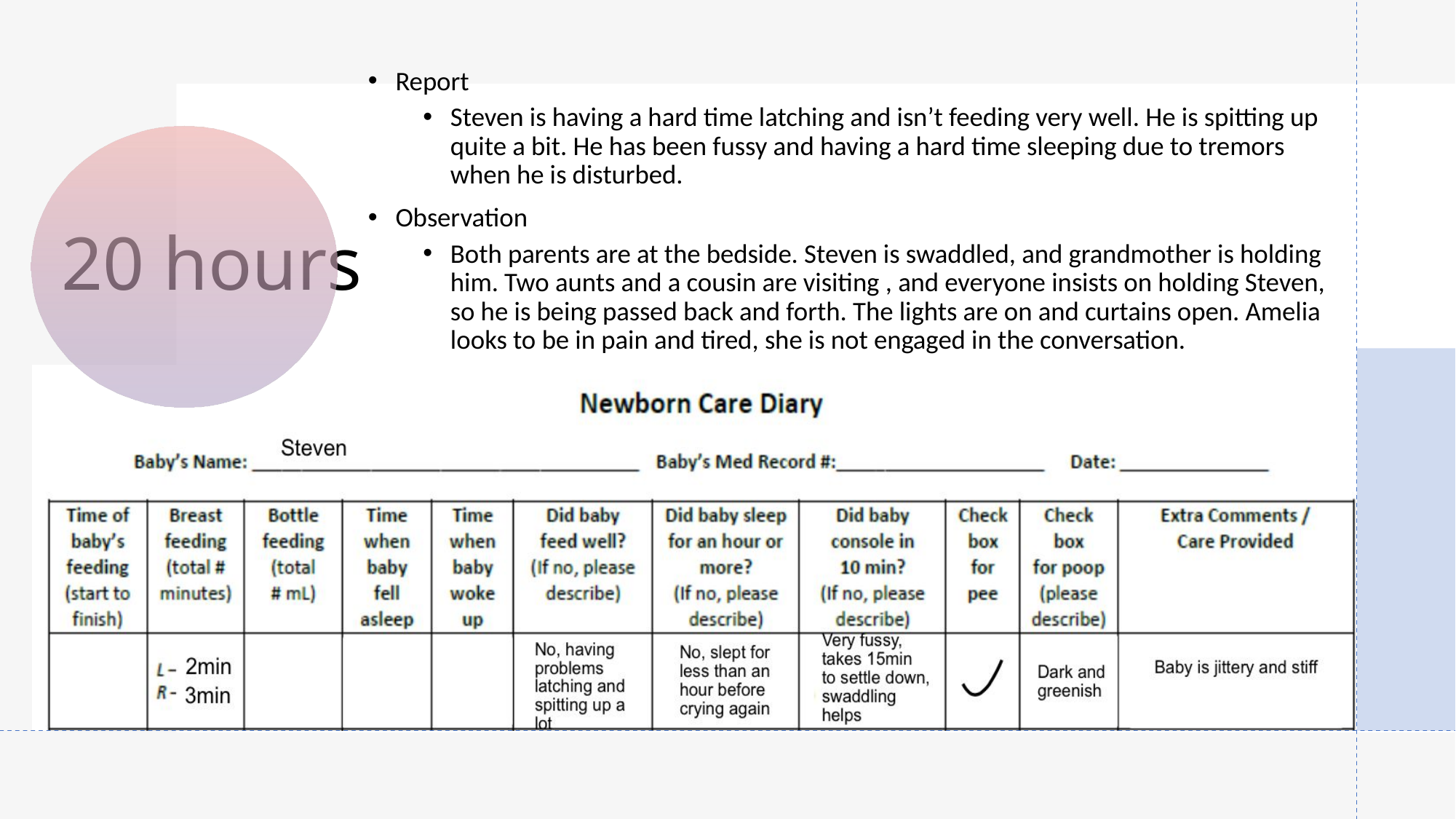

Report
Steven is having a hard time latching and isn’t feeding very well. He is spitting up quite a bit. He has been fussy and having a hard time sleeping due to tremors when he is disturbed.
Observation
Both parents are at the bedside. Steven is swaddled, and grandmother is holding him. Two aunts and a cousin are visiting , and everyone insists on holding Steven, so he is being passed back and forth. The lights are on and curtains open. Amelia looks to be in pain and tired, she is not engaged in the conversation.
# 20 hours

## Slide 32
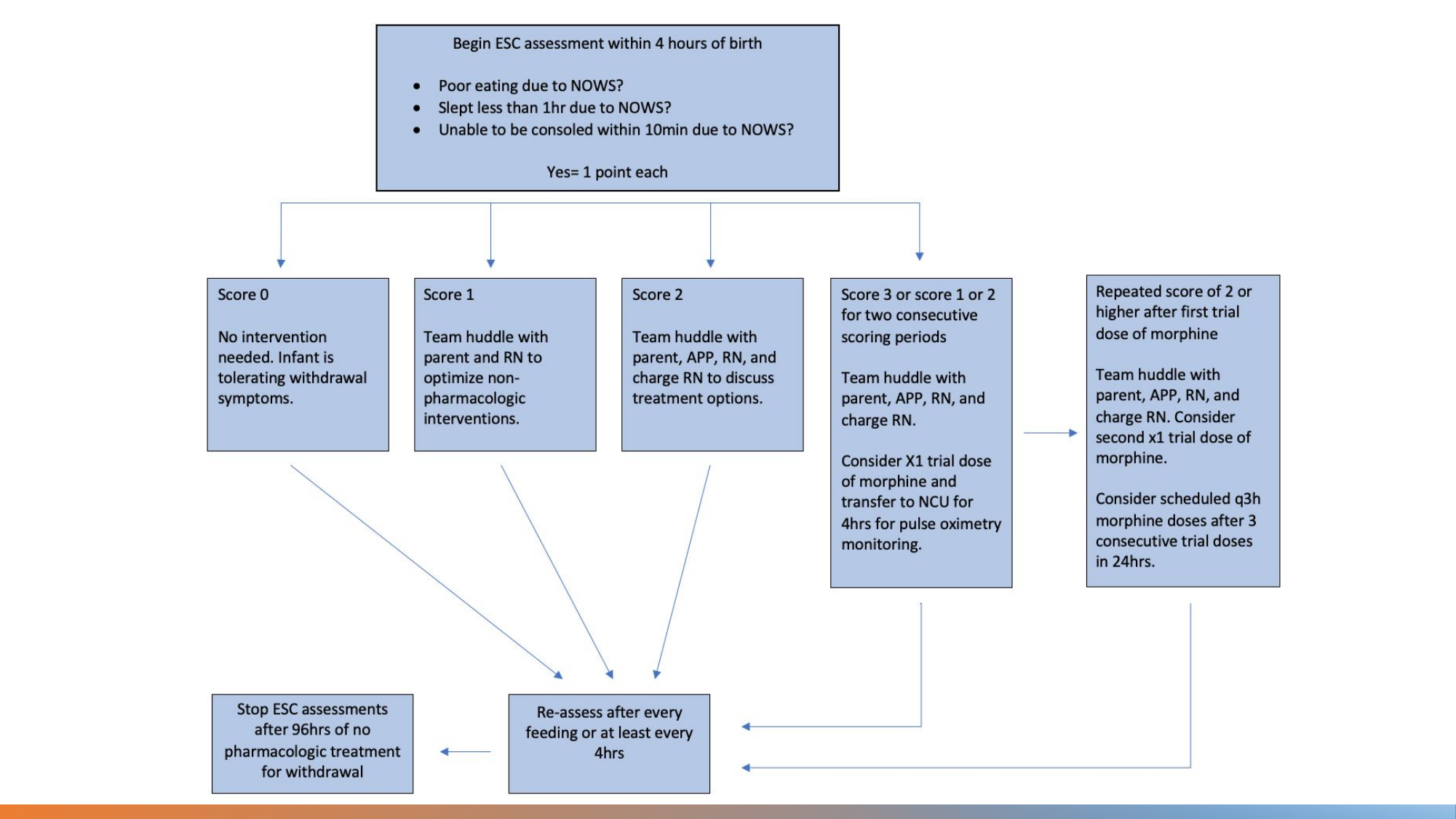

## Slide 33
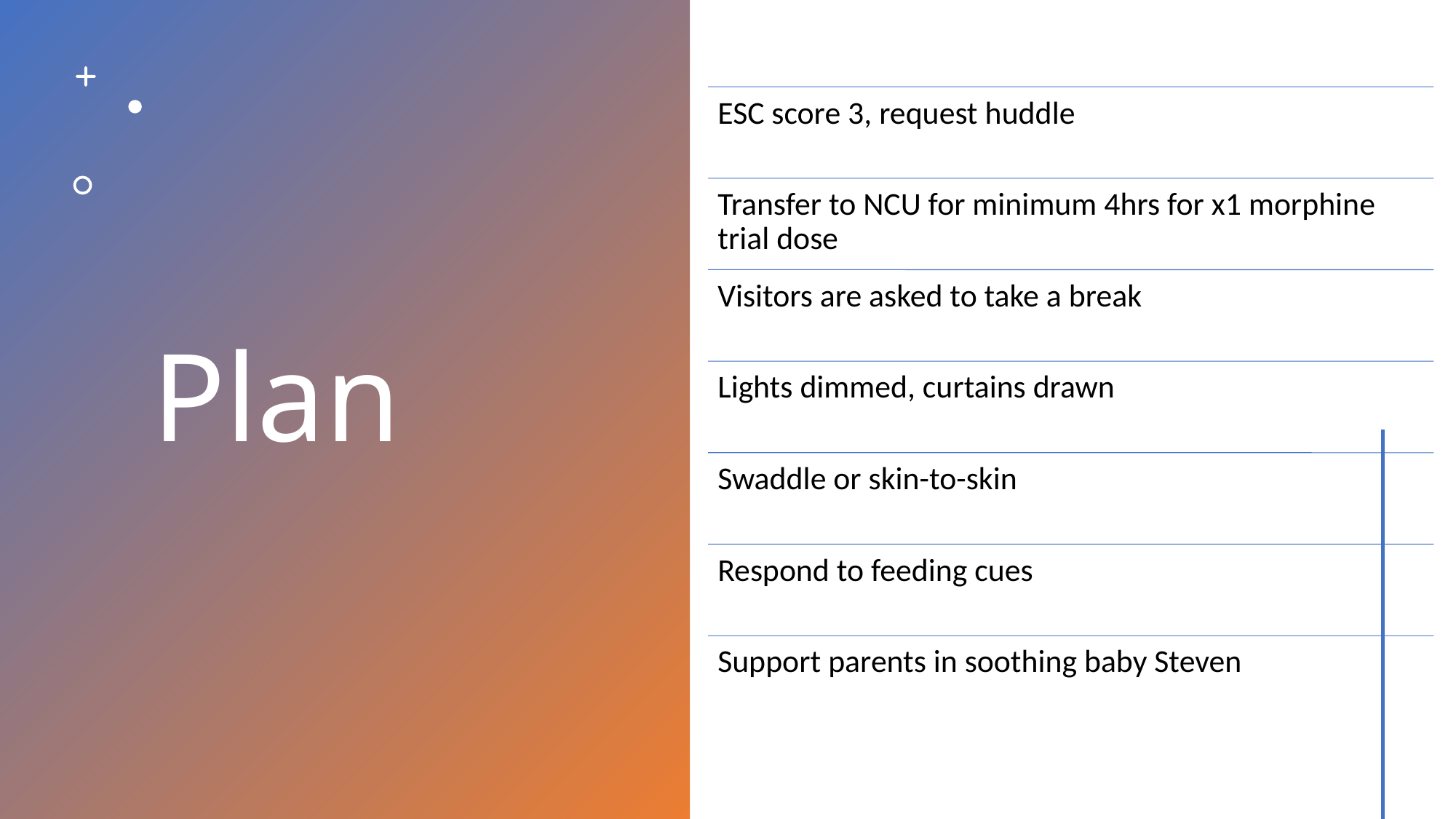

# Plan

## Slide 34
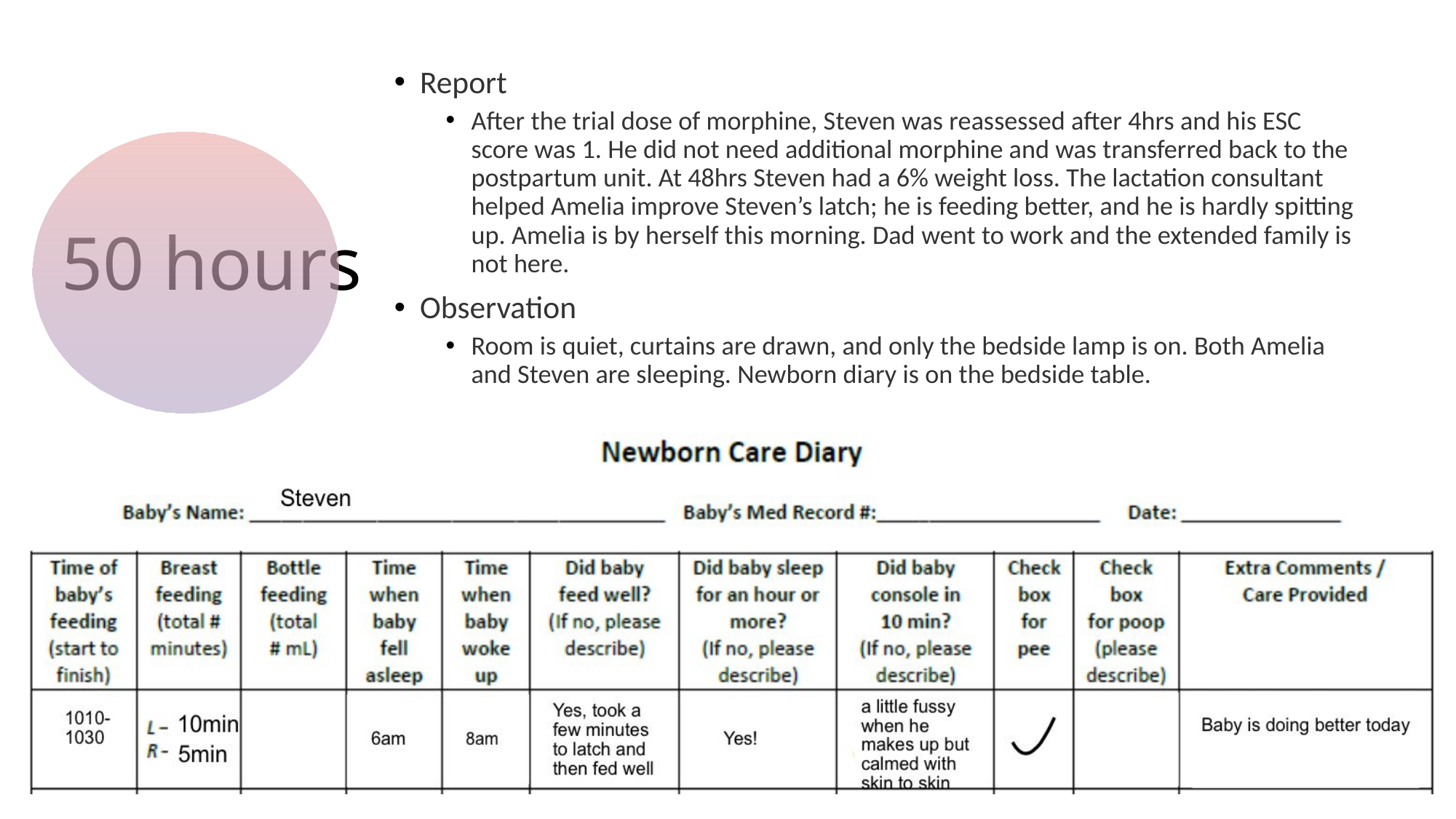

Report
After the trial dose of morphine, Steven was reassessed after 4hrs and his ESC score was 1. He did not need additional morphine and was transferred back to the postpartum unit. At 48hrs Steven had a 6% weight loss. The lactation consultant helped Amelia improve Steven’s latch; he is feeding better, and he is hardly spitting up. Amelia is by herself this morning. Dad went to work and the extended family is not here.
Observation
Room is quiet, curtains are drawn, and only the bedside lamp is on. Both Amelia and Steven are sleeping. Newborn diary is on the bedside table.
# 50 hours

## Slide 35
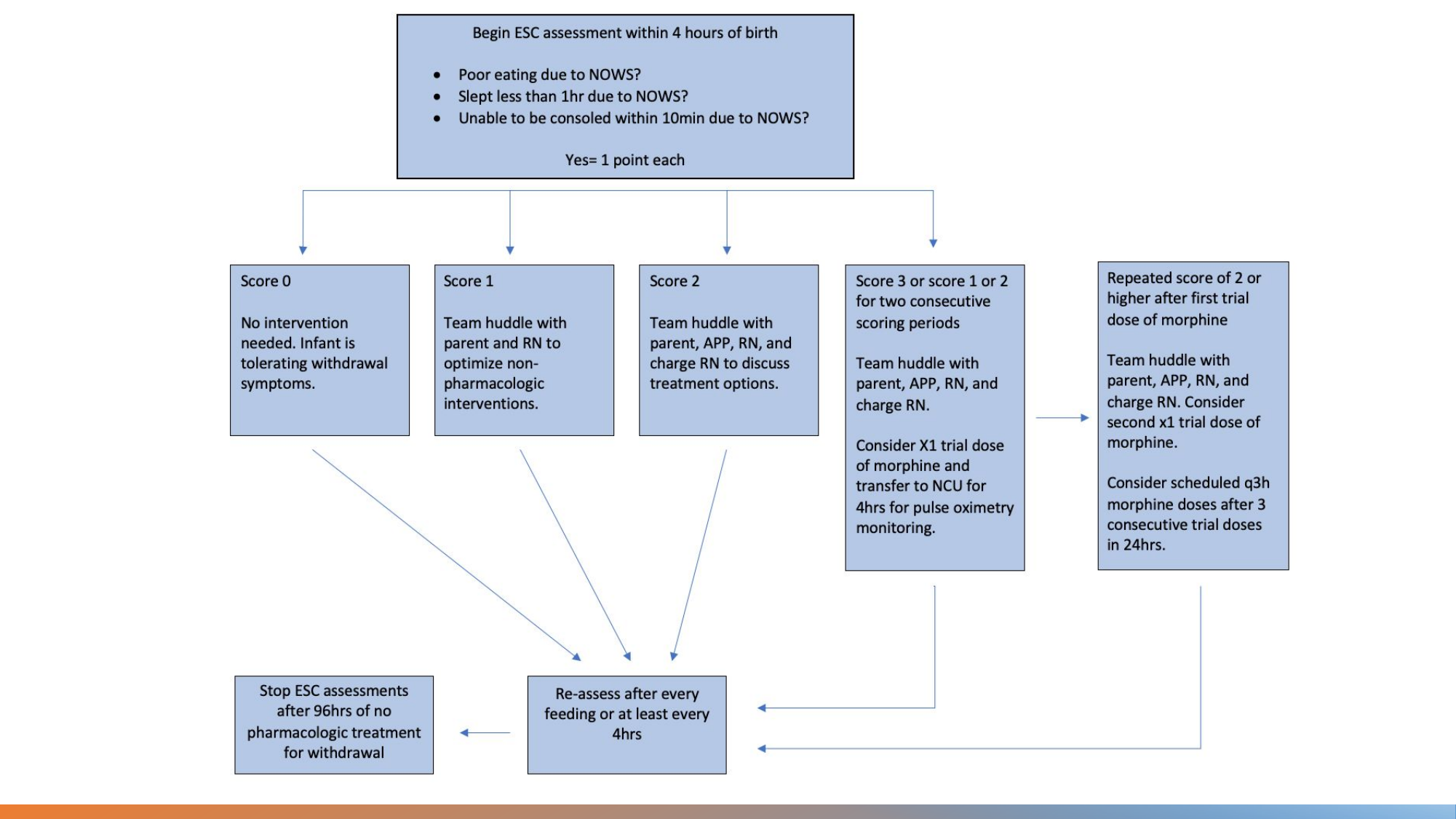

## Slide 36
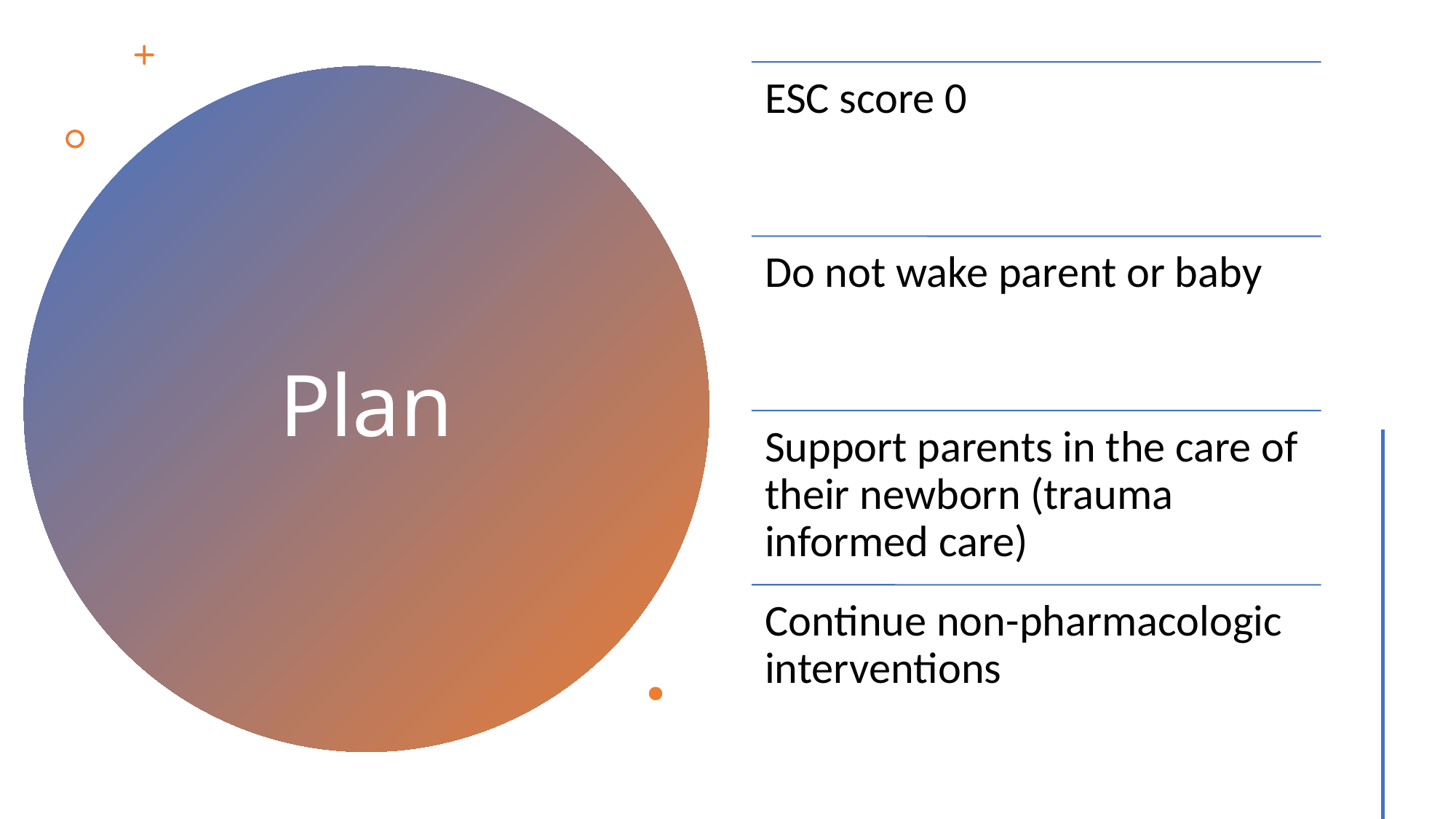

# Plan

## Slide 37
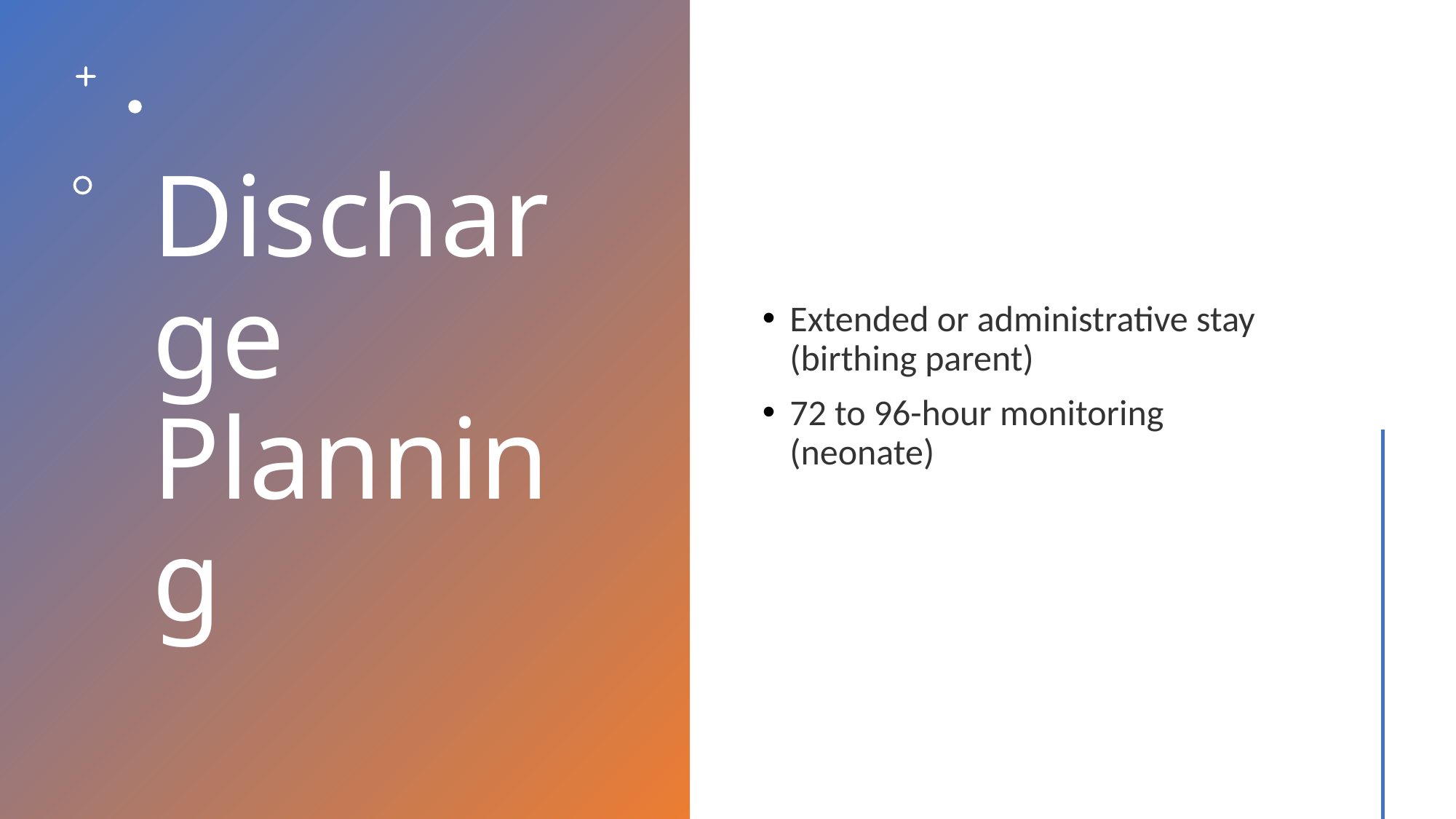

# Discharge Planning
Extended or administrative stay (birthing parent)
72 to 96-hour monitoring (neonate)

## Slide 38
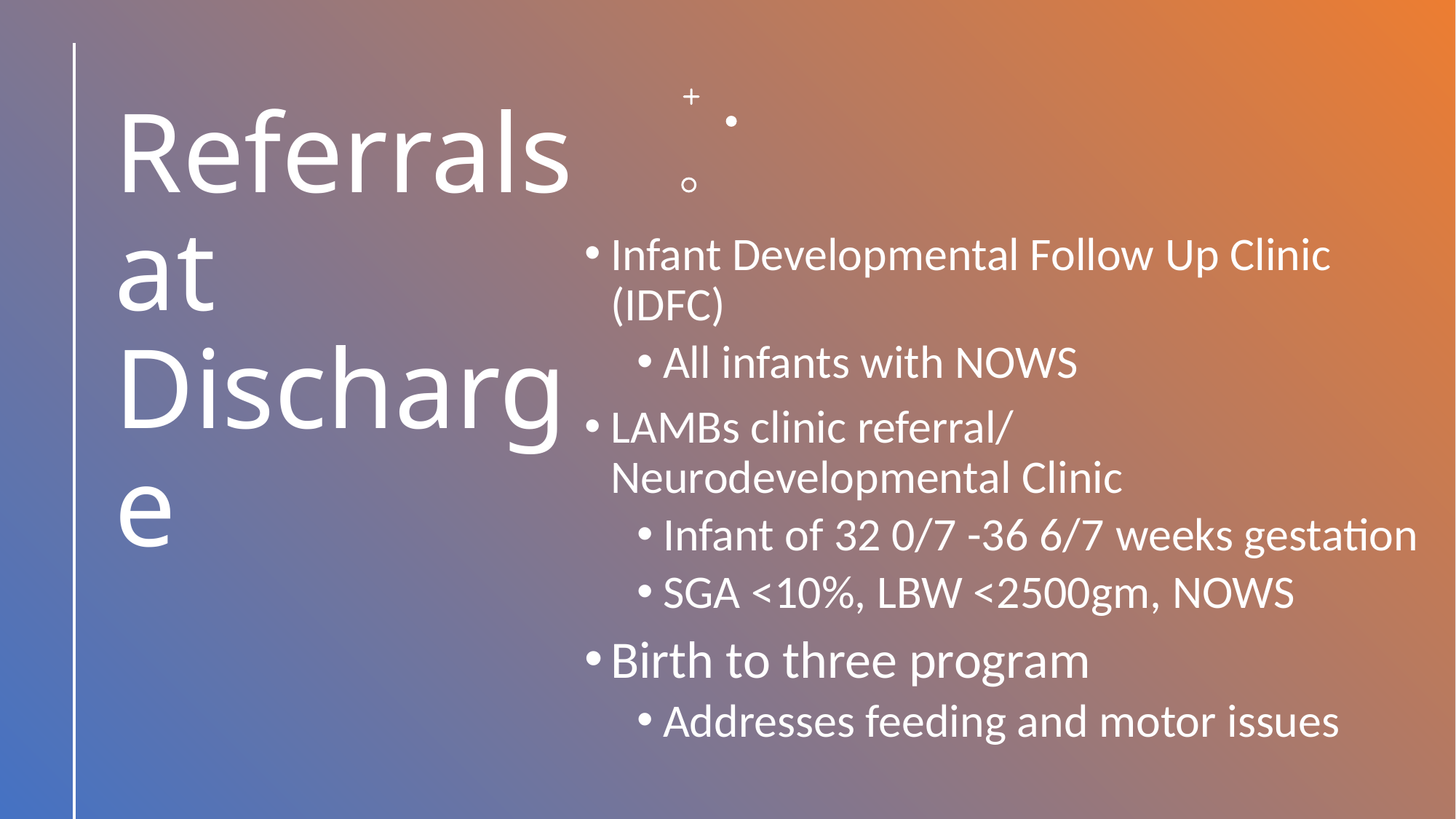

# Referrals at Discharge
Infant Developmental Follow Up Clinic (IDFC)
All infants with NOWS
LAMBs clinic referral/ Neurodevelopmental Clinic
Infant of 32 0/7 -36 6/7 weeks gestation
SGA <10%, LBW <2500gm, NOWS
Birth to three program
Addresses feeding and motor issues

## Slide 39
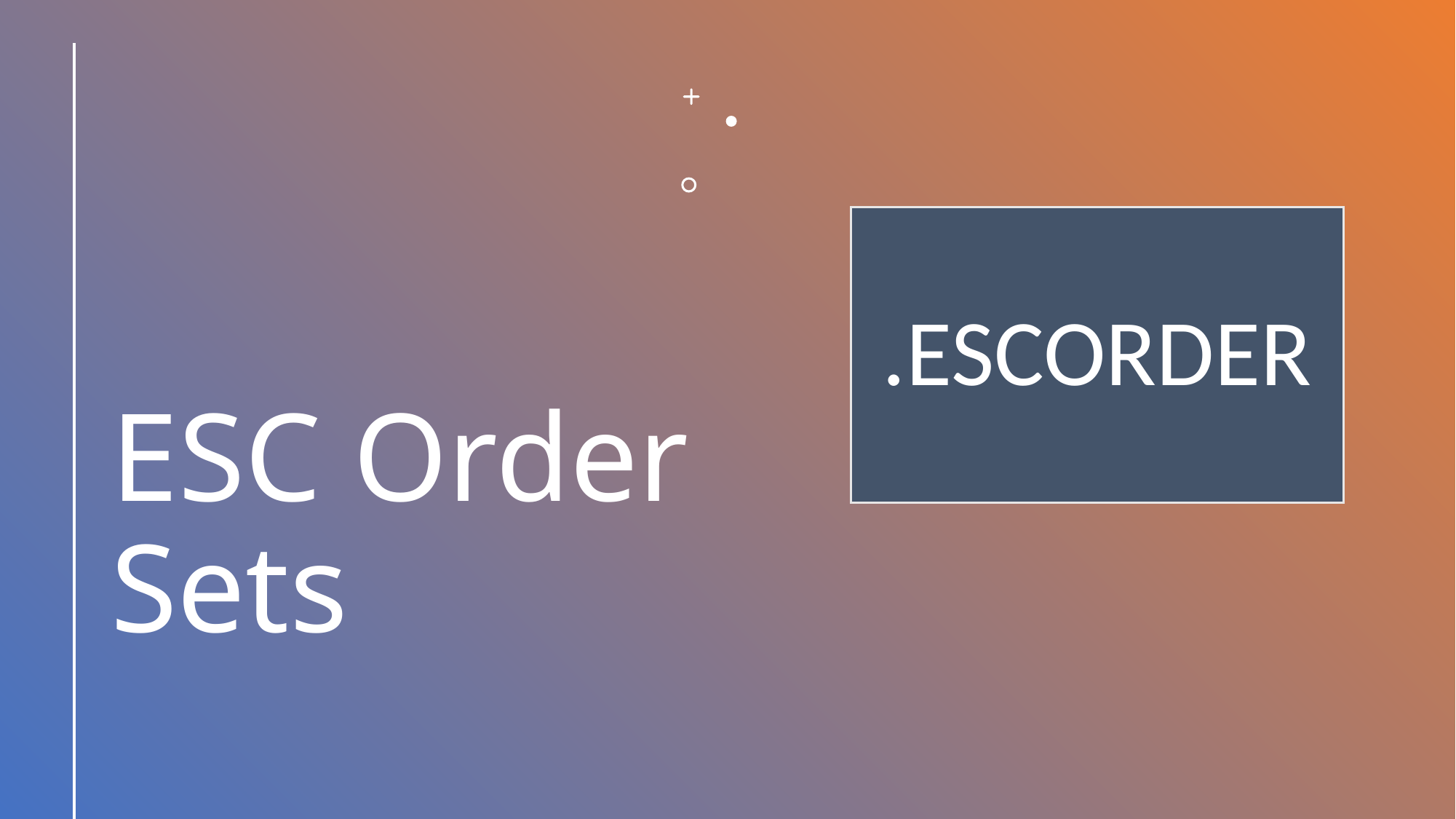

# ESC Order Sets

## Slide 40
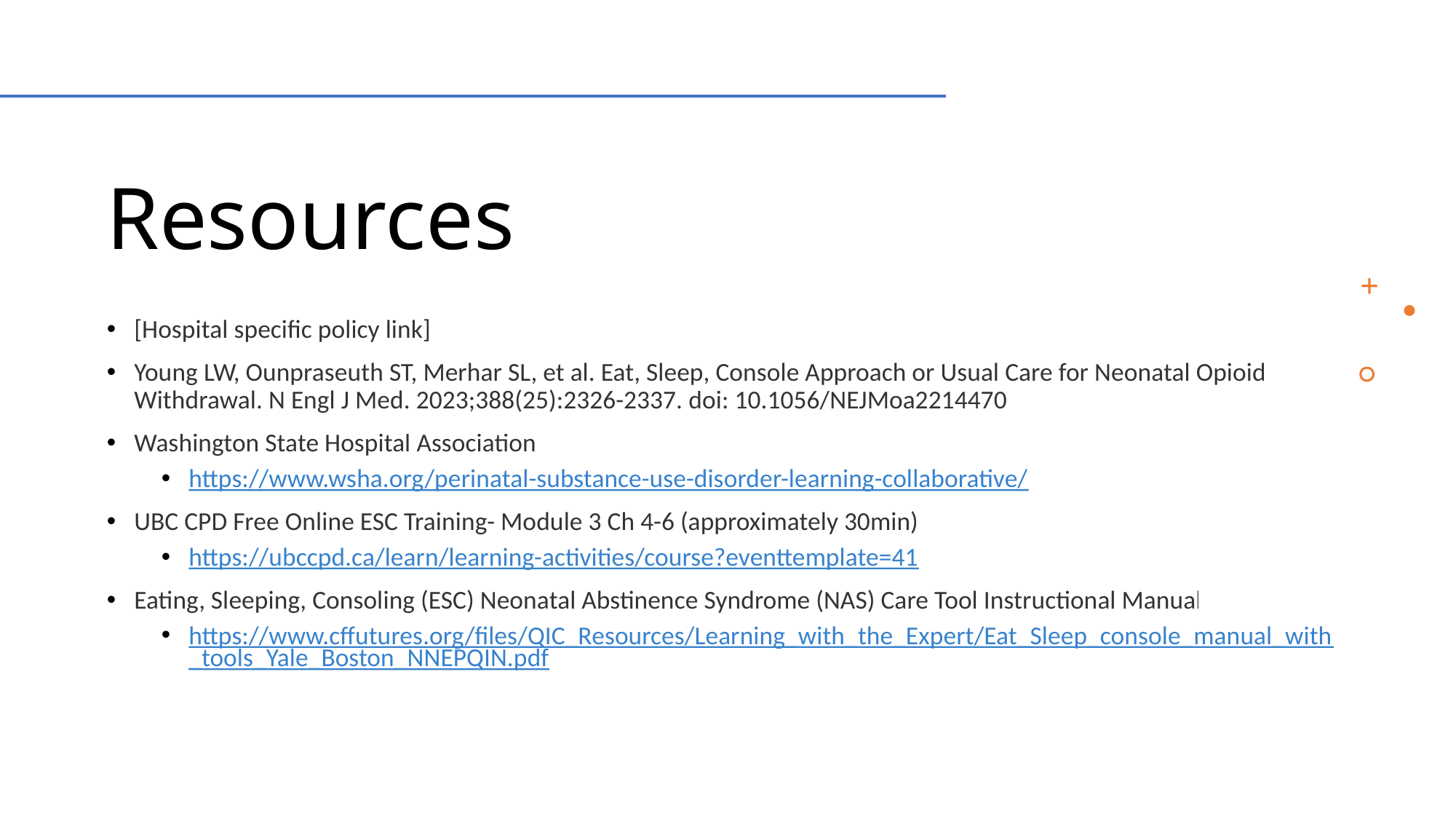

# Resources
[Hospital specific policy link]
Young LW, Ounpraseuth ST, Merhar SL, et al. Eat, Sleep, Console Approach or Usual Care for Neonatal Opioid Withdrawal. N Engl J Med. 2023;388(25):2326-2337. doi: 10.1056/NEJMoa2214470
Washington State Hospital Association
https://www.wsha.org/perinatal-substance-use-disorder-learning-collaborative/
UBC CPD Free Online ESC Training- Module 3 Ch 4-6 (approximately 30min)
https://ubccpd.ca/learn/learning-activities/course?eventtemplate=41
Eating, Sleeping, Consoling (ESC) Neonatal Abstinence Syndrome (NAS) Care Tool Instructional Manual
https://www.cffutures.org/files/QIC_Resources/Learning_with_the_Expert/Eat_Sleep_console_manual_with_tools_Yale_Boston_NNEPQIN.pdf

## Slide 41
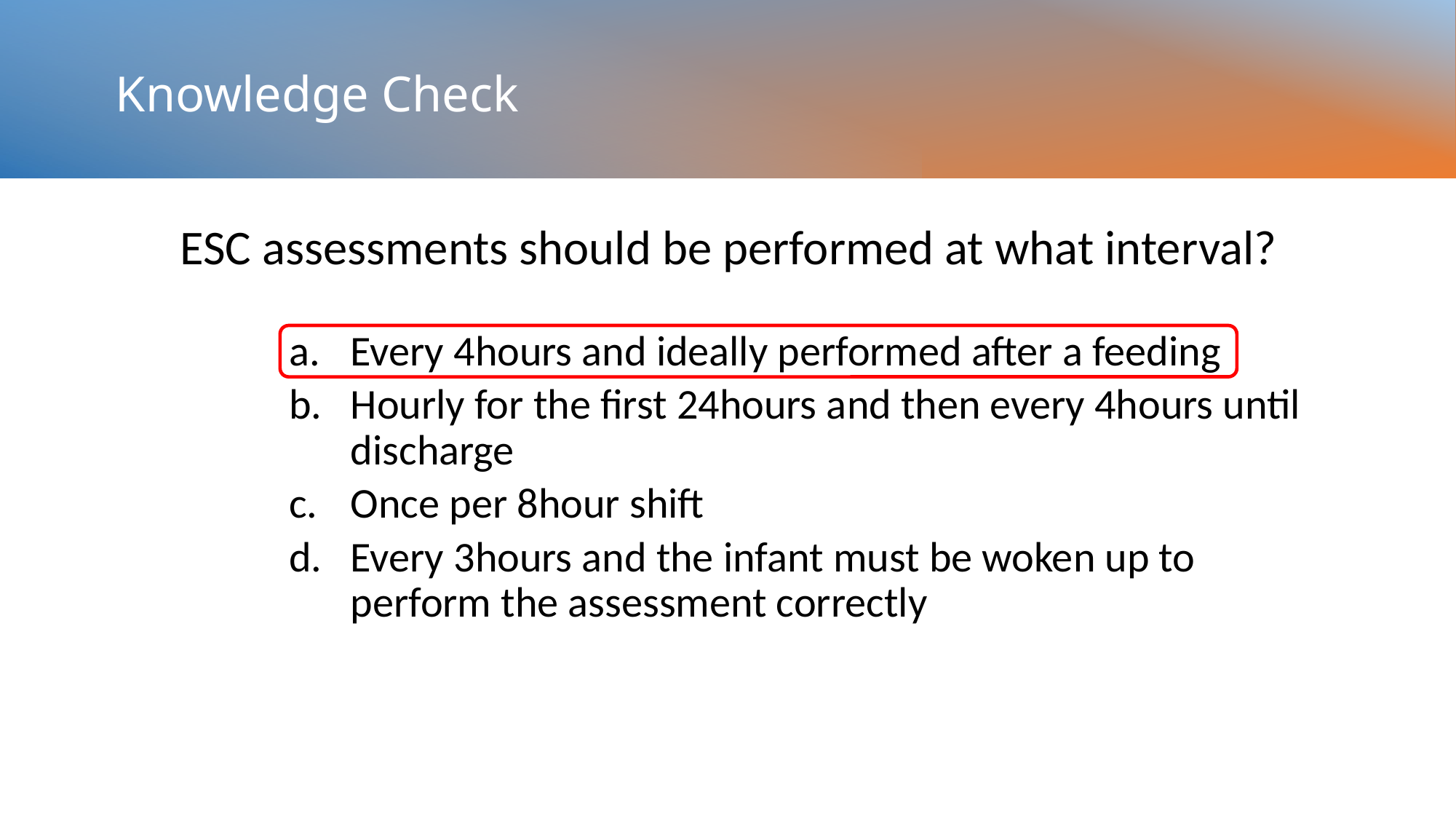

# Knowledge Check
ESC assessments should be performed at what interval?
Every 4hours and ideally performed after a feeding
Hourly for the first 24hours and then every 4hours until discharge
Once per 8hour shift
Every 3hours and the infant must be woken up to perform the assessment correctly

## Slide 42
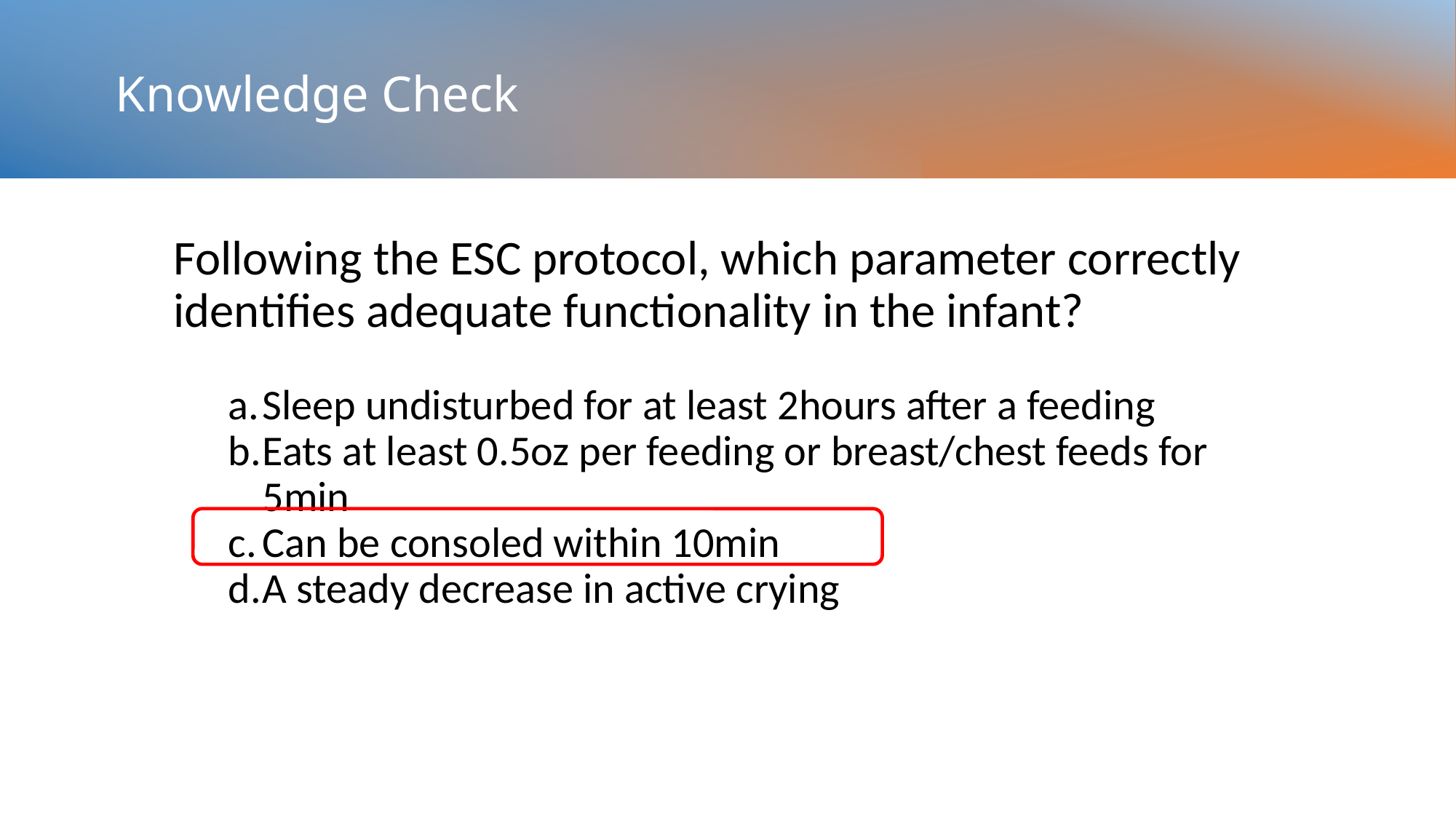

# Knowledge Check
Following the ESC protocol, which parameter correctly identifies adequate functionality in the infant?
Sleep undisturbed for at least 2hours after a feeding
Eats at least 0.5oz per feeding or breast/chest feeds for 5min
Can be consoled within 10min
A steady decrease in active crying

## Slide 43
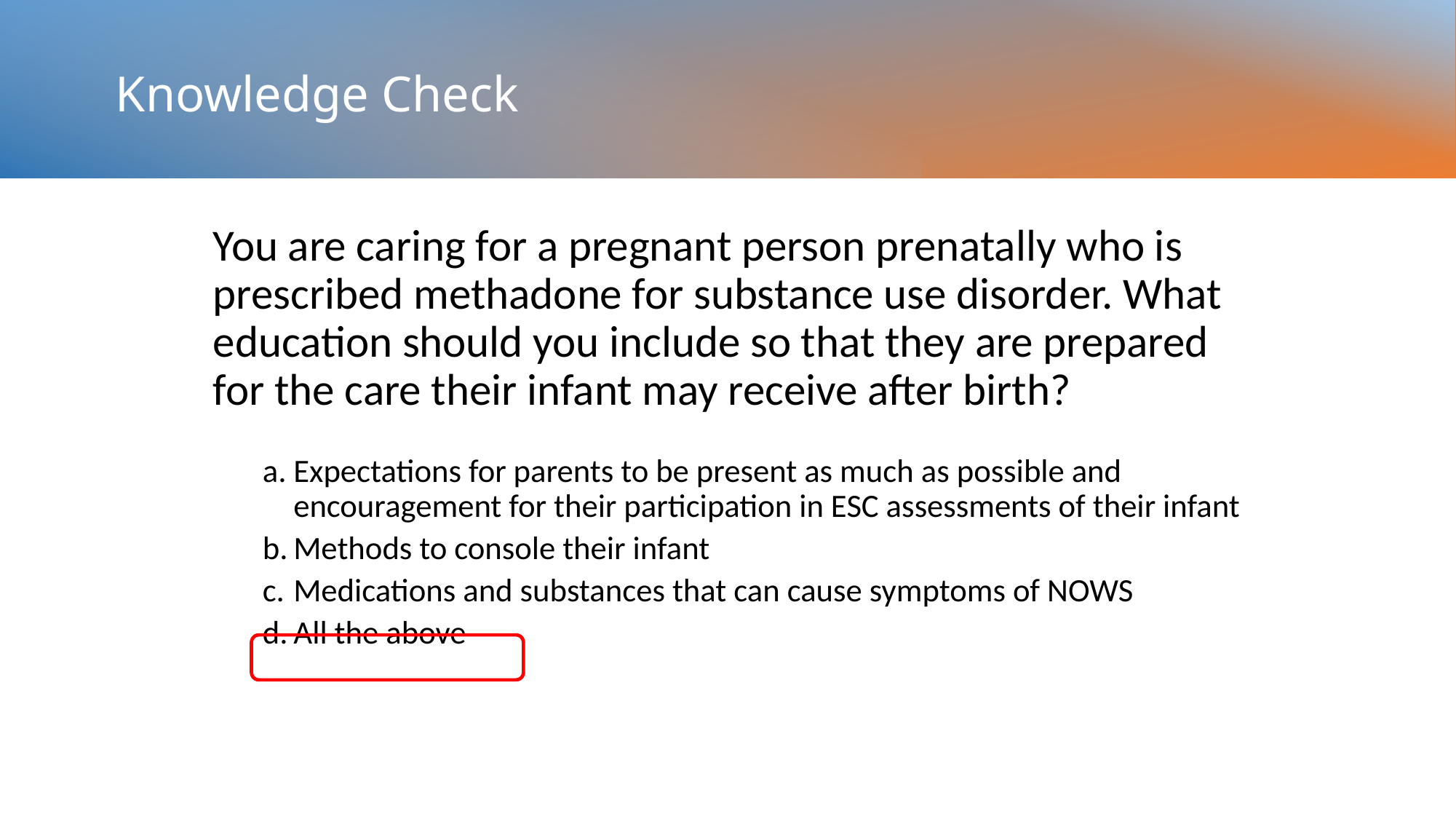

# Knowledge Check
You are caring for a pregnant person prenatally who is prescribed methadone for substance use disorder. What education should you include so that they are prepared for the care their infant may receive after birth?
Expectations for parents to be present as much as possible and encouragement for their participation in ESC assessments of their infant
Methods to console their infant
Medications and substances that can cause symptoms of NOWS
All the above

## Slide 44
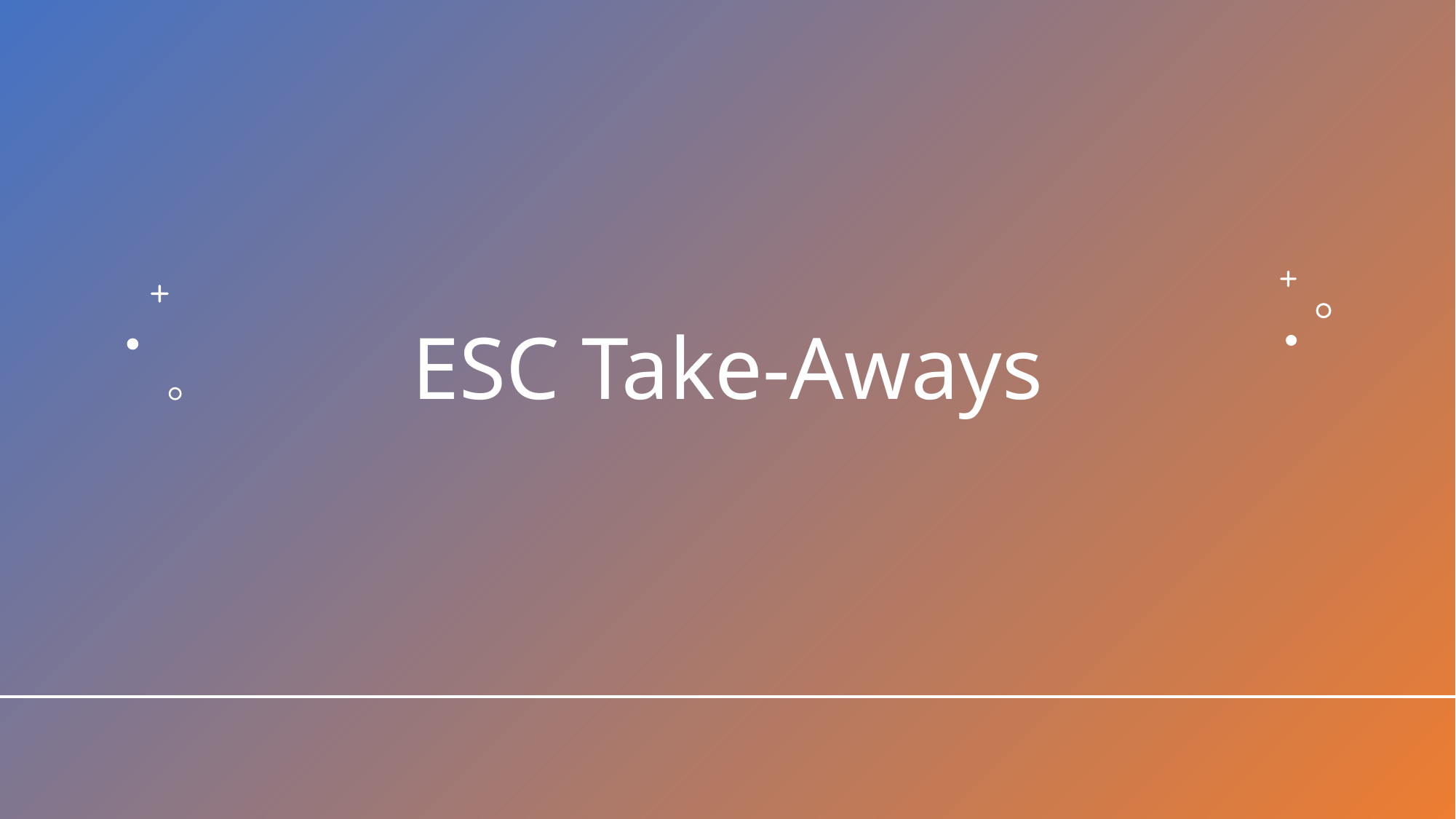

# ESC Take-Aways

## Slide 45
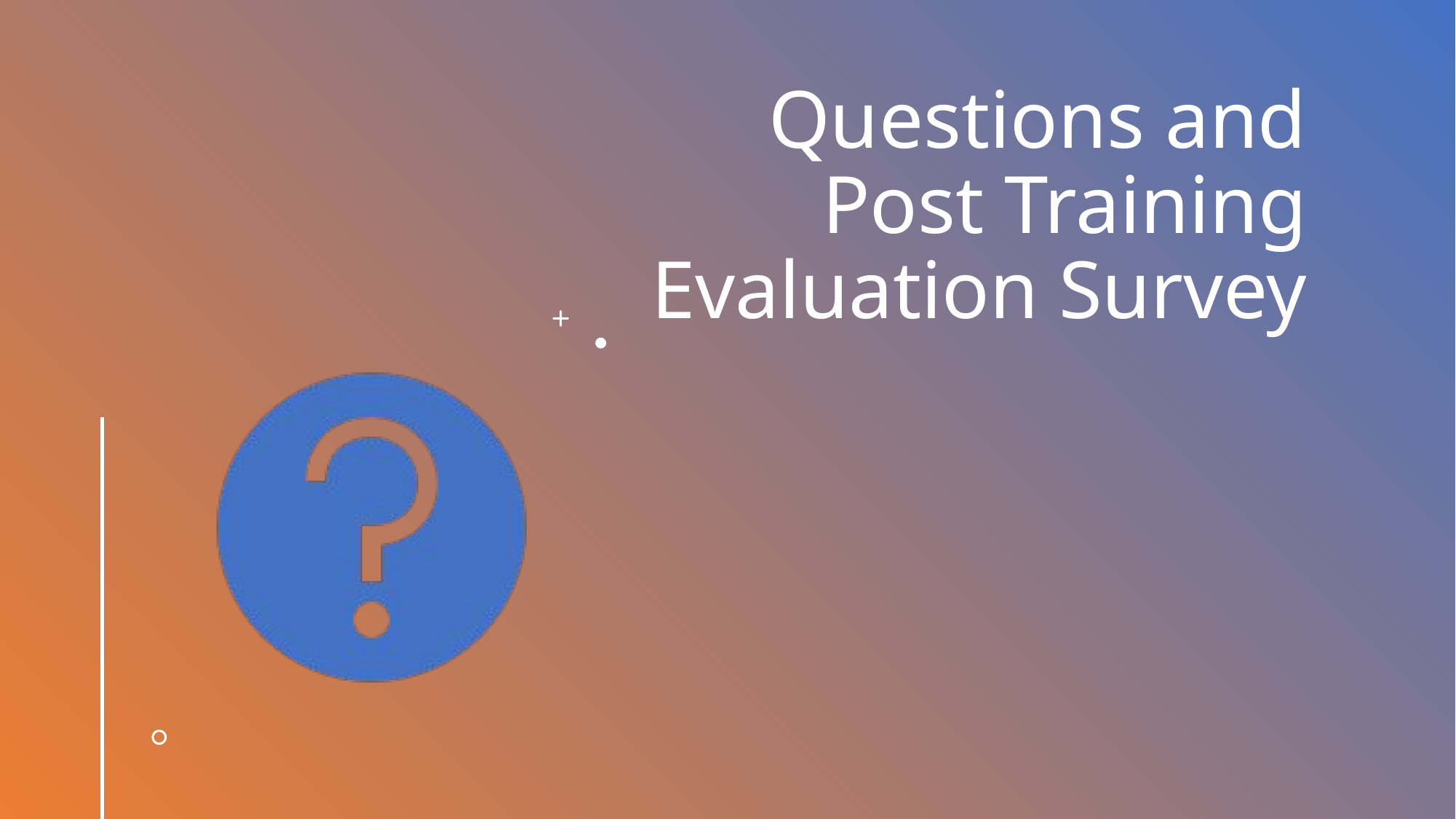

# Questions and Post Training Evaluation Survey

## Slide 46
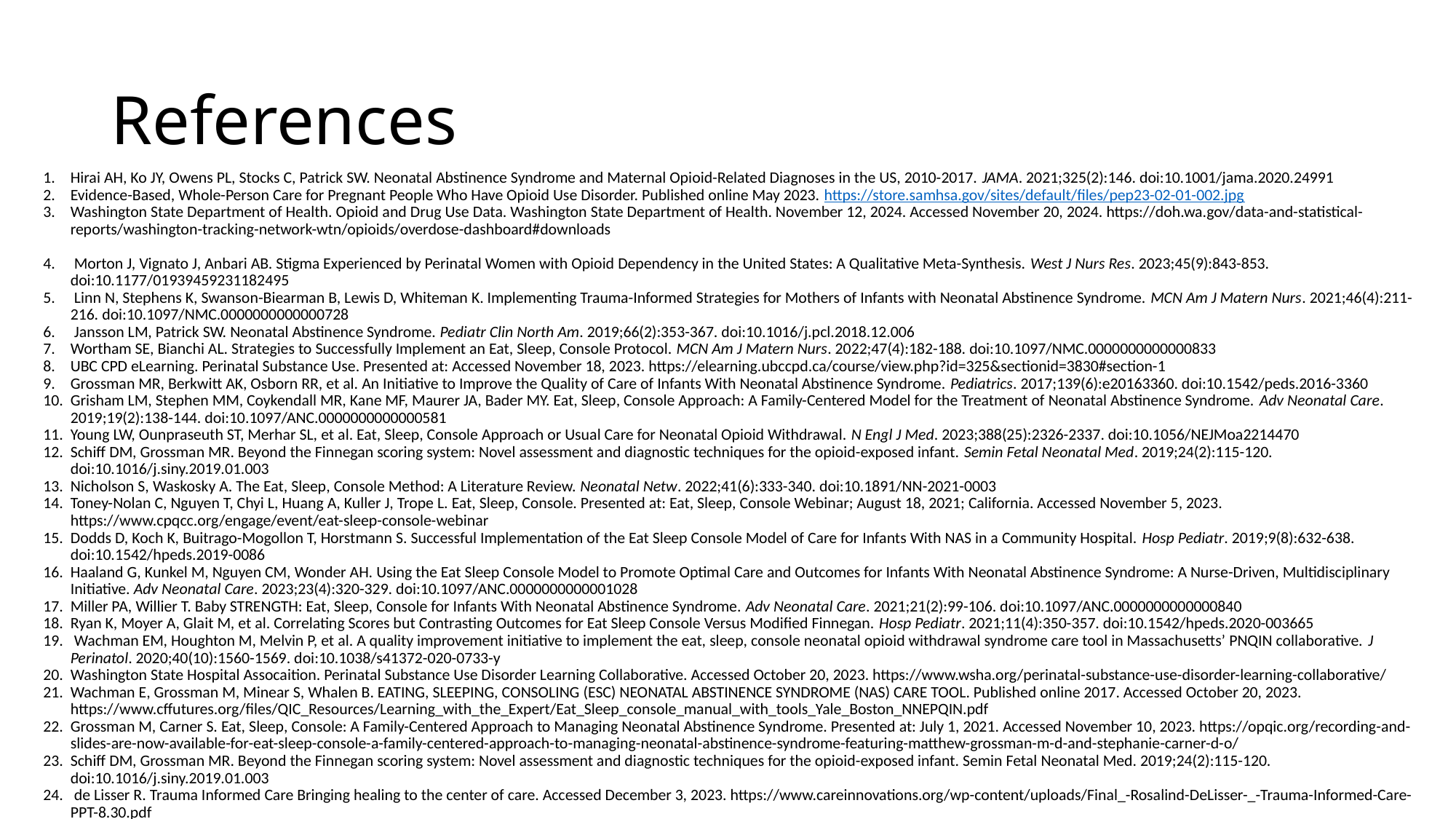

# References
Hirai AH, Ko JY, Owens PL, Stocks C, Patrick SW. Neonatal Abstinence Syndrome and Maternal Opioid-Related Diagnoses in the US, 2010-2017. JAMA. 2021;325(2):146. doi:10.1001/jama.2020.24991
Evidence-Based, Whole-Person Care for Pregnant People Who Have Opioid Use Disorder. Published online May 2023. https://store.samhsa.gov/sites/default/files/pep23-02-01-002.jpg
Washington State Department of Health. Opioid and Drug Use Data. Washington State Department of Health. November 12, 2024. Accessed November 20, 2024. https://doh.wa.gov/data-and-statistical-reports/washington-tracking-network-wtn/opioids/overdose-dashboard#downloads
 Morton J, Vignato J, Anbari AB. Stigma Experienced by Perinatal Women with Opioid Dependency in the United States: A Qualitative Meta-Synthesis. West J Nurs Res. 2023;45(9):843-853. doi:10.1177/01939459231182495
 Linn N, Stephens K, Swanson-Biearman B, Lewis D, Whiteman K. Implementing Trauma-Informed Strategies for Mothers of Infants with Neonatal Abstinence Syndrome. MCN Am J Matern Nurs. 2021;46(4):211-216. doi:10.1097/NMC.0000000000000728
 Jansson LM, Patrick SW. Neonatal Abstinence Syndrome. Pediatr Clin North Am. 2019;66(2):353-367. doi:10.1016/j.pcl.2018.12.006
Wortham SE, Bianchi AL. Strategies to Successfully Implement an Eat, Sleep, Console Protocol. MCN Am J Matern Nurs. 2022;47(4):182-188. doi:10.1097/NMC.0000000000000833
UBC CPD eLearning. Perinatal Substance Use. Presented at: Accessed November 18, 2023. https://elearning.ubccpd.ca/course/view.php?id=325&sectionid=3830#section-1
Grossman MR, Berkwitt AK, Osborn RR, et al. An Initiative to Improve the Quality of Care of Infants With Neonatal Abstinence Syndrome. Pediatrics. 2017;139(6):e20163360. doi:10.1542/peds.2016-3360
Grisham LM, Stephen MM, Coykendall MR, Kane MF, Maurer JA, Bader MY. Eat, Sleep, Console Approach: A Family-Centered Model for the Treatment of Neonatal Abstinence Syndrome. Adv Neonatal Care. 2019;19(2):138-144. doi:10.1097/ANC.0000000000000581
Young LW, Ounpraseuth ST, Merhar SL, et al. Eat, Sleep, Console Approach or Usual Care for Neonatal Opioid Withdrawal. N Engl J Med. 2023;388(25):2326-2337. doi:10.1056/NEJMoa2214470
Schiff DM, Grossman MR. Beyond the Finnegan scoring system: Novel assessment and diagnostic techniques for the opioid-exposed infant. Semin Fetal Neonatal Med. 2019;24(2):115-120. doi:10.1016/j.siny.2019.01.003
Nicholson S, Waskosky A. The Eat, Sleep, Console Method: A Literature Review. Neonatal Netw. 2022;41(6):333-340. doi:10.1891/NN-2021-0003
Toney-Nolan C, Nguyen T, Chyi L, Huang A, Kuller J, Trope L. Eat, Sleep, Console. Presented at: Eat, Sleep, Console Webinar; August 18, 2021; California. Accessed November 5, 2023. https://www.cpqcc.org/engage/event/eat-sleep-console-webinar
Dodds D, Koch K, Buitrago-Mogollon T, Horstmann S. Successful Implementation of the Eat Sleep Console Model of Care for Infants With NAS in a Community Hospital. Hosp Pediatr. 2019;9(8):632-638. doi:10.1542/hpeds.2019-0086
Haaland G, Kunkel M, Nguyen CM, Wonder AH. Using the Eat Sleep Console Model to Promote Optimal Care and Outcomes for Infants With Neonatal Abstinence Syndrome: A Nurse-Driven, Multidisciplinary Initiative. Adv Neonatal Care. 2023;23(4):320-329. doi:10.1097/ANC.0000000000001028
Miller PA, Willier T. Baby STRENGTH: Eat, Sleep, Console for Infants With Neonatal Abstinence Syndrome. Adv Neonatal Care. 2021;21(2):99-106. doi:10.1097/ANC.0000000000000840
Ryan K, Moyer A, Glait M, et al. Correlating Scores but Contrasting Outcomes for Eat Sleep Console Versus Modified Finnegan. Hosp Pediatr. 2021;11(4):350-357. doi:10.1542/hpeds.2020-003665
 Wachman EM, Houghton M, Melvin P, et al. A quality improvement initiative to implement the eat, sleep, console neonatal opioid withdrawal syndrome care tool in Massachusetts’ PNQIN collaborative. J Perinatol. 2020;40(10):1560-1569. doi:10.1038/s41372-020-0733-y
Washington State Hospital Assocaition. Perinatal Substance Use Disorder Learning Collaborative. Accessed October 20, 2023. https://www.wsha.org/perinatal-substance-use-disorder-learning-collaborative/
Wachman E, Grossman M, Minear S, Whalen B. EATING, SLEEPING, CONSOLING (ESC) NEONATAL ABSTINENCE SYNDROME (NAS) CARE TOOL. Published online 2017. Accessed October 20, 2023. https://www.cffutures.org/files/QIC_Resources/Learning_with_the_Expert/Eat_Sleep_console_manual_with_tools_Yale_Boston_NNEPQIN.pdf
Grossman M, Carner S. Eat, Sleep, Console: A Family-Centered Approach to Managing Neonatal Abstinence Syndrome. Presented at: July 1, 2021. Accessed November 10, 2023. https://opqic.org/recording-and-slides-are-now-available-for-eat-sleep-console-a-family-centered-approach-to-managing-neonatal-abstinence-syndrome-featuring-matthew-grossman-m-d-and-stephanie-carner-d-o/
Schiff DM, Grossman MR. Beyond the Finnegan scoring system: Novel assessment and diagnostic techniques for the opioid-exposed infant. Semin Fetal Neonatal Med. 2019;24(2):115-120. doi:10.1016/j.siny.2019.01.003
 de Lisser R. Trauma Informed Care Bringing healing to the center of care. Accessed December 3, 2023. https://www.careinnovations.org/wp-content/uploads/Final_-Rosalind-DeLisser-_-Trauma-Informed-Care-PPT-8.30.pdf
University of Washington. Opioid trends across Washington state. adai.washington. Published October 12, 2023. Accessed December 1, 2023. https://adai.washington.edu/WAdata/deaths.htm
