# Supplementary material for: Training and Integration of Eat, Sleep, Console Model for Infants and Families at an Urban Academic Health Center
Source: MedEdPORTAL. 2026 Mar 19;22:11583. doi: 10.15766/mep_2374-8265.11583 (PMC12999543; doi:10.15766/mep_2374-8265.11583)
Supplement: Supplementary file 1 — Eat, Sleep, Console Algorithm.docxEat, Sleep, Console Education.pptxPre- and Postsurvey.docx [file mep_2374-8265.11583-s001.zip › C. Pre- and Postsurvey.docx]

Appendix C: Pre-and Post-Eat, Sleep, Console Education Survey

Demographic Questions:

1. What is your age?
   1. 18-24
   2. 25-34
   3. 35-44
   4. 45-54
   5. 55-64
   6. 65-74
   7. 75+
2. Which gender(s) do you identify most with? (select all that apply)
   1. Cisgender woman
   2. Cisgender man
   3. Transgender woman/trans woman/male to female
   4. Transgender man/trans man/female to male
   5. Genderqueer
   6. Non-binary
   7. Prefer not to say
   8. None of the above
3. What race(s) best describes you? (select all that apply)
   1. American Indian or Alaska native
   2. Asian
   3. Black or African American
   4. Native Hawaiian or Other Pacific Islander
   5. White
   6. Prefer not to say
   7. None of the above
4. Are you of Hispanic or Latino origin?
   1. Yes
   2. No
   3. Prefer not to say
5. What specialty do you primarily work in? (select all that apply)
   1. Family Medicine
   2. Lactation consultant
   3. Midwives
   4. Neonatology/pediatrics
   5. Nursing
   6. Obstetrics and Gynecology
   7. Social work
   8. Other
6. How many years have you worked in the specialty you selected in the previous question?
   1. 0-5
   2. 6-10
   3. 11-20
   4. 21-30
   5. 31-40
   6. 41-50
   7. 50+
7. Have you received any training on the Eat, Sleep, Console approach to management of opioid exposed newborns?
   1. Yes
   2. No
8. Have you had prior experience delivering the Eat, Sleep, Console approach of care to newborns and families?
   1. Yes
   2. No
9. What are the last 4 digits of your phone number AND the first letter of your last name? (e.g. if your name is Jane Doe and your phone number is 206-744-1234, you would reply 1234D) This information will be used to anonymously link your pre-training and post-training survey responses.

|  | Before/After completing this training… | 1 Strongly disagree | 2  Disagree | 3 Neutral | | 4  Agree | 5 Strongly agree |
| --- | --- | --- | --- | --- | --- | --- | --- |
|  | I feel prepared to incorporate the principles of ESC into my work |  |  | |  |  |  |
|  | I feel prepared to assess the effects of neonatal withdrawal symptoms using the ESC tool |  |  | |  |  |  |
|  | I feel prepared to implement strategies to promote parent and infant togetherness and care whenever possible |  |  | |  |  |  |
|  | I feel prepared to support parental involvement in decisions related to their infant’s care |  |  | |  |  |  |
|  | I feel prepared to use nonpharmacologic interventions when caring for an infant with NOWS |  |  | |  |  |  |
|  | I believe the ESC score that I assign to infants with NOWS is accurate and objective |  |  | |  |  |  |
|  | I believe the best place for infants with NOWS to be cared for is the NCU or NICU |  |  | |  |  |  |
|  | I believe that parents of infants with NOWS cannot provide adequate care to their infants |  |  | |  |  |  |
|  | I encourage parents of infants with NOWS to breast/chest feed if not otherwise contraindicated |  |  | |  |  |  |

The survey was adapted by the author from Romisher et al.^1^, de Graca et al.^2^, and Nota Bene et al.^3^

References

1. Romisher R, Hill D, Cong X. Neonatal Abstinence Syndrome: Exploring Nurses’ Attitudes, Knowledge, and Practice. *Adv Neonatal Care*. 2018;18(2):E3-E11. doi:10.1097/ANC.0000000000000462

2. de Graca M. *Implementation of Eat, Sleep, Console Approach to Care for Opioid Exposed Newborns*. 2021. http://hdl.handle.net/10713/15792

3. Nota Bene Consulting Group, BC Women’s Hospital and Health Center, Provincial Health Services Authority. Implementing Eat Sleep Console. bcwomens. June 2022. Accessed October 27, 2023. http://www.bcwomens.ca/Professional-Resources-site/Documents/PHSA_implementing%20eat-sleep-console-vFIN.pdf
