# Supplementary material for: Training and Integration of Eat, Sleep, Console Model for Infants and Families at an Urban Academic Health Center
Source: MedEdPORTAL. 2026 Mar 19;22:11583. doi: 10.15766/mep_2374-8265.11583 (PMC12999543; doi:10.15766/mep_2374-8265.11583)
Supplement: Supplementary file 1 — Eat, Sleep, Console Algorithm.docxEat, Sleep, Console Education.pptxPre- and Postsurvey.docx [file mep_2374-8265.11583-s001.zip › _Educational Summary Report_11583.pdf]

MedEdPORTAL
THE AAMC JOURNAL OF TEACHING AND LEARNING RESOURCES

Original Publication Open Access

Training and Integration of Eat, Sleep, Console Model for Infants and Families at an Urban Academic Health Center

Sydney Conti, DNP, FNP-BC, Jennifer Chin, MD, MS, Katherine Kemble, DNP, FNP-C, Bethany Rolfe Witham, DNP, FNP-BC, Katelyn Yoder, MD, MSc, Amber Pattison, MSN, Ying Zhang, MD, MPH*

*Corresponding author: zhangy07@uw.edu

Abstract

Introduction: Eat, Sleep, Console (ESC) is an effective approach for evaluating and managing neonatal opioid withdrawal syndrome (NOWS). The current standard, Finnegan Neonatal Abstinence Scoring System, requires waking neonates to assess NOWS and prioritizes pharmacotherapy treatment. However, ESC focuses on infants' abilities to function and cope with opioid withdrawal, prioritizes nonpharmacologic interventions, and emphasizes the crucial role of the parent–infant relationship. We created and delivered ESC training for perinatal and neonatal staff and clinicians across an urban academic health center. **Methods:** We utilized the knowledge-to-action framework to guide project design and implementation. The training program consisted of 30- to 60-minute didactic sessions for neonatal and perinatal clinicians and staff on labor and delivery and neonatal intensive care units, an ESC algorithm for care, and pre- and posttraining surveys. **Results:** We trained 254 participants (nurses, OB/GYN, and family medicine attending physicians and residents, neonatal advanced practice clinicians, midwives, social workers) through virtual educational sessions. Eighty-eight participants completed pre- or posttraining surveys, and 11 completed both surveys. Posttraining results demonstrated statistically significant improvement in self-rated preparedness to use nonpharmacologic interventions (mean score 3.91 vs. 4.64, pre- vs. posttraining paired surveys on 5-point scale [1 = *strongly disagree*, 5 = *strongly agree*]; $p = .03$;). Pre/posttraining unpaired survey results indicated high levels of preparedness implementing ESC concepts. **Discussion:** ESC education enhanced preparedness of birthing staff and clinicians to implement the nonpharmacologic ESC tool for management of NOWS. Coordinated, multidisciplinary education and collaboration support the successful implementation of ESC in clinical settings.

Keywords
Substance Use Disorders, Neonatal Opioid Withdrawal, Neonatal-Perinatal Medicine, OB/GYN

Educational Objectives

By the end of this activity, learners will be able to:

1. Incorporate the principles of the Eat, Sleep, Console (ESC) approach to care for the evaluation and management of neonatal opioid withdrawal syndrome (NOWS).
2. Assess the effects of neonatal withdrawal symptoms using the ESC tool.
3. Construct a plan to use nonpharmacologic interventions, including ESC, when caring for newborns with NOWS.

Citation:
Conti S, Chin J, Kemble K, et al. Training and integration of eat, sleep, console model for infants and families at an urban academic health center. *MedEdPORTAL*. 2026;22:11583.
https://doi.org/10.15766/mep_2374-8265.11583

Introduction

Opioid use during pregnancy has drastically increased in the US over the past decade and affects pregnant people of all racial, ethnic, socioeconomic, and geographic populations.[1,2] Between 2010 and 2017, pregnant persons with opioid-related diagnoses increased by 131%, with an associated 82% increase in neonatal opioid withdrawal syndrome (NOWS).[2,3] About 50%–90% of infants exposed to opioids in utero will develop NOWS after birth.[1,4,5] NOWS symptoms are expressed on a spectrum and affect dysregulation in attention, motor and tone control, sensory processing, autonomic instability, central nervous system hyperirritability, and gastrointestinal dysfunction.[1,6] In the US, infants with NOWS have a longer hospital stay, averaging 10.8 days, compared to infants without NOWS, averaging a stay of 1.6 days, and the associated estimated mean cost of hospitalization is higher for infants with NOWS (averaging $14,600 more than for infants without NOWS).[3]

Copyright © 2026 Conti et al. This is an open-access publication distributed under the terms of the Creative Commons Attribution license.

The most well-known and globally utilized NOWS evaluation tool is the Finnegan Neonatal Abstinence Scoring System (FNASS), but the lack of tool validity, interrater reliability, and overestimation of the need for pharmacologic management of opioid withdrawal underscore a need for hospitals and clinicians to change their approach to monitoring and responding to NOWS.[7-13] Eat, Sleep, Console (ESC), designed in 2014, is a newer approach to evaluation and management of NOWS and has demonstrated improved neonatal outcomes and lower costs of care.[7] ESC is 99.4% more sensitive than FNASS in identifying infants needing pharmacologic treatment.[11] Infants managed with the ESC model have shorter hospital stays, fewer pharmacologic intervention requirements, improved breast/chest feeding rates, and decreased cost to the health care system compared to the FNASS model of care.[7-12,14-16] ESC focuses on the infant's ability to function and cope with opioid withdrawal, prioritizes nonpharmacologic interventions as the first-line treatment, and emphasizes the parent–infant dyad.[7] ESC is both a tool and a cultural shift that reframes the role of the parent/caregiver to be both integral support and therapeutic participant in the care of infants with NOWS.

Although several published articles have focused on the implementation of the ESC approach to care, there is little medical education literature and no literature within *MedEdPORTAL* detailing and evaluating training and education of perinatal and neonatal staff and providers to provide the ESC model of care.[4,17] While the staff and clinician roles primarily responsible for monitoring and evaluating ESC are nursing and neonatal providers, all staff and providers caring for birthing parents and their families on labor and delivery and within neonatal and neonatal intensive care units should be familiar with the ESC approach to care in order to provide accurate and consistent education and support for nonpharmacologic management of NOWS to caregivers. Hence, at our urban academic hospital, we designed and implemented a training program using didactic and case-based learning to deliver ESC education to multidisciplinary staff, providers, and trainees working in labor and delivery and neonatal hospital units. We designed the ESC educational training as didactics followed by skills practice developed from the framework of case-based learning, which engages learners in collaborative learning, helping them develop clinical reasoning and understanding from newly gained knowledge and contextualized concrete examples.[18] This education and training were part of a larger focus on improving overall perinatal substance use disorder (SUD) care for individuals and their families at our institution.

Methods

Setting and Participants

We developed ESC education and training for an urban, multisite academic medical center with 2 inpatient labor and delivery units (University of Washington Medical Center [UWMC]): Northwest campus and Montlake campus. Sydney Conti, a doctor of nursing practice (DNP) candidate at the time of curriculum development, led the training development and plan as part of her capstone DNP project, with guidance from a team of OB/GYN and family medicine attending faculty (co-authors), nursing, lactation, and neonatal clinician leadership from the Perinatal Substance Use Disorder Collaborative Committee at UWMC. We initially piloted the ESC training at UWMC Northwest before adapting the educational training for UWMC Montlake.

A hospital-wide multidisciplinary committee, the UWMC Perinatal SUD Collaborative, focusing on improving care of individuals with SUD and their families, supported the development and implementation of the training sessions. We conducted educational trainings about the ESC model of evaluating and managing NOWS, implemented during preexisting monthly perinatal quality and safety meetings for providers and staff on labor and delivery and neonatal intensive care units, as well as during weekly resident didactics. Since the initial educational launch of ESC, our hospital systems have incorporated the didactics slides and recorded training into the onboarding education of new nursing staff and asynchronous training for new clinicians and residents.

Design and Development

The knowledge-to-action (KTA) framework guided the design and implementation of this project through the knowledge creation and action cycle phases.[19] The KTA framework, developed by Graham and colleagues in 2006,[19] supports the translation of evidence-based practice into health interventions and implementation of effective care programs. We conducted both knowledge and action cycles simultaneously and iteratively, which improved the tailoring of our educational intervention and implementation of ESC approach to care in our local context.

The knowledge creation funnel began with conducting a literature review, in which we searched for published literature that included the ESC model of care, ESC implementation techniques, ESC staff training, and barriers and facilitators of ESC uptake. We compiled an optional ESC training resource list from our literature review and from the published resources from other hospital systems. The literature review guided our creation of an ESC algorithm and education slide deck (Appendix A and

Copyright © 2026 Conti et al. This is an open-access publication distributed under the terms of the Creative Commons Attribution license.

Appendix B). We developed a pre- and posttraining survey to evaluate staff preparedness for the implementation of ESC and identify knowledge gaps that may benefit from additional focused training[20,21] (Appendix C). We adapted the survey from methods previously described by Romisher et al, da Graca et al, and the Nota Bene Consulting Group.[20-22] The survey included 5 questions about self-rated preparedness for ESC and 4 questions about trainees' beliefs and attitudes toward infants with NOWS and their parents/caregivers. We used a 5-point Likert scale for ratings in response to questions 1 through 9, with a score of 1 representing *strongly disagree* and a score of 5 representing *strongly agree*. For questions 1 through 6 and question 9, high scores were considered indicative of more informed attitudes and preparedness regarding implementation of ESC, while for questions 7 and 8, low scores were considered indicative of beliefs more aligned with the core principles of ESC.

The action cycle included monthly meetings with the Perinatal SUD Collaborative to identify knowledge gaps and adapt the slide deck to specific department needs. OB/GYN, family medicine, pediatrics, neonatology, nursing, and lactation clinician leads conducted virtual and in-person ESC education trainings using the created didactics slide deck, which they presented to their respective providers and staff members. Based on the KTA framework and discussions identifying facilitators and barriers to implementation of ESC, the Perinatal SUD Collaborative Committee identified a need to conduct trainings in interprofessional groups (e.g., OB/GYN, midwives, family medicine, and nursing together in a single training) and in separate professional groups (e.g., neonatal providers, nursing staff, and family medicine residents) on multiple occasions, in order to reach as many providers and staff as possible on the labor and delivery unit prior to the launch of the ESC clinical protocol.

The duration of each educational session was ~30–60 minutes and included 1–4 facilitators/instructors. Facilitators of the trainings had content knowledge from clinical practice and completed the University of British Columbia's Perinatal Substance Use e-learning modules, including 1 module focused on care of newborns exposed to substances during pregnancy with a focus on ESC.[23] The target audience for the trainings included all clinicians and staff delivering care to birthing parents, neonates, and their families affected by SUD/opioid use disorder, which included OB/GYN and family medicine providers and residents within these professions, as well as midwives, lactation consultants, antepartum and postpartum nurses, social workers, and neonatal advanced practice clinicians. Didactic sessions were limited to 30 minutes for family medicine and OB/GYN resident trainees as part of their regularly scheduled weekly didactic sessions. Training for nursing staff, neonatal staff, and interprofessional attending clinicians during the monthly perinatal safety and quality meetings lasted 60 minutes. All training occurred between January 1 and March 1, 2024.

All slide deck materials and speaker notes for training facilitators are included in Appendix B. The slide deck features a case-based scenario designed to actively engage learners with the material. We presented cases as part of the didactic session, which prompted learners as a large group to practice using the ESC scoring system for theoretical clinical scenarios of newborns exhibiting symptoms of NOWS. The slide deck also included 3 knowledge check questions to solidify knowledge comprehension of ESC. We administered pre- and posttraining surveys electronically using RedCap to all participants in the various ESC trainings. The surveys assessed trainees' self-rated preparedness to implement ESC and beliefs and attitudes toward infants with NOWS and their parents/caregivers.[24,25]

Data Analysis

We collected and analyzed pre- and posttraining survey data. Descriptive statistics were calculated for demographic characteristics of participants. The treatment effect of the ESC education training was calculated using the difference in mean scores for each question, and statistical significance was determined using a paired-sample t test. A second analysis was conducted using Welch's t test for the nonpaired data set, included 54 pretraining and 22 posttraining records. Results comparing pre- and posttraining survey data were evaluated at a significance level of $p < .05$. This educational innovation plan was reviewed by the University of Washington Institutional Review Board (IRB) and does not qualify as human subjects research (IRB no. STUDY00024329).

Results

A total of 254 participants attended the various ESC training sessions. Eighty-eight survey responses, with self-ratings on a 5-point Likert scale, were collected, representing a 35% response rate. One response was incomplete, and 12 responses were excluded from the analysis due to the inability to determine whether they were pre- or posttraining. After exclusions, 54 unique participants completed the pretraining survey, and 22 unique participants completed the posttraining survey, with 11 participants completing both the pre- and posttraining surveys.

The majority of staff who responded to the survey identified as White (82%), non-Hispanic or Latino (84%), cisgender woman (86%), ages 25–44 years (70%), employed at inpatient sites on

Copyright © 2026 Conti et al. This is an open-access publication distributed under the terms of the Creative Commons Attribution license.

the UWMC Northwest campus (98%), and specialized in nursing (63%), family medicine (16%), or OB/GYN (26%). Table 1 lists the demographic characteristics of the survey respondents, and Table 2 presents the results of paired and unpaired analyses of the pre- and posttraining survey ratings.

Table 1. Demographics of All Participants in the ESC Approach to Care of Infants With NOWS Educational Training (*N* = 88)

Demographics	*n* (%)
Age (years)	
18-24	3 (3)
25-34	34 (39)
35-44	28 (32)
45-54	14 (16)
>55	9 (10)
Gender[a]	
Cisgender woman	75 (86)
Cisgender man	6 (7)
Transgender (MTF or FTM)	0 (0)
Nonbinary	1 (1)
Prefer not to say	4 (5)
None of the above	1 (1)
Race	
American Indian or Alaska Native	3 (3)
Asian	13 (15)
Black or African American	1 (1)
Native Hawaiian or Other Pacific Islander	0 (0)
White	71 (82)
Prefer not to say	2 (2)
None of the above	2 (2)
Ethnicity	
Hispanic or Latino	12 (14)
Non-Hispanic or Latino	74 (84)
Prefer not to say	2 (2)
Workplace location[b]	
UWMC Northwest	86 (98)
UWMC Montlake	5 (6)
UWMC Outpatient Clinics	4 (5)
None of the above	1 (1)
Specialty	
Family medicine	14 (16)
Lactation consultant	1 (1)
Midwives	0 (0)
Neonatology/pediatrics	1 (1)
Nursing	55 (63)
Obstetrics and gynecology	23 (26)
Social work	0 (0)
Other	1 (1)
Work experience in indicated specialty (years)	
0-5	31 (35)
6-10	17 (19)
11-20	26 (30)
21-30	9 (10)
>31	5 (6)
Received previous training on ESC	
Yes	65 (74)
No	23 (26)
Prior experience delivering ESC	
Yes	28 (32)
No	60 (68)

Abbreviations: ESC, Eat, Sleep, Console model; FTM, female-to-male; MTF, male-to-female; NOWS, neonatal opioid withdrawal syndrome; UWMC, University of Washington Medical Center.
[a]*N* = 87 due to 1 incomplete record for gender.
[b]Respondents could choose multiple answers if applicable.

Data Analysis of Survey Responses

Paired data: In responding to question 5, “I feel prepared to use nonpharmacologic interventions when caring for an infant with NOWS,” there was a statistically significant increase in participants’ scores, from a mean score of 3.91 pretraining to 4.64 posttraining ($p = .03$), indicating that this ESC education training may enhance trainees’ self-perceived preparedness to administer nonpharmacologic interventions. The remaining 8 questions did not exhibit statistically significant score changes from pre- to posttraining, but the change in mean scores moved in the predicted directions, with scores increasing for questions 1–6 and question 9, and scores decreasing for questions 7 and 8 (Table 2).

Unpaired data: In responding to questions 1, 3, and 5, participants’ mean scores were found to statistically significantly increase from pre- to posttraining. In the posttraining survey, staff self-ratings indicated an increased preparedness to incorporate the principles of ESC into their work (mean score 3.89 pretraining vs. 4.27 posttraining; $p = .03$), to implement strategies to promote parent/caregiver and infant togetherness and care whenever possible (mean score 4.06 pretraining vs. 4.45 posttraining; $p = .02$), and to use nonpharmacologic interventions when caring for an infant with NOWS (mean score 4.04 pretraining vs. 4.55 posttraining; $p = .004$) (Table 2).

Discussion

Opioid use in pregnancy and the incidence of NOWS are both increasing in the US and require compassionate, patient-centered care.[1-4] We demonstrate that a combination of live and recorded training sessions, including case study examples and opportunities to practice ESC scoring for neonates experiencing NOWS, were used to effectively provide education to staff in our urban academic hospital system prior to a hospital-wide initiation of the ESC approach to care. We found that staff felt prepared for implementation and integration of ESC for evaluation and management of NOWS after our training. Among the paired data, 8 of the 9 questions did not exhibit statistically significant changes in scores; however, notably the pretraining scores reflected high levels of preparedness with the material. In both the pre- and posttraining analysis of the paired and nonpaired data, staff continued to score high on questions 1 through 6 and question 9 (questions generally assessing self-reported preparedness to implement ESC approaches to care of newborns with NOWS), while scoring low for questions 7 and 8 (questions assessing attitudes about care of newborns exposed to substances), which, based on these evaluations, reflects favorable attitudes for promoting newborn and caregiver

Copyright © 2026 Conti et al. This is an open-access publication distributed under the terms of the Creative Commons Attribution license.

Table 2. Survey Results for Paired and Unpaired Data Before and After Participation in the ESC Approach to Care of Infants With NOWS Educational Training

	Paired Responses			Unpaired Responses		
**Survey Question**[a]	**Pre *M* (*N* = 11)**	**Post *M* (*N* = 11)**	***P***	**Pre *M* (*N* = 54)**	**Post *M* (*N* = 22)**	***P***
1. I feel prepared to incorporate the principles of ESC into my work.	3.73	4.27	.11	3.89	4.27	.03[b]
2. I feel prepared to assess the effects of neonatal withdrawal symptoms using the ESC tool.	4.00	4.27	.39	3.90	4.23	.11
3. I feel prepared to implement strategies to promote parent and infant togetherness and care whenever possible.	4.09	4.64	.11	4.06	4.45	.02[b]
4. I feel prepared to support parental involvement in decisions related to their infant's care.	3.91	4.18	.47	4.06	4.23	.36
5. I feel prepared to use nonpharmacologic interventions when caring for an infant with NOWS.	3.91	4.64	.03[b]	4.04	4.55	.004[c]
6. I believe the ESC score assigned to infants with NOWS is accurate and objective.	4.09	4.45	.10	4.22	4.22	.98
7. I believe the best place for infants with NOWS to be cared for is the NCU or NICU.	2.36	2.27	.76	2.52	2.55	.92
8. I believe that parents of infants with NOWS cannot provide adequate care to their infants.	1.81	1.72	.78	1.87	2.18	.26
9. I encourage parents of infants with NOWS to breast/chest feed if not otherwise contraindicated.	4.09	4.55	.24	4.37	4.41	.82

Abbreviations: ESC, Eat, Sleep, Console model; NCU, neonatal care unit; NICU, neonatal intensive care unit; NOWS, neonatal opioid withdrawal syndrome.
[a]Rated on a 5-point Likert scale (1 = *Strongly disagree*, 5 = *Strongly agree*).
[b]Significant at $p < .05$.
[c]Significant at $p < .005$.

bonding and NOWS care outside of neonatal/intensive care units provided by the newborn's primary caregivers when appropriate.

Our findings suggest that after this intervention, providers and staff were more prepared to utilize nonpharmacologic interventions and to incorporate ESC principles and strategies in their care of newborns with NOWS. The absence of additional demonstrated impact may reflect the respondents' high level of preexisting preparedness and proficiency in this topic. This could be a result of previously delivered training with the staff, clinicians, and trainees at UWMC, which was focused on trauma-informed care for perinatal patients and had a special focus on perinatal patients with SUD and their families. Ultimately, these are desired results in which staff beliefs and attitudes are aligned with the core principles of ESC and an overall high self-rated preparedness to implement aspects of ESC.

The results are similar to other studies of ESC training in the clinical workplace in demonstrating high levels of self-rated preparedness to implement ESC after receiving education.[22] While Romisher and colleagues specifically surveyed nurses' beliefs and attitudes toward infants with NOWS and their families without an associated training, our educational innovation was expanded to include multiple health care disciplines and multidisciplinary trainings.[20] The Nota Bene Consulting Group surveyed providers and staff on gains in knowledge and practice of ESC after a multimodule ESC training, and da Graca and colleagues surveyed beliefs and attitudes of providers and staff toward infants with NOWS and their families prior to ESC training.[21,22] To our knowledge, our educational innovation is the first to compare paired pre- and posttraining survey data with a focus on the self-rated preparedness of providers and staff to implement ESC and their beliefs and attitudes toward infants with NOWS and their families.

After our intervention, a significant change in the approach to monitoring and managing NOWS at UWMC Northwest was implemented, with the support and leadership of our Perinatal SUD Collaborative. Sustaining and monitoring a successful practice change requires resources and support from key partners and interdisciplinary engagement. We have seen improvement in communication between interdisciplinary team members on the labor and delivery unit, and improved application of the principles of trauma-informed care in support of newborns and their family members who are affected by SUD. Ongoing monitoring and review of outcomes from care of neonates with NOWS using the ESC approach has created opportunities for iterative evaluation and improvement of workflows and policies for clinical care.

Strengths of our educational innovation include the incorporation of multiple professions and specialties, allowing for simulated team-based education and ensuring a shared understanding of the changes implemented on the labor and delivery and neonatal units to monitor and manage NOWS. We developed our intervention based on input from a large committee, which included collaboration from multiple specialties. Our didactic

Copyright © 2026 Conti et al. This is an open-access publication distributed under the terms of the Creative Commons Attribution license.

materials were distributed in a variety of settings to reach multiple audiences at different times.

The analysis and interpretation of the survey results were limited by a small sample size, particularly among the paired data, and the majority of respondents (98%) were from 1 primary workplace location (i.e., UWMC Northwest), which could also limit interpretation of the results. While we were successful in completing training sessions for a multidisciplinary group of trainees and clinicians and staff at the UWMC Northwest campus, the other inpatient site (i.e., UWMC Montlake) and outpatient clinic staff received training at a later date. Thus, the training of nursing staff and attending providers at the other clinical sites happened 10 months after initial dissemination of our ESC training program, and thus we did not capture survey data for other campuses.

The difference between the mean scores in the nonpaired data analysis should be interpreted cautiously because the 2 groups comprise different individuals, and baseline characteristics of the 2 groups, including specialty or background training, may impact the scores and responses. Based on the demographics of the staff who responded to the survey, it is evident that certain groups were underrepresented in the results analyses. Inclusion of feedback from midwives, neonatologists, lactation consultants, and social workers, as well as individuals of diverse ages, races, ethnicities, and genders, would enhance the generalizability of data on staff self-rated preparedness for ESC implementation. Additionally, we did not require a response to the training surveys, and thus the data we collected was from only those who chose to respond, which could introduce response bias.

Despite these limitations, our ESC education training produced beneficial results among staff, as evidenced by their high levels of self-rated preparedness to implement ESC, and helped to support the implementation of our policy change for NOWS monitoring and management in our urban academic hospital system.

Appendices

A. Eat, Sleep, Console Algorithm.docx

B. Eat, Sleep, Console Education.pptx

C. Pre- and Postsurvey.docx

All appendices are peer reviewed as integral parts of the Original Publication.

Sydney Conti, DNP, FNP-BC: Doctor of Nursing Practice Student, University of Washington School of Nursing

Jennifer Chin, MD, MS: Assistant Professor, Division of Complex Family Planning, Department of Obstetrics and Gynecology, University of Washington School of Medicine

Katherine Kemble, DNP, FNP-C: Associate Teaching Professor, Department of Child, Family, and Population Health Nursing, University of Washington School of Nursing

Bethany Rolfe Witham, DNP, FNP-BC: DNP Program Director, University of Washington School of Nursing; Associate Teaching Professor, Department of Child, Family, and Population Health Nursing, University of Washington School of Nursing

Katelyn Yoder, MD, MSc: Assistant Professor, Department of Obstetrics and Gynecology, University of Washington School of Medicine

Amber Pattison, MSN: Perinatal Clinical Nurse Specialist, University of Washington Medical Center-Northwest, Childbirth Center, University of Washington School of Medicine

Ying Zhang, MD, MPH: Associate Professor, Department of Family Medicine, University of Washington School of Medicine; ORCID: https://orcid.org/0000-0002-1846-962X

Acknowledgments
We would like to express sincere gratitude to the University of Washington Perinatal Substance Use Disorder Collaborative for their support with this initiative and manuscript and for their continued commitment to improving care for infants with neonatal opioid withdrawal syndrome and their families. Thank you to the Washington Department of Health and the Washington State Hospital Association for their continued support with this initiative. We extend our sincere gratitude to Adam Wilson, MS, for his help with the data analysis and Renee Cantarini, DNP, FNP-BC.

Disclosures
None to report.

Funding/Support
Author Sydney Conti, DNP, FNP-BC, was supported by the Health Resources and Services Administration (HRSA) of the US Department of Health and Human Services as part of an award totaling $649,982 with 0% percentage financed with nongovernmental sources. HRSA was not involved in the research, review, or writing of this manuscript.

Ethical Approval
The University of Washington Institutional Review Board reviewed this project.

References

1. Committee Opinion No. 711: opioid use and opioid use disorder in pregnancy. *Obstet Gynecol*. 2017;130:e81-e94. https://doi.org/10.1097/AOG.0000000000002235
2. *Evidence-Based, Whole-Person Care for Pregnant People Who Have Opioid Use Disorder*. SAMSHA; 2023. Accessed

Copyright © 2026 Conti et al. This is an open-access publication distributed under the terms of the Creative Commons Attribution license.

MedEdPORTAL
THE AAMC JOURNAL OF TEACHING AND LEARNING RESOURCES

February 2, 2026.
https://store.samhsa.gov/sites/default/files/pep23-02-01-002.jpg

3. Hirai AH, Ko JY, Owens PL, Stocks C, Patrick SW. Neonatal abstinence syndrome and maternal opioid-related diagnoses in the US, 2010-2017. *JAMA*. 2021;325(2):146-155. https://www.doi.org/10.1001/jama.2020.24991
4. Wortham SE, Bianchi AL. Strategies to successfully implement an eat, sleep, console protocol. *MCN Am J Matern Nurs*. 2022;47(4): 182-188. https://www.doi.org/10.1097/NMC.0000000000000833
5. Linn N, Stephens K, Swanson-Biearman B, Lewis D, Whiteman K. Implementing trauma-informed strategies for mothers of infants with neonatal abstinence syndrome. *MCN Am J Matern Nurs*. 2021;46(4):211-216. https://www.doi.org/10.1097/NMC.0000000000000728
6. Jansson LM, Patrick SW. Neonatal abstinence syndrome. *Pediatr Clin North Am*. 2019;66(2):353-367. https://www.doi.org/10.1016/j.pcl.2018.12.006
7. Grossman MR, Berkwitt AK, Osborn RR, et al. An initiative to improve the quality of care of infants with neonatal abstinence syndrome. *Pediatrics*. 2017;139(6):e20163360. https://www.doi.org/10.1542/peds.2016-3360
8. Dodds D, Koch K, Buitrago-Mogollon T, Horstmann S. Successful implementation of the eat sleep console model of care for infants with NAS in a community hospital. *Hosp Pediatr*. 2019;9(8): 632-638. https://www.doi.org/10.1542/hpeds.2019-0086
9. Grisham LM, Stephen MM, Coykendall MR, Kane MF, Maurer JA, Bader MY. Eat, sleep, console approach: a family-centered model for the treatment of neonatal abstinence syndrome. *Adv Neonatal Care*. 2019;19(2):138-144. https://www.doi.org/10.1097/ANC.0000000000000581
10. Haaland G, Kunkel M, Nguyen CM, Wonder AH. Using the eat sleep console model to promote optimal care and outcomes for infants with neonatal abstinence syndrome: a nurse-driven, multidisciplinary initiative. *Adv Neonatal Care*. 2023;23(4):320-329. https://www.doi.org/10.1097/ANC.0000000000001028
11. Nicholson S, Waskosky A. The eat, sleep, console method: A literature review. *Neonatal Netw*. 2022;41(6):333-340. https://www.doi.org/10.1891/NN-2021-0003
12. Young LW, Ounpraseuth ST, Merhar SL, et al; ACT NOW Collaborative. Eat, sleep, console approach or usual care for neonatal opioid withdrawal. *N Engl J Med*. 2023;388(25): 2326-2337. https://www.doi.org/10.1056/NEJMoa2214470
13. Schiff DM, Grossman MR. Beyond the Finnegan scoring system: novel assessment and diagnostic techniques for the opioid-exposed infant. *Semin Fetal Neonatal Med*. 2019;24(2): 115-120. https://doi.org/10.1016/j.siny.2019.01.003
14. Miller PA, Willier T. Baby STRENGTH: eat, sleep, console for infants with neonatal abstinence syndrome. *Adv Neonatal Care*. 2021;21(2):99-106. https://www.doi.org/10.1097/ANC.0000000000000840
15. Wachman EM, Houghton M, Melvin P, et al. A quality improvement initiative to implement the eat, sleep, console neonatal opioid withdrawal syndrome care tool in Massachusetts' PNQIN collaborative. *J Perinatol*. 2020;40(10):1560-1569. https://www.doi.org/10.1038/s41372-020-0733-y
16. Ryan K, Moyer A, Glait M, et al. Correlating scores but contrasting outcomes for eat sleep console versus modified Finnegan. *Hosp Pediatr*. 2021;11(4):350-357. https://www.doi.org/10.1542/hpeds.2020-003665
17. Gallant SM, DeCoste K, Norris N, et al. Implementing the eat, sleep, console model of care: A scoping review. *Hosp Pediatr*. 2025;15(3):e108-e120. https://doi.org/10.1542/hpeds.2024-008078
18. Thistlethwaite JE, Davies D, Ekeocha S, et al. The effectiveness of case-based learning in health professional education. *A BEME systematic review: BEME Guide No. 23. Med Teach.* 2012; 34(6):e421-e444. https://www.doi.org/10.3109/0142159X.2012.680939
19. Graham ID, Logan J, Harrison MB, et al. Lost in knowledge translation: time for a map? *J Contin Educ Health Prof*. 2006;26(1):13-24. https://www.doi.org/10.1002/chp.47
20. Romisher R, Hill D, Cong X. Neonatal abstinence syndrome: exploring nurses' attitudes, knowledge, and practice. *Adv Neonatal Care*. 2018;18(2):E3-E11. https://www.doi.org/10.1097/ANC.0000000000000462
21. da Graca MM. *Implementation of Eat, Sleep, Console Approach to Care for Opioid Exposed Newborns*. NDP project. University of Maryland, Baltimore; 2021. Accessed February 2, 2026. http://hdl.handle.net/10713/15792
22. *Implementing Eat Sleep Console*. Nota Bene Consulting Group, BC Women's Hospital and Health Center, Provincial Health Services Authority; 2022. Accessed February 2, 2026. http://www.bcwomens.ca/Professional-Resources-site/Documents/PHSA_implementing%20eat-sleep-console-vFIN.pdf
23. Perinatal substance use. University of British Columbia Continuing Professional Development. Accessed February 2, 2026. https://ubccpd.ca/learn/learning-activities/course?eventtemplate=41
24. Harris PA, Taylor R, Thielke R, Payne J, Gonzalez N, Conde JG. Research electronic data capture (REDCap)—A metadata-driven methodology and workflow process for providing translational research informatics support. *J Biomed Inform.* 2009;42(2): 377-381. https://doi.org/10.1016/j.jbi.2008.08.010
25. Harris PA, Taylor R, Minor BL, et al; REDCap Consotrium. The REDCap consortium: Building an international community of software partners. *J Biomed Inform.* 2019;95:103208. https://www.doi.org/10.1016/j.jbi.2019.103208

Received: November 17, 2025
Accepted: January 14, 2026
Published: March 19, 2026

Copyright © 2026 Conti et al. This is an open-access publication distributed under the terms of the Creative Commons Attribution license.
